# Lipids and Terpenoids from the Deep-Sea Fungus *Trichoderma lixii* R22 and Their Antagonism against Two Wheat Pathogens

**DOI:** 10.3390/molecules28176220

**Published:** 2023-08-24

**Authors:** Chang-Peng Li, Zhen-Zhen Shi, Sheng-Tao Fang, Yin-Ping Song, Nai-Yun Ji

**Affiliations:** 1 Yantai Institute of Coastal Zone Research, Chinese Academy of Sciences, Yantai 264003, China; cpli@yic.ac.cn (C.-P.L.); zzshi@yic.ac.cn (Z.-Z.S.); stfang@yic.ac.cn (S.-T.F.); ypsong@yic.ac.cn (Y.-P.S.); 2 University of Chinese Academy of Sciences, Beijing 100049, China; 3 Shandong Saline-Alkaline Land Modern Agriculture Company, Dongying 257345, China

**Keywords:** *Trichoderma*, lipid, terpenoid, butenolide, brasilane, fungicide

## Abstract

Five new lipids, tricholixins A–E (**1**–**5**), and two known terpenoids, brasilane A (**6**) and harzianone A (**7**), were discovered from a deep-sea strain (R22) of the fungus *Trichoderma lixii* isolated from the cold seep sediments of the South China Sea. Their structures and relative configurations were identified by meticulous analysis of MS and IR as well as NMR data. The absolute configuration of **5** was ascertained by dimolybdenum-induced ECD data in particular. Compounds **1** and **2** represent the only two new butenolides from marine-derived *Trichoderma*, and they further add to the structural diversity of these molecules. Although **6** has been reported from a basidiomycete previously, it is the first brasilane aminoglycoside of *Trichoderma* origin. During the assay against wheat-pathogenic fungi, both **1** and **2** inhibited *Fusarium graminearum* with an MIC value of 25.0 μg/mL, and **6** suppressed *Gaeumannomyces graminis* with an MIC value of 12.5 μg/mL. Moreover, the three isolates also showed low toxicity to the brine shrimp *Artemia salina*.

## 1. Introduction

As one of the main grains, wheat (*Triticum aestivum* L.) is cultivated worldwide and taken as a staple food by over 35% of the global population [1]. Although the current wheat production (>700 million tons per year) is increasing all over the world, many pathogenic fungi can threaten wheat growth and lead to 15–20% yield losses every year [2]. Among the fungal pathogens, *Fusarium graminearum* is the causal agent of *Fusarium* head blight that occurs at floret and glume parts of wheat [3]. This wheat disease has ever outbroken in Asia, Europe, North America, and South America and resulted in devastating economic losses [2,3]. As a wheat-rhizospheric pathogen, the soil-borne fungus *Gaeumannomyces graminis* can give rise to the notorious take-all disease. The infection may result in stunting and premature ripening, with the observed symptoms inclusive of dark roots, yellow leaves, white heads, and shrivelled grains [4]. *Fusarium* head blight and take-all disease can decrease both the yield and quality of wheat and their pathogens also have the ability to infect other grains, such as barley and oats. Besides the alteration of tillage practices, synthesized fungicides have been widely used to control these diseases. However, their efficacy has been decreased by the emergence of drug resistance due to long-time utilization [5]. Natural antibiotics that are more ecologically and environmentally preferable than synthesized ones have been continuously discovered [6].

**Figure 1:**
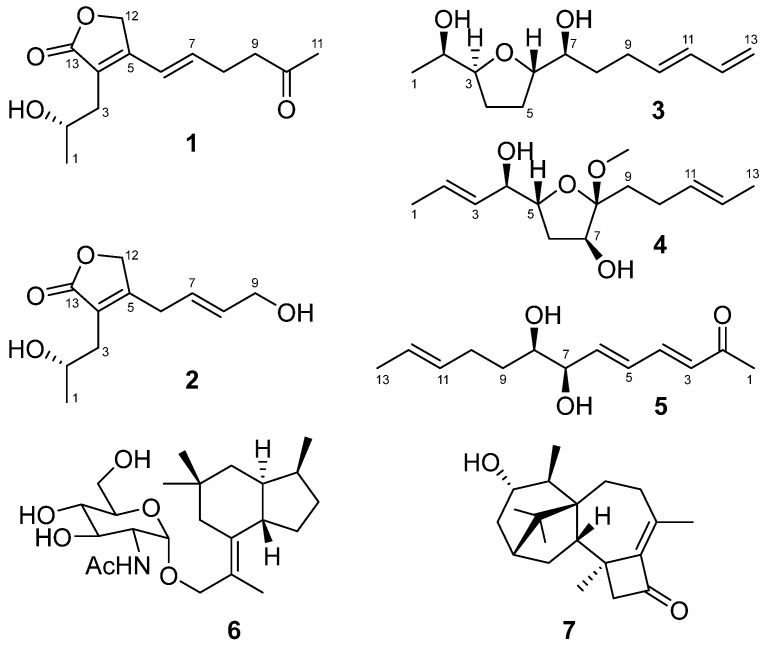
Chemical structures of **1**–**7**.

*Trichoderma* species have achieved great success to antagonize plant-pathogenic fungi in agriculture [7,8]. A number of fungicidal metabolites, such as 6-pentyl-2*H*-pyran-2-one, viridin, and trichodermin, have been isolated and identified from *Trichoderma* species of terrestrial and marine origin [7,8,9,10]. Recently, deep-sea *Trichoderma* fungi have attracted the attention of some researchers, and three unidentified strains have been chemically surveyed [11,12,13,14]. Although a total of 18 new metabolites have been obtained from these deep-sea *Trichoderma* strains, no antagonism against plant-pathogenic fungi has been detected. During our research on functional metabolites from deep-sea fungi [15,16,17], another isolate designated *Trichoderma lixii* R22 was obtained from a cold-seep sediment sample (−1217 m) collected in May 2020. Chemical investigation of this strain led to the discovery of five new lipids (**1**–**5**) and two known terpenoids (**6** and **7**) (Figure 1).

## 2. Results and Discussion

The organic extracts of deep-sea fungus *Trichoderma lixii* R22 isolated from the cold seep sediments of the South China Sea were subjected to a series of column chromatography, Sephadex LH-20, preparative TLC, and semipreparative HPLC to yield five new lipids, tricholixins A–E (**1**–**5**), and two known terpenoids, brasilane A (**6**) and harzianone A (**7**) (Figure 1).

### 2.1. Structural Elucidation

**Figure 2:**
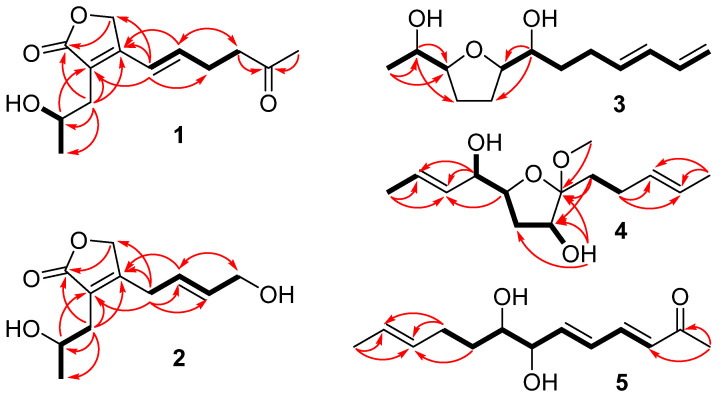
Key COSY and HMBC correlations of **1**–**5** (bold lines for COSY and arrows for HMBC).

Tricholixin A (**1**) has a molecular formula of C_13_H_18_O_4_, which was deduced from the deprotonated molecular ion peak at *m*/*z* 237.1123 in negative HRESI(−)MS. The IR absorption bands at 3440 and 1738 cm^−1^ demonstrated the presence of hydroxy and carbonyl groups. The ^1^H NMR spectrum, recorded in DMSO-*d*_6_ (Table 1), showed a methyl (C-1) doublet at δ_H_ 1.02 and a hydroxy doublet at δ_H_ 4.63, both of which exhibited COSY correlations with a multiplet at δ_H_ 3.77 for a methine group (C-2). This information defined a hydroxyethyl group, which was elongated to C-3 to form a 2-hydroxypropyl unit based on the COSY correlation between H-2 and H-3b and the HMBC correlations from H-3b to C-1 and C-2 (Figure 2). On the other hand, a *trans* double bond at C-6 was established by the chemical shifts of H-6 (δ_H_ 6.54) and H-7 (δ_H_ 6.14) and their mutually coupling constant (*J* = 16.1 Hz), and an acetonyl group was defined by the deshielded chemical shift of C-10 (δ_C_ 207.4) and its HMBC correlations with H-9 and H-11. These two parts were linked together with a methylene group (C-8) to form a 5-oxohexenyl group based on the HMBC correlations from H-6 to C-8 and from H-7 to C-9. The remaining ^1^H and ^13^C NMR signals, especially in CDCl_3_ (Table 1 and Table 2), corresponded to an α,β-unsaturated γ-lactone ring by comparison with harzianolide [18,19], and its connectivity with the 2-hydroxypropyl and 5-oxohexenyl groups was confirmed by the HMBC correlations from H-3b to C-4 and from H-6 to C-5. The above evidence established the planar structure of **1**, verified by other COSY and HMBC correlations as shown in Figure 2. C-2 is the only chiral center in this molecule, and its absolute configuration was deduced to be 2*S* by comparison of the specific rotation value with that of harzianolide ([α]^D^ 6.6) [18]. Thus, compound **1** was named (*S*,*E*)-3-(2-hydroxypropyl)-4-(5-oxohex-1-en-1-yl)furan-2(5*H*)-one.

The molecular formula of tricholixin B (**2**) was determined to be C_11_H_16_O_4_, two fewer carbon atoms than for **1**, on the basis of HRESI(+)MS data. The hydroxy and carbonyl groups were indicated by the IR absorption bands at 3430 and 1740 cm^−1^, similar to those of **1**. The ^1^H and ^13^C NMR data (Table 1 and Table 2) resembled those for **1**, but some signals lacked or altered. Analysis of those data revealed that a hydroxymethyl group (C-9), instead of an acetonyl group in **1**, should be present in **2**. This functionality was linked to a methylene group (C-6) via a *trans* double bond to form a 4-hydroxy-2-butenyl group, as seen from the large coupling constant (*J* = 15.3) between H-7 and H-8 and the COSY correlations of H_2_-9/H-8/H-7/H_2_-6 (Figure 2). The remaining NMR signals were identical to those of **1**, suggesting the same connectivity of corresponding parts in **1** and **2**. The whole structure was further validated by other COSY and HMBC correlations as shown in Figure 2. Finally, a 2*S* configuration was assigned to C-2 by the identical specific rotation value with that of **1**. Compound **2** was named (*S*,*E*)-4-(4-hydroxybut-2-en-1-yl)-3-(2-hydroxypropyl)furan-2(5*H*)-one.

HRESI(+)MS analysis gave tricholixin C (**3**) a molecular formula of C_13_H_22_O_3_, with three degrees of unsaturation. The high-frequency chemical shifts at δ_H_ 4.9–6.4 and δ_C_ 115–139 in ^1^H and ^13^C NMR spectra (Table 3 and Table 4), respectively, along with their coupling constants and COSY correlations (Figure 2), indicated the presence of a butadienyl group (C-10 to C-13), accounting for two degrees of unsaturation. To satisfy the remaining one degree of unsaturation, this compound should contain a ring unit. Four oxygenated methine groups (C-2, C-3, C-6, and C-7) were proposed by the four ^1^H NMR signals at δ_H_ 3.5–3.9 and the four ^13^C NMR signals at δ_C_ 70–85. However, only three oxygen atoms existed in the molecular formula. To match the above data, one cycloether ring and two hydroxy groups were suggested in the molecule, and the hydroxy groups were supported by the IR absorption at 3425 cm^−1^. A comparison of the NMR signals for oxygenated methine groups with literature data revealed the presence of a 2,5-dihydroxymethyltetrahydrofuran unit [20], which was supported by the COSY correlations between H-2 and H-3 and between H-6 and H-7 and the HMBC correlations from H-2 to C-3 and C-4 and from H-7 to C-5 and C-6. One terminal was methylated according to the COSY correlation between H_3_-1 and H-2 and the HMBC correlations from H_3_-1 to C-2 and C-3, and the other was linked to the butadienyl group via an ethylene group based on the COSY correlations of H-7/H_2_-8/H_2_-9/H-10. The above information secured the planar structure of **3**, of which the double bond at C-10 was *trans* in view of the large coupling constant (*J* = 15.2) between H-10 and H-11. Moreover, H-3 and H-6 were located on the opposite face of the tetrahydrofuran ring, as supported by the NOESY correlation between H-3 and H-7. The relationships of H-2/H-3 and H-6/H-7 were deduced to be *erythro* and *threo*, respectively, by comparison of NMR data with those for annonacin A [21]. The systematic name of compound **3** was (*E*)-1-(5-(1-hydroxyethyl)tetrahydrofuran-2-yl)hepta-4,6-dien-1-ol.

Tricholixin D (**4**) was assigned a molecular formula of C_14_H_24_O_4_ by interpretation of HRESI(+)MS data. Two propenyl groups were deduced in the molecule by comparing NMR data (Table 3 and Table 4) with literature ones [22], and they were also confirmed by the HMBC correlations from H_3_-1 to C-2 and C-3 and from H_3_-13 to C-11 and C-12. One propenyl group was extended to the nonprotonated C-8 via an ethylene group according to the COSY correlations from H-9b to H_2_-10 and the HMBC correlations from H-9b to C-8 and from H_2_-10 to C-11 and C-12. The other was bonded to a hydroxylated methine group based on the HMBC correlations from H-4 to C-2 and C-3 and then elongated to C-8 based on the COSY correlations from OH-4 through to HO-7 and the HMBC correlations from OH-7 to C-6, C-7, and C-8. The remaining NMR signals corresponded to a methoxy group [23], which was attached to C-8 on the basis of their HMBC correlation. To satisfy the molecular formula, an ether linkage was situated between C-5 and C-8. Taken together, the planar structure of **4** was completed. As indicated by the signal of H-2, the large coupling constant (*J* = 15.4) between H-2 and H-3 suggested the double bond at C-2 to be *trans*. Although the coupling constants of the other two olefinic methines (C-11 and C-12) failed to be diagnosed, their chemical shifts demonstrated the double bond at C-11 to be *trans* [22]. Moreover, H-5, OH-7, and CH_3_O-8 were oriented on the same face of the tetrahydrofuran ring by the NOESY correlations between H-5 and CH_3_O-8 and between H-7 and H-9b. The relationship between H-4 and H-5 was speculated to be *threo* according to the similar coupling constants of H-5 with that of (6*S*,7*R*,9*R*,10*R*)-6,9-epoxynonadec-18-ene-7,10-diol [24]. Despite the wide distribution of the tetrahydrofuran motif in marine natural products, ketal-bearing ones have rarely been discovered so far [25]. Therefore, compound **4** was named 5-((*E*)-1-hydroxybut-2-en-1-yl)-2-methoxy-2-((*E*)-pent-3-en-1-yl)tetrahydrofuran-3-ol.

**Figure 3:**
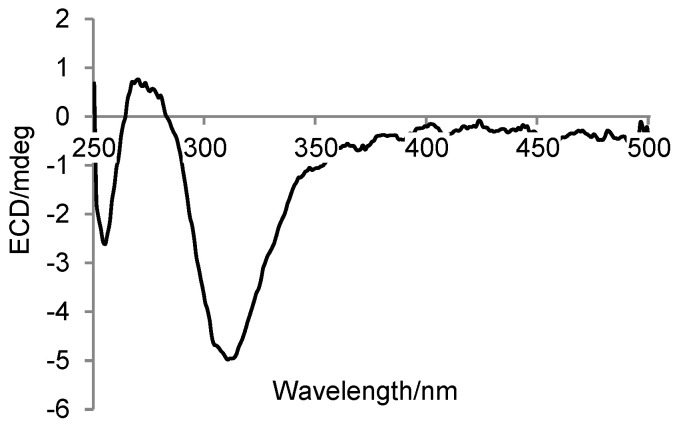
Induced ECD spectrum of Mo_2_-complex of **5** in DMSO.

Tricholixin E (**5**) possesses a molecular formula of C_13_H_20_O_3_, established by HRESI(−)MS data. Its IR spectrum exhibited absorption bands for hydroxy and carbonyl groups at 3439 and 1735 cm^−1^. The doublet at δ_H_ 1.64 (H_3_-13) in the ^1^H NMR spectrum (Table 3) and the low-frequency signal at δ_C_ 18.1 in the ^13^C NMR spectrum (Table 4) were attributed to a methyl group by analysis of HSQC and DEPT data. Its linkage with a double bond was deduced by the HMBC correlations from H_3_-13 to C-11 and C-12 and further verified by comparison of NMR data with those for **4**. The high singlet at δ_H_ 2.28 (H_3_-1) in the ^1^H NMR spectrum was also ascribed to a methyl group, which was then connected to a carbonyl group to form an acetyl unit based on the HMBC correlation from H_3_-1 to C-2. In addition, a large spin system from C-3 to C-10 was established by the COSY correlations as shown in Figure 2 and was further validated by the identical NMR data with those for the same unit reported in the literature [26]. These three moieties were linked together according to the HMBC correlation from H_2_-10 to C-11 and C-12, from H-9b to C-11, and from H_3_-1 to C-3. To ascertain the absolute configurations at C-7 and C-8, a dimolybdenum-induced ECD spectrum (Figure 3) was determined by the addition of Mo_2_(OAc)_4_. The curve with a negative Cotton effect at 310 nm matched well with that of neocyclocitrinol A, which features a *threo* vicinal diol unit with *R*,*R* configurations according to the empirical helicity rule [27]. Thus, compound **5** was named (3*E*,5*E*,7*R*,8*R*,11*E*)-7,8-dihydroxytrideca-3,5,11-trien-2-one.

The two known compounds, brasilane A (**6**) and harzianone A (**7**), were identified by comparison of their spectroscopic data with those reported in the literature [28,29,30]. Compound **6** features a brasilane skeleton that has been rarely discovered from *Trichoderma* [31,32]. It is the first brasilane aminoglycoside from *Trichoderma*. Compound **7** possesses a harziane scaffold, and its discovery further verifies the universality of this class of diterpenoids in *Trichoderma* [33].

### 2.2. Antifungal Activity of Isolated Compounds

Compounds **1**–**7** were evaluated for their inhibition of two wheat-pathogenic fungi, *Fusarium graminearum* ACCC39334 and *Gaeumannomyces graminis* ACCC38864. The results showed only compounds **1**, **2**, and **6** were active against one of the two pathogens (Table 5). Compounds **1** and **2** inhibited *F. graminearum* ACCC39334 with an MIC value of 25.0 μg/mL, and **6** suppressed *G. graminis* ACCC38864 with an MIC value of 12.5 μg/mL. Although harzianolide and its 2-oxo derivative effectively inhibited *G. graminis* at 200 and 100 μg/plug [34], no activities were detected at a concentration of 50 μg/mL for **1** and **2**. This might result from the low concentration of the two new compounds or the structural discrepancy of these butenolides. The inhibition effect of **1** and **2** on *F. graminearum* further demonstrated the potency of butenolides to antagonize plant-pathogenic fungi. Additionally, terpenoid aminoglycosides are a family of metabolites that have often been discovered from marine-derived *Trichoderma* in recent years [10,35,36]. The high inhibition effect of **6** on *G. graminis* may afford a new target for the application of this class of molecules. On the other hand, the brine shrimp lethality of **1**–**7** was also evaluated, and only <24% inhibition rates were detected for these compounds at 100 μg/mL. The brine shrimp *Artemia salina* was widely used to predict human toxicity to environmental chemicals and natural products [37]. The low toxicity of compounds **1**, **2**, and **6** may suggest their prospect in the development of antifungal agents in agriculture.

## 3. Materials and Methods

### 3.1. General Experimental Producres

Similar to previous procedures [10,15,16,17,23], Autopol VI polarimeters were applied to determine optical rotations. The Chirascan CD spectrometer was applied to measure UV and ECD data. The Nicolet iS50 FT-IR spectrometer was applied to acquire IR data. The Bruker Avance III 500 NMR spectrometer (500 MHz for ^1^H and 125 MHz for ^13^C) was applied to record 1D/2D NMR data. The Xevo G2-XS QTof mass spectrometer was applied to obtain HRESIMS data. The Agilent 1260 Infinity II system with a ZORBAX SB-C18 (5 μm, 9.4 × 250 mm) column was applied for HPLC separation. Silica gel (200–300 mesh, Qingdao Haiyang Chemical Co., Qingdao, China), RP-18 (AAG12S50, YMC Co., Ltd., Kyoto, Japan), and Sephadex LH-20 (GE Healthcare, Chicago, IL, USA) were employed for column chromatography (CC). Precoated silica gel plates (GF-254, 20 × 20 cm, Qingdao Haiyang Chemical Co., Qingdao, China) were used for thin-layer chromatography (TLC).

### 3.2. Fungal Material and Fermentation

*Trichoderma lixii* R22 was obtained from the sediments collected from a deep-sea (−1217 m) cold seep off southwestern Taiwan Island in May 2020. Its identification was fulfilled by analysis of the internal transcribed spacer rDNA sequence data (GenBank no. OQ745814, Bethesda, MD, USA). The static cultivation was completed in 150 × 1 L Erlenmeyer flasks at 25 °C for 40 days. Each flask mainly harbored 25.0 g of rice, 1.0 g of glucose, 0.25 g of peptone, 0.25 g of yeast extract, 0.25 g of monosodium glutamate, 0.05 g of NaBr, 25.0 mL of pure water, and 25.0 mL of natural seawater (Yantai coast).

### 3.3. Extraction and Isolation

Some 50 mL EtOAc was poured into each flask to end the cultivation. Mycelia were collected by filtration, which were further dried and then extracted with CH_2_Cl_2_/MeOH (1:1, *v*/*v*) three times. After evaporating organic solvents under negative pressure, the mycelial extract (308 g) was obtained. The filtrate was extracted with EtOAc and then concentrated to afford the broth extract (38 g). The two parts (346 g) were combined and then subjected to silica gel CC with step-gradient solvent systems comprising petroleum ether (PE)/EtOAc and CH_2_Cl_2_/MeOH to give 9 fractions (Frs. 1–9). Fr. 5 eluted with PE/EtOAc (2:1) and was further separated by RP-18 CC (MeOH/H_2_O, 17:3), preparative TLC (PE/EtOAc, 2:1), and Sephadex LH-20 CC (MeOH) to provide **7** (12.0 mg). Fr. 7 eluted with EtOAc and was further purified by RP-18 CC (MeOH/H_2_O, 2:3 to 1:1) and Sephadex LH-20 CC (MeOH) and semipreparative HPLC (MeOH/H_2_O, 7/13 to 2/3 for 20 min, RT = 10 min; ACN/H_2_O, 3/22 to 17/83 for 20 min, RT = 8 min; MeOH/H_2_O, 1/1 to 11/9 for 20 min, RT = 12 min and 10 min, respectively, 3.0 mL/min, UV detection at 210 nm) to obtain **1** (6.6 mg), **2** (3.1 mg), **4** (1.7 mg), and **5** (3.0 mg). Fr. 7 eluted with EtOAc and was further purified by RP-18 CC (MeOH/H_2_O, 3:2) and preparative TLC (CH_2_Cl_2_/MeOH, 15:1) and Sephadex LH-20 CC (MeOH) to acquire **3** (2.1 mg). Fr. 10 eluted with CH_2_Cl_2_/MeOH (10:1) and was further separated by RP-18 CC (MeOH/H_2_O, 4:1) and Sephadex LH-20 CC (MeOH) as well as semipreparative HPLC (ACN/H_2_O, 7/3 to 4/1 for 20 min, 3.0 mL/min, RT = 8 min, UV detection at 210 nm) to afford **6** (15.0 mg).

### 3.4. Spectral and Physical Data of Compounds ***1**–**5***

Tricholixin A (**1**): colorless oil; ${\left[\alpha \right]}_{D}^{20}$ +1.1 (*c* 0.13, MeOH); UV (MeOH) λ_max_ (log ε) 264 (4.13) nm; IR (KBr) *v*_max_ 3440, 2924, 2853, 1738, 1647, 1452, 1383, 1031 cm^−1^; ^1^H and ^13^C NMR data, Table 1 and Table 2; HRESI(−)MS *m*/*z* 237.1123 [M − H]^−^ (calcd for C_13_H_17_O_4_, 237.1127).

Tricholixin B (**2**): colorless oil; ${\left[\alpha \right]}_{D}^{20}$ + 8.0 (*c* 0.10, MeOH); UV (MeOH) λ_max_ (log *ε*) 216 (3.87), 260 (3.42) nm; IR (KBr) *v*_max_ 3430, 2923, 2854, 1740, 1635, 1451, 1399, 1033 cm^−1^; ^1^H and ^13^C NMR data, Table 1 and Table 2; HRESI(+)MS *m*/*z* 235.0945 [M + Na]^+^ (calcd for C_11_H_16_O_4_Na, 235.0946).

Tricholixin C (**3**): colorless oil; ${\left[\alpha \right]}_{D}^{20}$ − 6.2 (*c* 0.10, MeOH); UV (MeOH) λ_max_ (log *ε*) 225 (4.27) nm; IR (KBr) *v*_max_ 3425, 2924, 2855, 1631, 1450, 1385, 1057 cm^−1^; ^1^H and ^13^C NMR data, Table 3 and Table 4; HRESI(+)MS *m*/*z* 249.1479 [M + Na]^+^ (calcd for C_13_H_22_O_3_Na, 249.1467).

Tricholixin D (**4**): colorless oil; ${\left[\alpha \right]}_{D}^{20}$ − 31.5 (*c* 0.12, MeOH); IR (KBr) *v*_max_ 3402, 2925, 1642, 1549, 1405, 1006 cm^−1^; ^1^H and ^13^C NMR data, Table 3 and Table 4; HRESI(+)MS *m*/*z* 279.1561 [M + Na]^+^ (calcd for C_14_H_24_O_4_Na, 279.1572).

Tricholixin E (**5**): colorless oil; ${\left[\alpha \right]}_{D}^{20}$ − 26.0 (*c* 0.090, MeOH); UV (MeOH) λ_max_ (log *ε*) 271 (4.52) nm; IR (KBr) *v*_max_ 3439, 2923, 2856, 2356, 1735, 1635, 1450, 1383, 1099, 1047, 969 cm^−1^; ^1^H and ^13^C NMR data, Table 3 and Table 4; HRESI(−)MS *m*/*z* 223.1326 [M − H]^−^ (calcd for C_13_H_19_O_3_, 223.1334).

### 3.5. ECD Determination for Mo_2_-Complex of ***5***

Solutions of **5** and dimolybdenum tetraacetate, Mo_2_(OAc)_4_, were prepared by dissolving appropriate amounts of them in 1.0 mL of DMSO solvent of analytical grade, respectively, and both the concentrations were 1.5 mg/mL. Subsequently, 0.15 mL of the two solutions were mixed and then poured into a quartz cuvette with a 1.0 mm optical path length. In the mixture, the molar ratio of **5** to Mo_2_(OAc)_4_ was 1.9:1. Within the first 40 min, ECD signals were continually determined until the emergence of an invariant spectrum.

### 3.6. Assay for Antifungal Activity

Antifungal assay toward the wheat-pathogenic *Fusarium graminearum* ACCC39334 and *Gaeumannomyces graminis* ACCC38864, which were purchased from the Agricultural Culture Collection of China, was carried out through the microdilution method in a 96-well plate as described previously [10]. During the antifungal test, carbendazim was taken as the positive control. Following a previous procedure [38], the brine shrimp lethality was assayed against *Artemia salina*, with CuSO_4_ being a positive control.

## 4. Conclusions

Chemical investigation of the deep-sea fungus *Trichoderma lixii* R22 resulted in the isolation and identification of five new lipids, tricholixins A–E (**1**–**5**), and two known terpenoids, brasilane A (**6**) and harzianone A (**7**), and compounds **1** and **2** represent the only two new butenolides from marine-derived *Trichoderma*. In addition, compound **6** is the first brasilane aminoglycoside of *Trichoderma* origin. Compounds **1**–**7** were evaluated for inhibition against two plant-pathogenic fungi and one marine plankton species. Among them, compounds **1** and **2** inhibited *F. graminearum* with an MIC value of 25.0 μg/mL, and the structure–activity relationship suggested that butenolides could enhance the antifungal activity of these lipids. On the other hand, the high inhibition effect of **6** on *G. graminis* may afford a new target for the application of this class of molecules. The low toxicity of compounds **1**, **2**, and **6** may suggest their prospect in the development of antifungal agents in agriculture. An in-depth study including chemical modification and biosynthesis of these isolates will be conducted to improve their bioactivity in our further research.

## Figures and Tables

**Table 1:** ^1^H NMR Data for **1** and **2** (δ in ppm, *J* in Hz).

Position	1 (in DMSO-*d*_6_)	1 (in CDCl_3_)	2 (in DMSO-*d*_6_)	2 (in CD_3_OD)
1	1.02, d (6.2)	1.23, d (6.2)	1.03, d (6.2)	1.18, d (6.2)
2	3.77, m	4.05, m	3.77, m	3.97, m
3a	2.36, m	2.53, m	2.30, dd (13.4, 7.1)	2.43, br dd (13.8, 7.6)
3b	2.26, br dd (13.3, 5.8)	2.46, m	2.21, dd (13.4, 5.8)	2.37, br dd (13.8, 5.2)
6a	6.54, br d (16.1)	6.47, br d (16.1)	3.21, dd (16.4, 6.8)	3.28, br d (6.7)
6b			3.18, dd (16.4, 7.2)	
7	6.14, dt (16.1, 6.7)	6.05, dt (16.1, 6.9)	5.60, br ddd (15.3, 7.2, 6.8)	5.71, dtt (15.3, 6.7, 1.1)
8	2.38, m	2.49, m	5.69, br dd (15.3, 5.0)	5.79, br dt (15.3, 5.2)
9	2.62, t (7.3)	2.62, t (7.0)	3.89, br s	4.04, br dd (5.2, 1.1)
11	2.10, s	2.17, s		
12a	4.95, d (16.5)	4.89, s	4.75, d (17.9)	4.78, s
12b	4.91, d (16.5)		4.69, d (17.9)	
OH-2	4.63, br d (4.1)		4.62, br d (4.3)	
OH-9			4.68, m	

**Table 2:** ^13^C NMR Data for **1** and **2** (δ in ppm).

Position	1 (in DMSO-*d*_6_)	1 (in CDCl_3_)	2 (in DMSO-*d*_6_)	2 (in CD_3_OD)
1	23.2, CH_3_	23.5, CH_3_	23.3, CH_3_	23.3, CH_3_
2	64.8, CH	66.7, CH	64.6, CH	67.0, CH
3	33.2, CH_2_	33.5, CH_2_	33.1, CH_2_	34.1, CH_2_
4	122.0, C	123.4, C	123.1, C	125.0, C
5	156.0, C	155.7, C	162.0, C	164.1, C
6	121.1, CH	121.5, CH	29.6, CH_2_	31.0, CH_2_
7	138.6, CH	138.3, CH	124.0, CH	126.5, CH
8	26.8, CH_2_	27.3, CH_2_	133.8, CH	134.4, CH
9	41.2, CH_2_	42.2, CH_2_	61.0, CH_2_	63.0, CH_2_
10	207.4, C	207.1, C		
11	29.8, CH_3_	30.1, CH_3_		
12	69.3, CH_2_	69.9, CH_2_	71.2, CH_2_	73.1, CH_2_
13	174.8, C	176.0, C	174.7, C	177.7, C

**Table 3:** ^1^H NMR Data for **3**–**5** (δ in ppm, *J* in Hz).

Position	3 (in CD_3_OD)	4 (in DMSO-*d*_6_)	5 (in CD_3_OD)
1	1.13, d (6.4)	1.64, br d (6.5)	2.28, s
2	3.71, qd (6.4, 5.0)	5.61, dqd (15.4, 6.5, 1.2)	
3	3.79, m	5.40, m	6.16, d (15.7)
4a	1.95, m	3.82, m	7.30, dd (15.7, 10.7)
4b	1.82, m		
5a	1.95, m	3.75, ddd (9.0, 4.4, 3.4)	6.48, br dd (15.3, 10.7)
5b	1.82, m		
6a	3.82, m	1.97, ddd (11.9, 8.5, 3.4)	6.37, dd (15.3, 5.8)
6b		1.72, m	
7	3.53, ddd (8.9, 5.3, 3.2)	3.85, q (8.5)	4.05, ddd (5.8, 5.3, 0.9)
8a	1.64, m		3.51, ddd (9.4, 5.3, 3.1)
8b	1.42, m		
9a	2.30, m	1.71, m	1.61, m
9b	2.15, m	1.58, m	1.43, m
10a	5.72, dt (15.2, 7.0)	2.09, m	2.19, m
10b		1.91, m	2.03, m
11	6.09, dd (15.2, 10.3)	5.40, m	5.45, m
12	6.31, dt (17.0, 10.3)	5.40, m	5.45, m
13a	5.06, dd (17.0, 2.0)	1.60, br d (4.9)	1.64, br d (4.7)
13b	4.93, dd (10.3, 2.0)		
CH_3_O-8		3.14, s	
OH-4		4.75, br d (4.7)	
OH-7		4.43, br d (8.5)	

**Table 4:** ^13^C NMR Data for **3**–**5** (δ in ppm).

Position	3 (in CD_3_OD)	4 (in DMSO-*d*_6_)	5 (in CD_3_OD)
1	19.5, CH_3_	17.7, CH_3_	27.0, CH_3_
2	70.2, CH	125.4, CH	201.6, C
3	85.0, CH	131.8, CH	131.1, CH
4	27.9, CH_2_	72.6, CH	145.5, CH
5	27.8, CH_2_	78.3, CH	130.5, CH
6	84.1, CH	32.6, CH_2_	145.3, CH
7	73.5, CH	73.6, CH	76.2, CH
8	34.2, CH_2_	104.5, C	74.9, CH
9	29.8, CH_2_	32.1, CH_2_	33.8, CH_2_
10	135.7, CH	26.4, CH_2_	29.8, CH_2_
11	132.7, CH	131.5, CH	132.1, CH
12	138.6, CH	124.0, CH	126.2, CH
13	115.1, CH_2_	17.8, CH_3_	18.1, CH_3_
CH_3_O-8		47.4, CH_3_	

**Table 5:** Inhibition of two wheat-pathogenic fungi by **1**–**7**.

Compound	MIC (μg/mL)		Lethal Rate (at 100 μg/mL)
	*Fusarium graminearum* ACCC39334	*Gaeumannomyces graminis* ACCC38864	*Artemia salina*
**1**	25.0 ± 0.0	–	15.6 ± 5.2%
**2**	25.0 ± 0.0	–	15.2 ± 2.1%
**3**	–	–	4.7 ± 1.3%
**4**	–	–	19.9 ± 3.7%
**5**	–	–	7.2 ± 5.3%
**6**	–	12.5 ± 0.0	23.4 ± 5.4%
**7**	–	–	0.0 ± 0.0%
carbendazim	6.1 ± 0.0	6.1 ± 0.0	
CuSO_4_			100.0 ± 0.0%

– no inhibition effect at 50 μg/mL.

## Data Availability

Data of the compounds are available in Supplementary Materials.

## Supplementary Materials

The following supporting information can be downloaded at: https://www.mdpi.com/article/10.3390/molecules28176220/s1, Figures S1–S61: 1D/2D NMR, HRESIMS, IR, and UV spectra of **1**–**5** and ^1^H and ^13^C NMR as well as DEPT spectra of **6** and **7**.

## Sample Availability

Not all samples of the compounds (**1**–**7**) are available from the authors since some compounds had been used up after their bioassay.

## References

[1] Shao H.B., Liang Z.S., Shao M.A., Wang B.C. (2005). Changes of anti-oxidative enzymes and membrane peroxidation for soil water deficits among 10 wheat genotypes at seedling stage. Colloid Surf. B Biointerfaces.

[2] Figueroa M., Hammond-Kosack K.E., Solomon P.S. (2018). A review of wheat diseases-a field perspective. Mol. Plant Pathol..

[3] Goswami R.S., Kistler H.C. (2004). Heading for disaster: *Fusarium graminearum* on cereal crops. Mol. Plant Pathol..

[4] Freeman J., Ward E. (2004). *Gaeumannomyces graminis*, the take-all fungus and its relatives. Mol. Plant Pathol..

[5] Cheng Y.-N., Sun L., Meng H., Jiang Z., Zhang Z., Yun Y., Wang X., Yan J., Yang X., Zhou H. (2022). Structure-activity studies of N-heterocyclic benzoyl arylamine derivatives led to a highly fungicidal candidate against *Gaeumannomyces graminis* var. *tritici* and four *Fusarium* wheat pathogens. J. Agric. Food Chem..

[6] Zhang X., Chen X., Qiao X., Fan X., Huo X., Zhang D. (2021). Isolation and yield optimization of lipopeptides from *Bacillus subtilis* Z-14 active against wheat take-all caused by *Gaeumannomyces graminis* var. tritici. J. Sep. Sci..

[7] Woo S.L., Ruocco M., Vinale F., Nigro M., Marra R., Lombardi N., Pascale A., Lanzuise S., Manganiello G., Lorito M. (2014). *Trichoderma*-based products and their widespread use in agriculture. Open Mycol. J..

[8] Ghisalberti E.L., Sivasithamparam K. (1991). Antifungal antibiotics produced by *Trichoderma* spp.. Soil Biol. Biochem..

[9] Reino J.L., Guerrero R.F., Hernández-Galán R., Collado I.G. (2008). Secondary metabolites from species of the biocontrol agent *Trichoderma*. Phytochem. Rev..

[10] Shi Z.-Z., Liu X.-H., Li X.-N., Ji N.-Y. (2020). Antifungal and antimicroalgal trichothecene sesquiterpenes from the marine algicolous fungus *Trichoderma brevicompactum* A-DL-9-2. J. Agric. Food Chem..

[11] You J., Dai H., Chen Z., Liu G., He Z., Song F., Yang X., Fu H., Zhang L., Chen X. (2010). Trichoderone, a novel cytotoxic cyclopentenone and cholesta-7, 22-diene-3β, 5α, 6β-triol, with new activities from the marine-derived fungus *Trichoderma* sp.. J. Ind. Microbiol. Biotechnol..

[12] Li H., Liu X., Li X., Hu Z., Wang L. (2021). Novel harziane diterpenes from deep-sea sediment fungus *Trichoderma* sp. SCSIOW21 and their potential anti-inflammatory effects. Mar. Drugs.

[13] Hao M.-J., Chen P.-N., Li H.-J., Wu F., Zhang G.-Y., Shao Z.-Z., Liu X.-P., Ma W.-Z., Xu J., Mahmud T. (2022). β-Carboline alkaloids from the deep-sea fungus *Trichoderma* sp. MCCC 3A01244 as a new type of anti-pulmonary fibrosis agent that inhibits TGF-b/Smad signaling pathway. Front. Microbiol..

[14] Li H., Liu X., Hu Z., Wang L. (2023). Novel sesquiterpene and diterpene aminoglycosides from the deep-sea-sediment fungus *Trichoderma* sp. SCSIOW21. Mar. Drugs.

[15] Liu Y.-P., Fang S.-T., Shi Z.-Z., Wang B.-G., Li X.-N., Ji N.-Y. (2021). Phenylhydrazone and quinazoline derivatives from the cold-seep-derived fungus *Penicillium oxalicum*. Mar. Drugs.

[16] Li C.-P., Song Y.-P., Wang B.-G., Ji N.-Y. (2022). Sulfurated and iodinated metabolites from the cold-seep fungus *Cladosporium cladosporioides* 8-1. Tetrahedron Lett..

[17] Liu Y.-P., Fang S.-T., Wang B.-G., Ji N.-Y. (2022). Phenol derivatives from the cold-seep fungus *Aspergillus sydowii* 10-31. Phytochem. Lett..

[18] Almassi F., Ghisalberti E.L., Narvey M.J., Sivasithamparam K. (1991). New antibiotics from strains of *Trichoderma harzianum*. J. Nat. Prod..

[19] Claydon N., Hanson J.R., Truneh A., Avent A.G. (1991). Harzianolide, a butenolide metabolite from cultures of *Trichoderma harzianum*. Phytochemistry.

[20] Fujimoto Y., Murasaki C., Shimada H., Nishioka S., Kakinuma K., Singh S., Singh M., Gupta Y.K., Sahai M. (1994). Annonaceous acetogenins from the seeds of *Annona squamosa*. Non-adjacent bis-tetrahydrofuranic acetogenins. Chem. Pharm. Bull..

[21] Harmange J.-C., Figadère B., Cavé A. (1992). Stereocontrolled synthesis of 2,5-linked monotetrahydrofuran units of acetogenins. Tetrahedron Lett..

[22] Liu X.-H., Ji N.-Y. (2022). Isolation, identification, and bioactivity of a new triol from algicolous fungus *Trichoderma citrinoviride*. Chem. Bioeng..

[23] Liu X.-H., Song Y.-P., Yin X.-L., Ji N.-Y. (2022). Antimicrobial terpenoids and polyketides from the algicolous fungus *Byssochlamys spectabilis* RR-dl-2-13. J. Agric. Food Chem..

[24] Capon R.J., Barrow R.A., Rochfort S., Jobling M., Skene C., Lacey E., Gill J.H., Friedel T., Wadsworth D. (1998). Marine nematocides: Tetrahydrofurans from a southern Australian brown alga, *Notheia anomala*. Tetrahedron.

[25] González-Andrés P., Fernández-Peña L., Díez-Poza C., Barbero A. (2022). The tetrahydrofuran motif in marine lipids and terpenes. Mar. Drugs.

[26] Miyata Y., Matsunaga S. (2008). Structure elucidation of 21,22-dihydroxyonnamides A_1_-A_4_ from the marine sponge *Theonella swinhoei*: An empirical rule to assign the relative stereochemistry of linear 1,5-diols. Tetrahedron Lett..

[27] Xia M.-W., Cui C.-B., Li C.-W., Wu C.-J. (2014). Three new and eleven known unusual C25 steroids: Activated production of silent metabolites in a marine-derived fungus by chemical mutagenesis strategy using diethyl sulphate. Mar. Drugs.

[28] Feng J., Surup F., Hauser M., Miller A., Wennrich J.-P., Stadler M., Cox R.J., Kuhnert E. (2020). Biosynthesis of oxygenated brasilane terpene glycosides involves a promiscuous *N*-acetylglucosamine transferase. Chem. Commun..

[29] Hu D.-B., Zhang S., He J.-B., Dong Z.-J., Li Z.-H., Feng T., Liu J.-K. (2015). Brasilane sesquiterpenoids and alkane derivatives from cultures of the basidiomycete *Coltricia sideroides*. Fitoterapia.

[30] Zhao D.-L., Yang L.-J., Shi T., Wang C.-Y., Shao C.-L., Wang C.-Y. (2019). Potent phytotoxic harziane diterpenes from a soft coral-derived strain of the fungus *Trichoderma harzianum* XS-20090075. Sci. Rep..

[31] Murai K., Lauterbach L., Teramoto K., Quan Z., Barra L., Yamamoto T., Nonaka K., Shiomi K., Nishiyama M., Kuzuyama T. (2019). An unusual skeletal rearrangement in the biosynthesis of the sesquiterpene trichobrasilenol from *Trichoderma*. Angew. Chem. Int. Ed..

[32] Sun X., Cai Y.-S., Yuan Y., Bian G., Ye Z., Deng Z., Liu T. (2019). Genome mining in *Trichoderma viride* J1-030: Discovery and identification of novel sesquiterpene synthase and its products. Beilstein J. Org. Chem..

[33] Zou J.-X., Song Y.-P., Zeng Z.-Q., Ji N.-Y. (2021). Proharziane and harziane derivatives from the marine algicolous fungus *Trichoderma asperelloides* RR-dl-6-11. J. Nat. Prod..

[34] Vinale F., Marra R., Scala F., Ghisalberti E.L., Lorito M., Sivasithamparam K. (2006). Major secondary metabolites produced by two commercial *Trichoderma* strains active against different phytopathogens. Lett. Appl. Microbiol..

[35] Song Y.-P., Liu X.-H., Shi Z.-Z., Miao F.-P., Fang S.-T., Ji N.-Y. (2018). Bisabolane, cyclonerane, and harziane derivatives from the marine-alga-endophytic fungus *Trichoderma asperellum* cf44-2. Phytochemistry.

[36] Song Y.-P., Miao F.-P., Liu X.-H., Yin X.-L., Ji N.-Y. (2019). Seven chromanoid norbisabolane derivatives from the marine-alga-endophytic fungus *Trichoderma asperellum* A-YMD-9-2. Fitoterapia.

[37] Hamidi M.R., Jovanova B., Panovska T.K. (2014). Toxicological evaluation of the plant products using brine shrimp (*Artemia salina* L.) model. Maced. Pharm. Bull..

[38] Miao F.P., Liang X.R., Yin X.L., Wang G., Ji N.Y. (2012). Absolute configurations of unique harziane diterpenes from *Trichoderma* species. Org. Lett..

