# Supplementary material for: Lipids and Terpenoids from the Deep-Sea Fungus Trichoderma lixii R22 and Their Antagonism against Two Wheat Pathogens
Source: Molecules. 2023 Aug 24;28(17):6220. doi: 10.3390/molecules28176220 (PMC10488430; doi:10.3390/molecules28176220)
Supplement: Supplementary file 1 [file molecules-28-06220-s001.zip › Supplementary Information.pdf]

Supplementary Materials

Lipids and Terpenoids from the Deep-Sea Fungus *Trichoderma lixii* R22 and Their Antagonism against Two Wheat Pathogens

Chang-Peng Li[1,2,†] , Zhen-Zhen Shi[1,†], Sheng-Tao Fang[1], Yin-Ping Song[1], and Nai-Yun Ji[1,2,3,*]

[1] Yantai Institute of Coastal Zone Research, Chinese Academy of Sciences, Yantai 264003, China; cpli@yic.ac.cn (C.-P.L.); zzshi@yic.ac.cn (Z.-Z.S.); stfang@yic.ac.cn (S.-T.F.); ypsong@yic.ac.cn (Y.-P.S.)
[2] University of Chinese Academy of Sciences, Beijing 100049, China
[3] Shandong Saline-Alkaline Land Modern Agriculture Company, Dongying 257345, China
[*] Correspondence: nyji@yic.ac.cn; Tel.: +86-535-210-9176

† These authors contributed equally to this work.

Table of Contents

Figure S1. ^{1}H NMR spectrum of compound **1** in DMSO-d_6.

Figure S2. ^{13}C NMR and DEPT spectra of compound **1** in DMSO-d_6.

Figure S3. HSQC spectrum of compound **1** in DMSO-d_6.

Figure S4. HMBC spectrum of compound **1** in DMSO-d_6.

Figure S5. COSY spectrum of compound **1** in DMSO-d_6.

Figure S6. NOESY spectrum of compound **1** in DMSO-d_6.

Figure S7. ^{1}H NMR spectrum of compound **1** in $CDCl_3$.

Figure S8. ^{13}C NMR and DEPT spectra of compound **1** in $CDCl_3$.

Figure S9. HSQC spectrum of compound **1** in $CDCl_3$.

Figure S10. HMBC spectrum of compound **1** in $CDCl_3$.

Figure S11. COSY spectrum of compound **1** in $CDCl_3$.

Figure S12. NOESY spectrum of compound **1** in $CDCl_3$.

Figure S13. HRESI(-)MS spectrum of compound **1**.

Figure S14. IR spectrum of compound **1**.

Figure S15. UV spectrum of compound **1** (0.2 mg/mL in MeOH).

Figure S16. ^{1}H NMR spectrum of compound **2** in DMSO-d_6.

Figure S17. ^{13}C NMR and DEPT spectra of compound **2** in DMSO-d_6.

Figure S18. HSQC spectrum of compound **2** in DMSO-d_6.

Figure S19. HMBC spectrum of compound **2** in DMSO-d_6.

Figure S20. COSY spectrum of compound **2** in DMSO-d_6.

Figure S21. NOESY spectrum of compound **2** in DMSO-d_6.

Figure S22. ^{1}H NMR spectrum of compound **2** in CD_3OD.

Figure S23. ^{13}C NMR and DEPT spectra of compound **2** in CD_3OD.

Figure S24. HSQC spectrum of compound **2** in CD_3OD.

Figure S25. HMBC spectrum of compound **2** in CD_3OD.

Figure S26. COSY spectrum of compound **2** in CD_3OD.

Figure S27. NOESY spectrum of compound **2** in CD_3OD.

Figure S28. HRESI(+)MS spectrum of compound **2**.

Figure S29. IR spectrum of compound **2**.

Figure S30. UV spectrum of compound **2** (0.4 mg/mL in MeOH).

Figure S31. ^{1}H NMR spectrum of compound **3** in CD_3OD.

Figure S32. ^{13}C NMR and DEPT spectra of compound **3** in CD_3OD.

Figure S33. HSQC spectrum of compound **3** in CD_3OD.

Figure S34. HMBC spectrum of compound **3** in CD_3OD.

Figure S35. COSY spectrum of compound **3** in CD_3OD.

Figure S36. NOESY spectrum of compound **3** in CD_3OD.

Figure S37. HRESI(+)MS spectrum of compound **3**.

Figure S38. IR spectrum of compound **3**.

Figure S39. UV spectrum of compound **3** (0.2 mg/mL in MeOH).

Figure S40. ^{1}H NMR spectrum of compound **4** in DMSO-d_6.

Figure S41. ^{13}C NMR and DEPT spectra of compound **4** in DMSO-d_6.

Figure S42. HSQC spectrum of compound **4** in DMSO-d_6.

Figure S43. HMBC spectrum of compound **4** in DMSO-d_6.

Figure S44. COSY spectrum of compound **4** in DMSO-d_6.

Figure S45. NOESY spectrum of compound **4** in DMSO-d_6.

Figure S46. HRESI(+)MS spectrum of compound **4**.

Figure S47. IR spectrum of compound **4**.

Figure S48. UV spectrum of compound **4** (0.4 mg/mL in MeOH).

Figure S49. ^{1}H NMR spectrum of compound **5** in CD_3OD.

Figure S50. ^{13}C NMR and DEPT spectra of compound **5** in CD_3OD.

Figure S51. HSQC spectrum of compound **5** in CD_3OD.

Figure S52. HMBC spectrum of compound **5** in CD_3OD.

Figure S53. COSY spectrum of compound **5** in CD_3OD.

Figure S54. NOESY spectrum of compound **5** in CD_3OD.

Figure S55. HRESI(-)MS spectrum of compound **5**.

Figure S56. IR spectrum of compound **5**.

Figure S57. UV spectrum of compound **5** (0.1 mg/mL in MeOH).

Figure S58. ^{1}H NMR spectrum of compound **6** in CD_3OD.

Figure S59. ^{13}C NMR and DEPT spectra of compound **6** in CD_3OD.

Figure S60. ^{1}H NMR spectrum of compound **7** in $CDCl_3$.

Figure S61. ^{13}C NMR and DEPT spectra of compound **7** in $CDCl_3$.

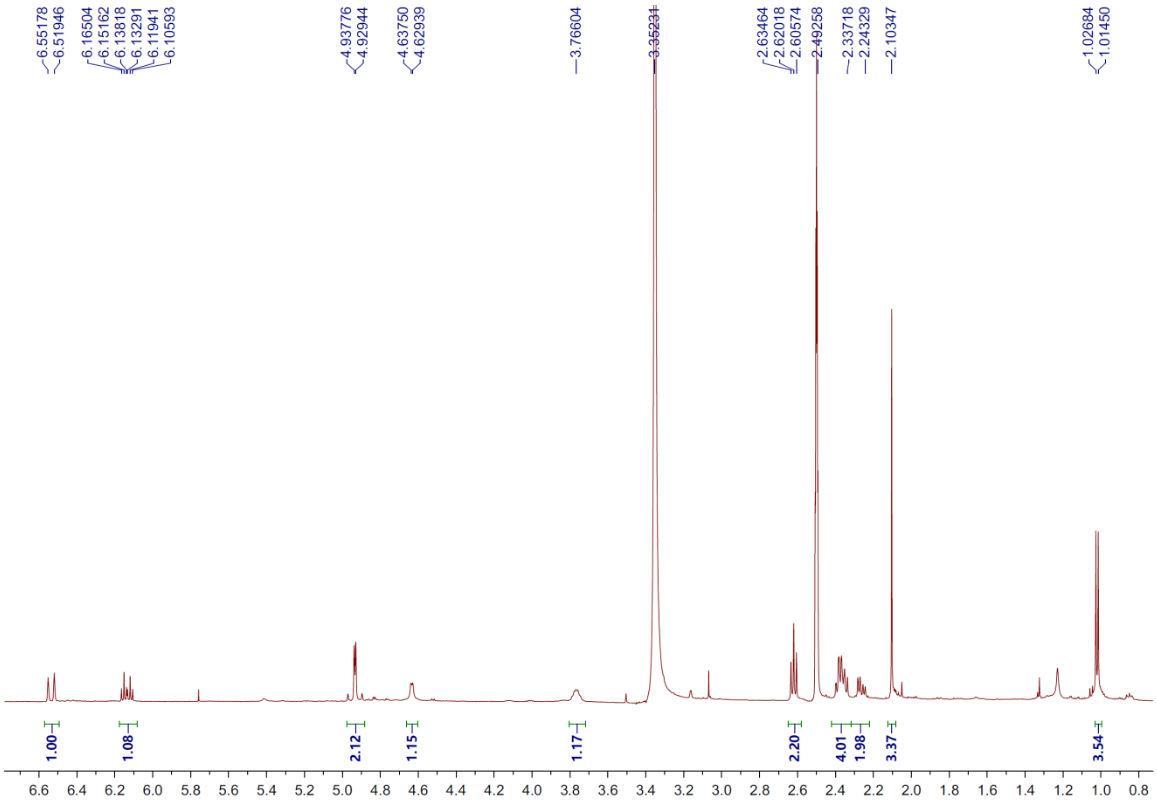

Figure S1. ^{1}H NMR spectrum of compound **1** in DMSO-d_6.

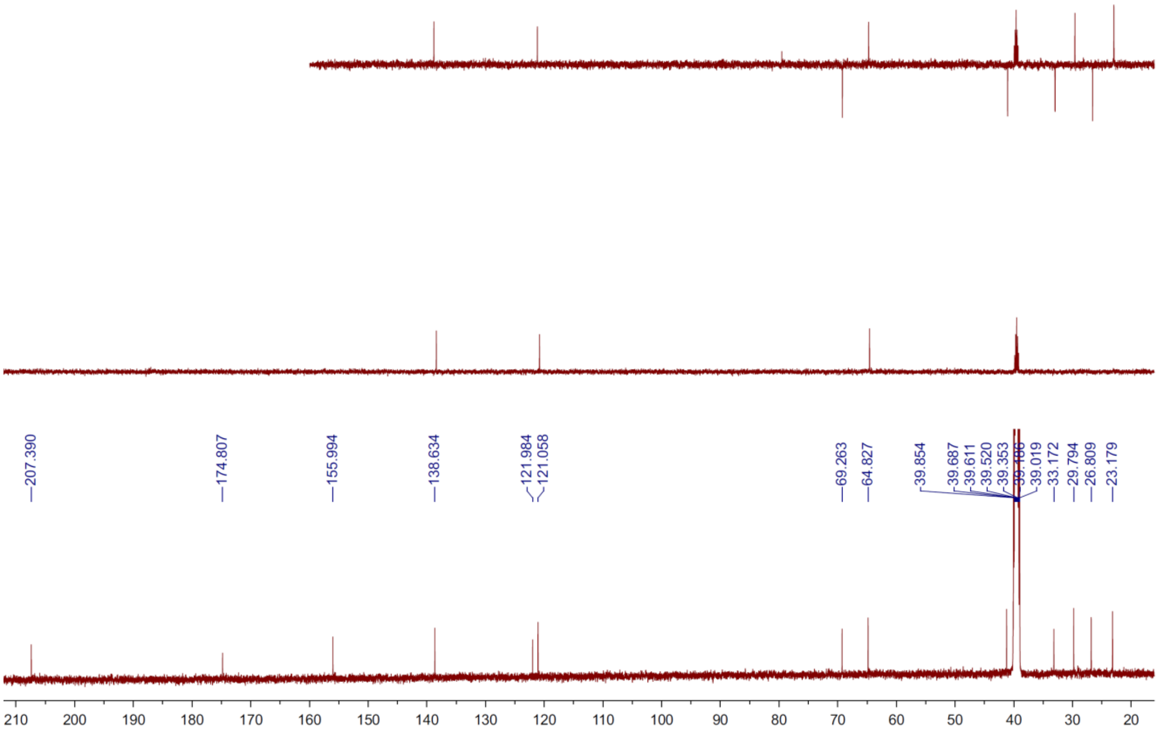

Figure S2. ^{13}C NMR and DEPT spectra of compound **1** in DMSO-d_6.

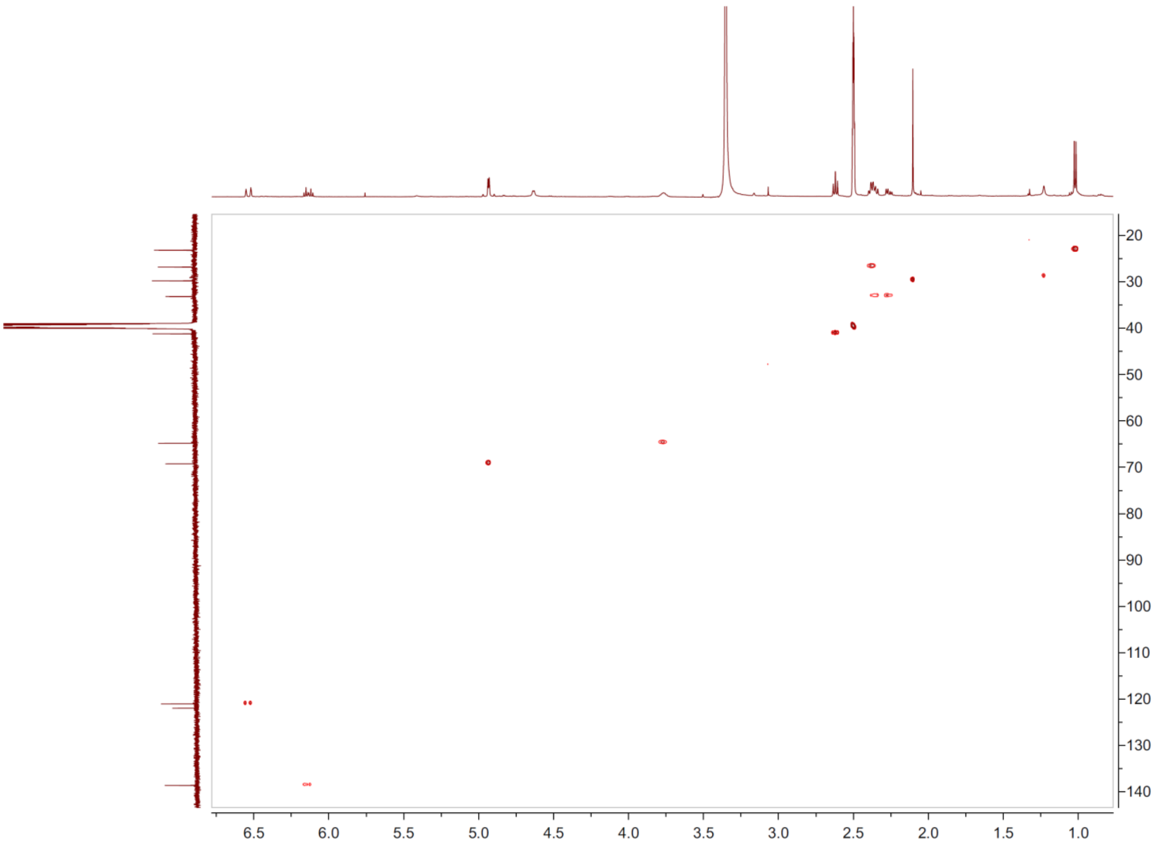

Figure S3. HSQC spectrum of compound **1** in DMSO-d_6.

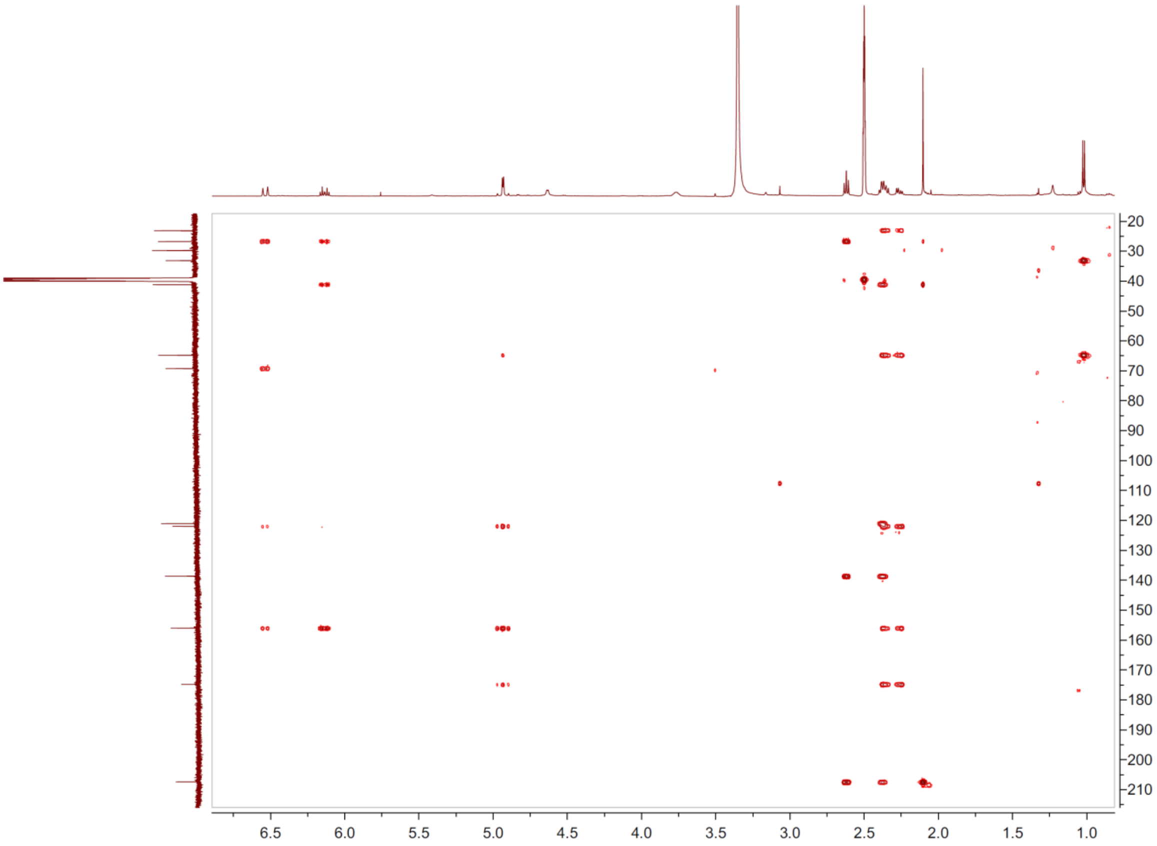

Figure S4. HMBC spectrum of compound **1** in DMSO-d_6.

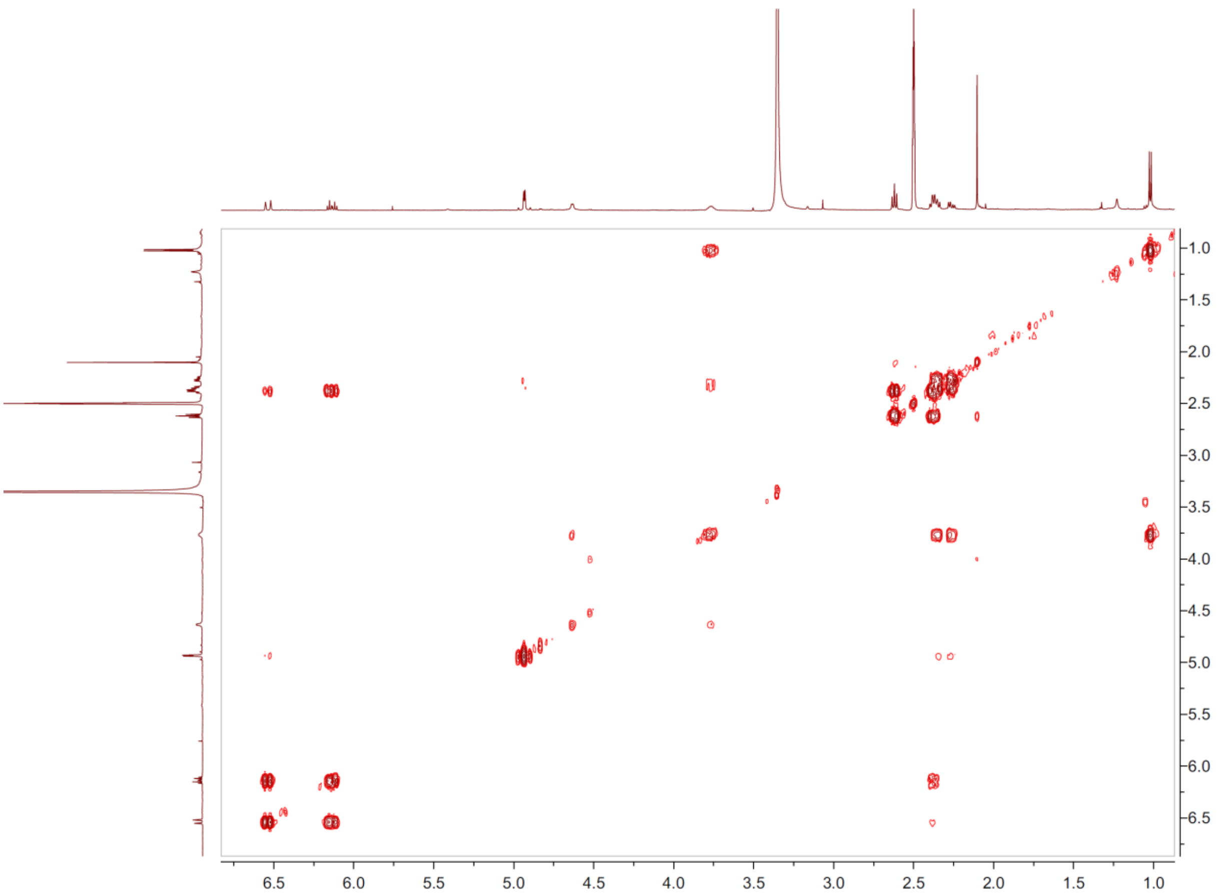

Figure S5. COSY spectrum of compound **1** in DMSO-d_6.

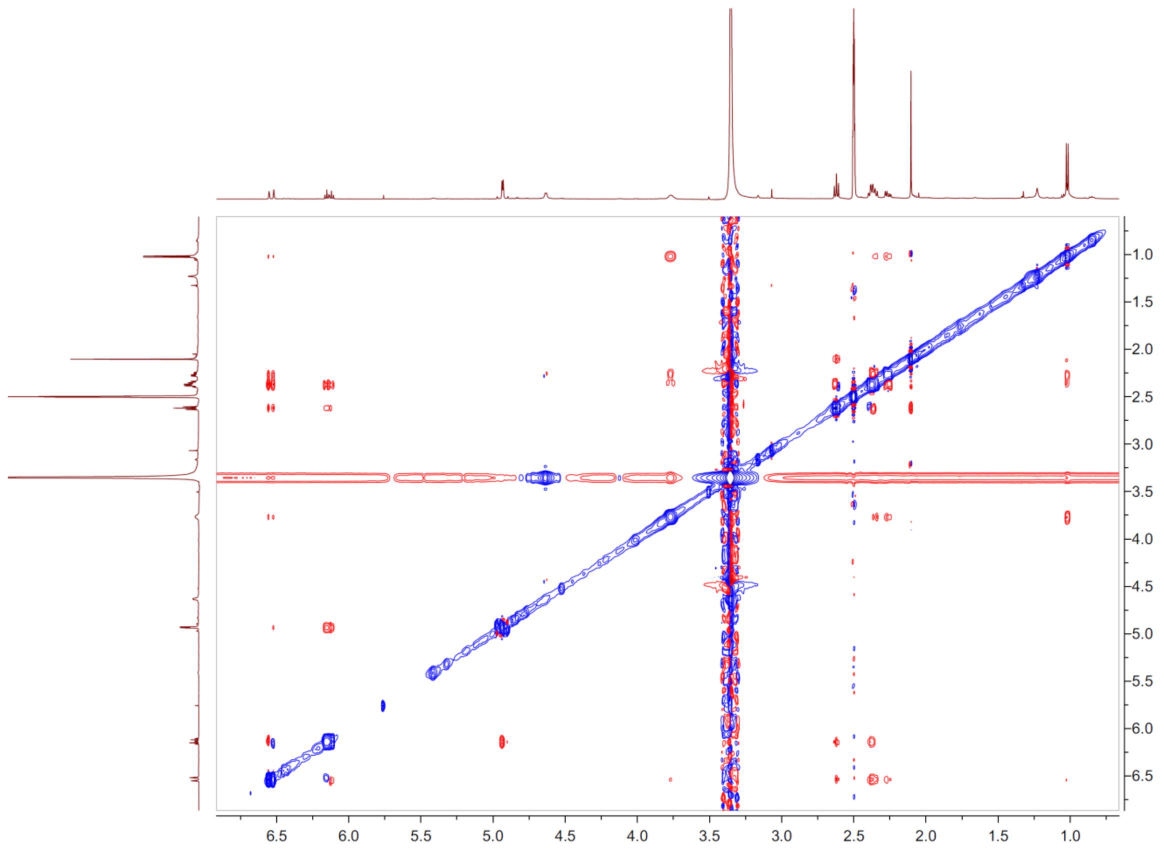

Figure S6. NOESY spectrum of compound **1** in DMSO-d_6.

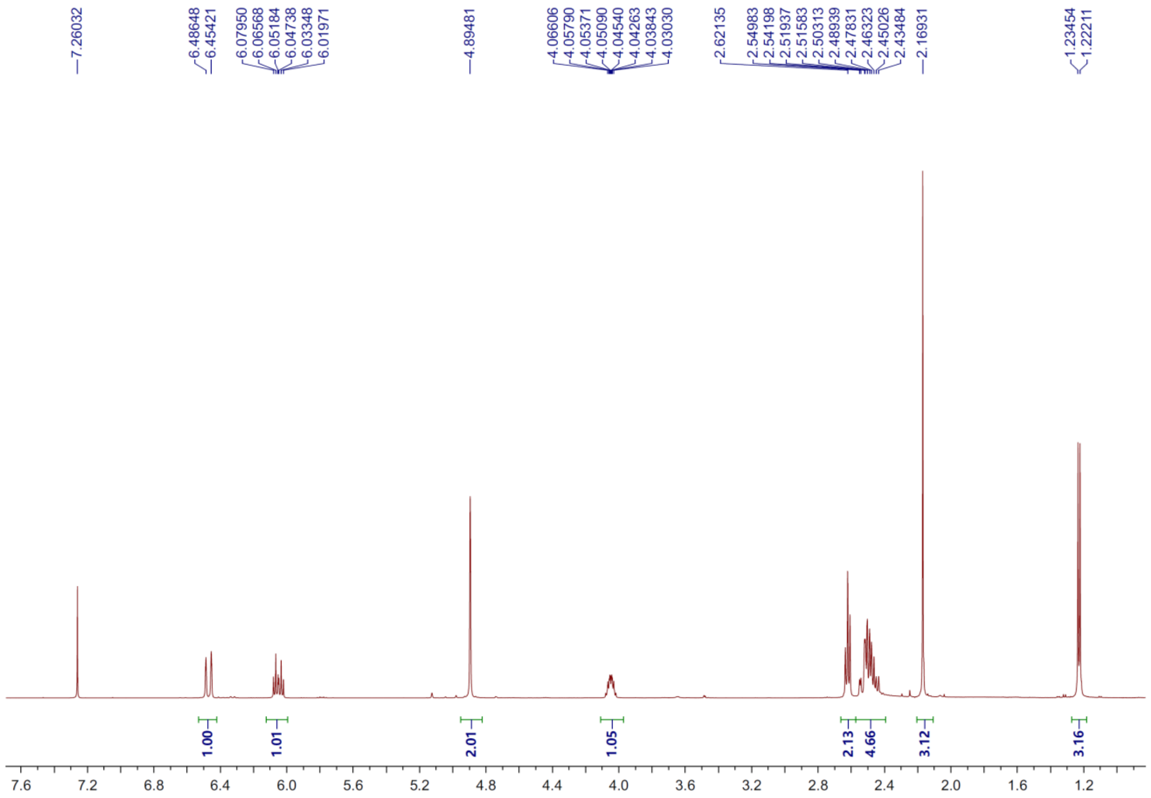

Figure S7. ^{1}H NMR spectrum of compound **1** in $CDCl_3$.

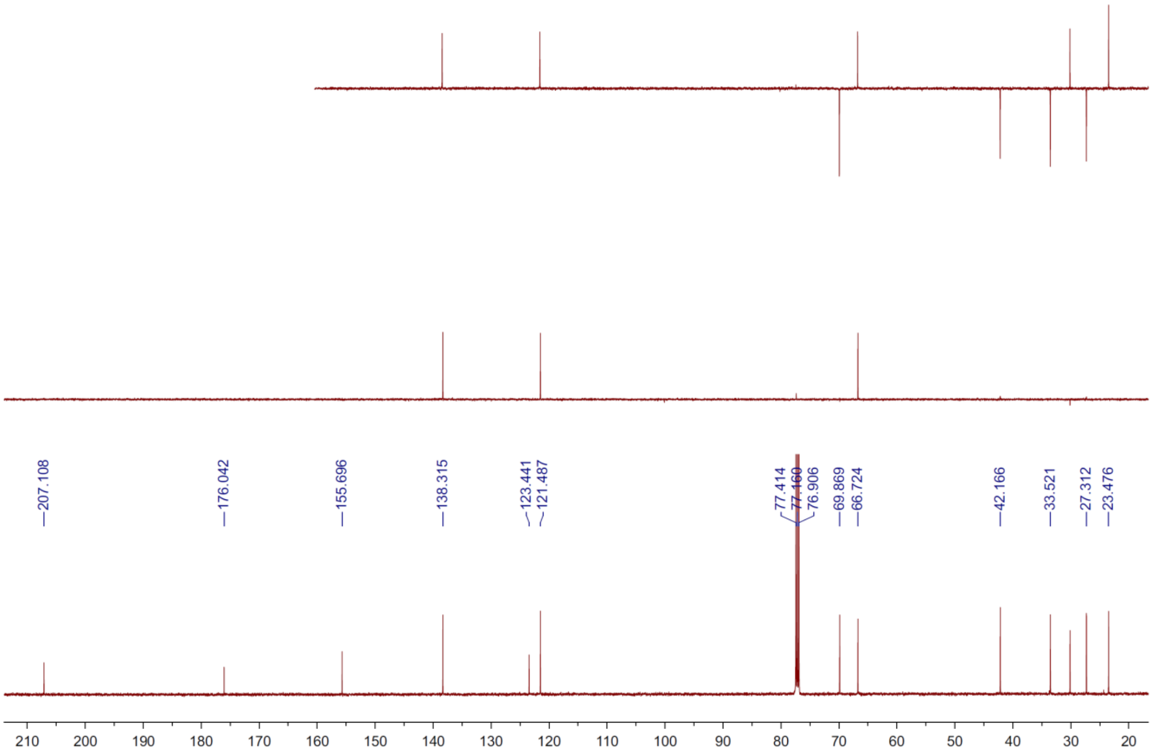

Figure S8. ^{13}C NMR and DEPT spectra of compound **1** in $CDCl_3$.

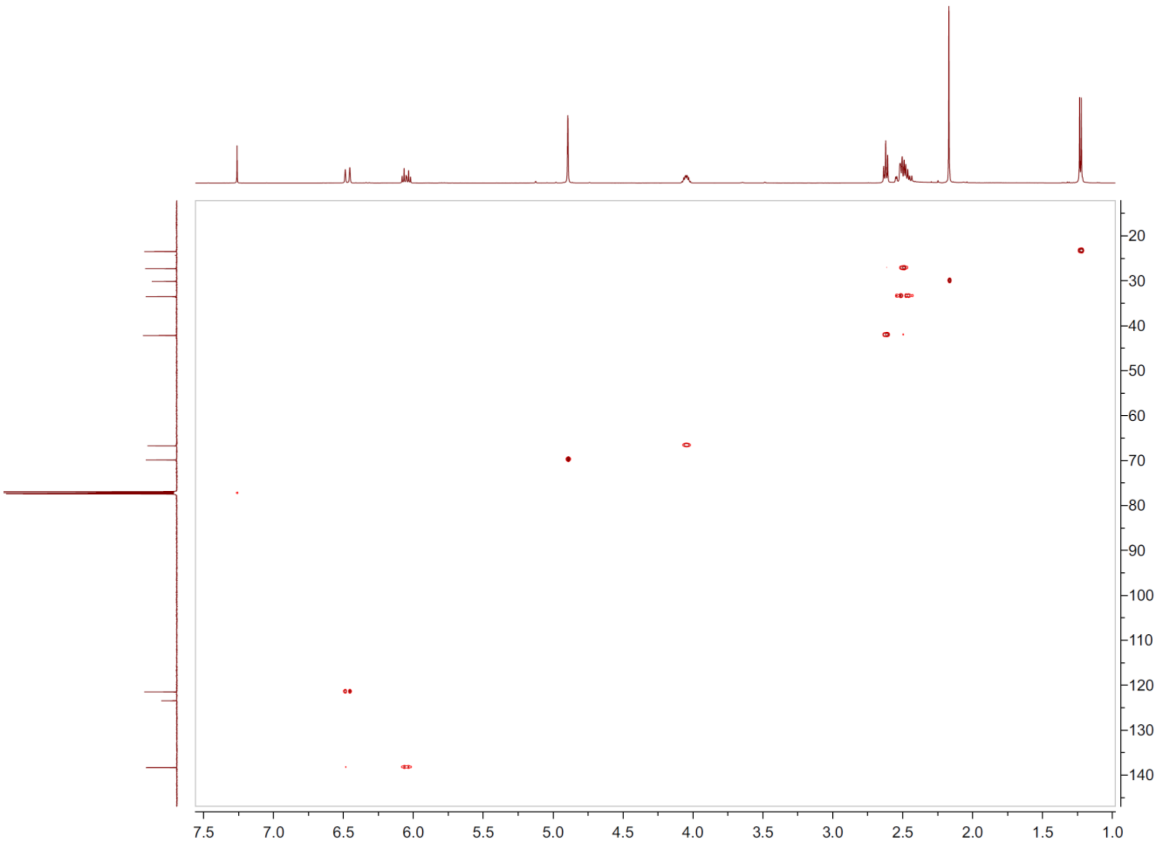

Figure S9. HSQC spectrum of compound **1** in $CDCl_3$.

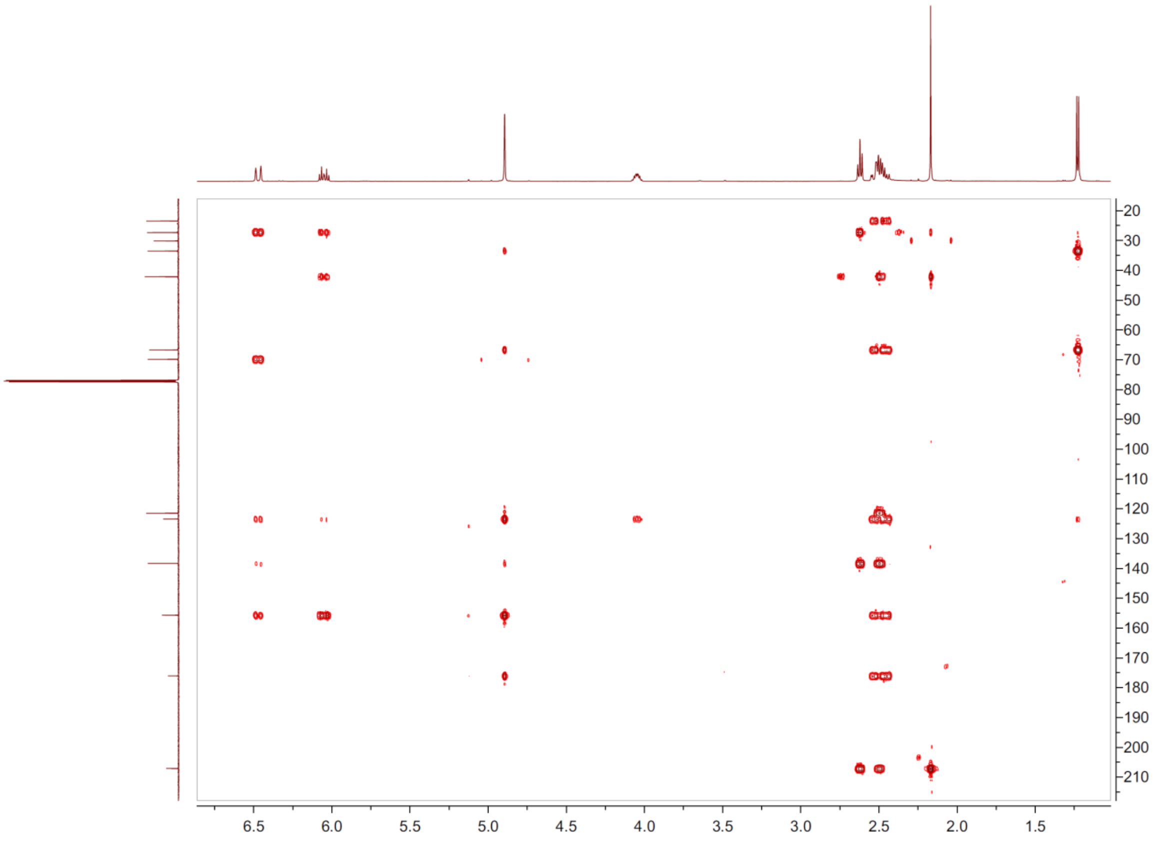

Figure S10. HMBC spectrum of compound **1** in $CDCl_3$.

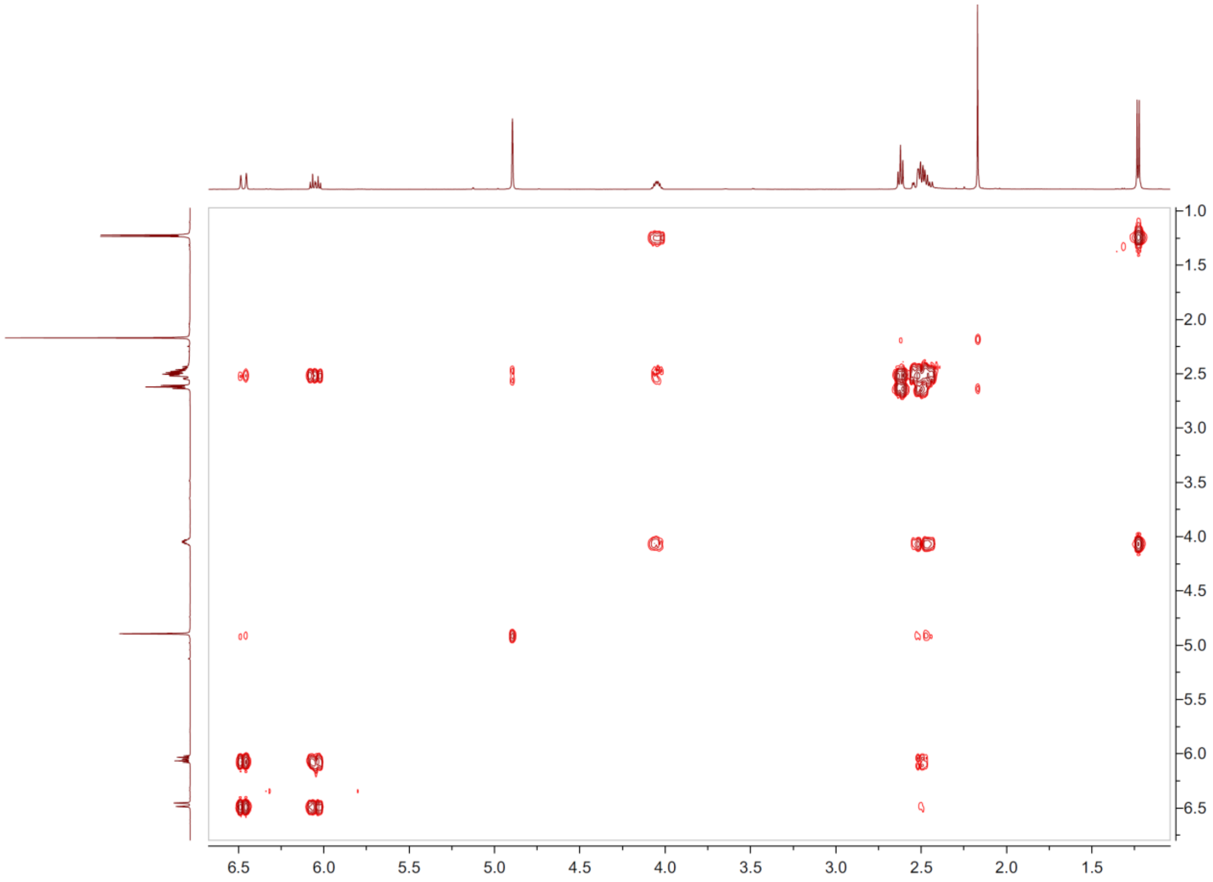

Figure S11. COSY spectrum of compound **1** in $CDCl_3$.

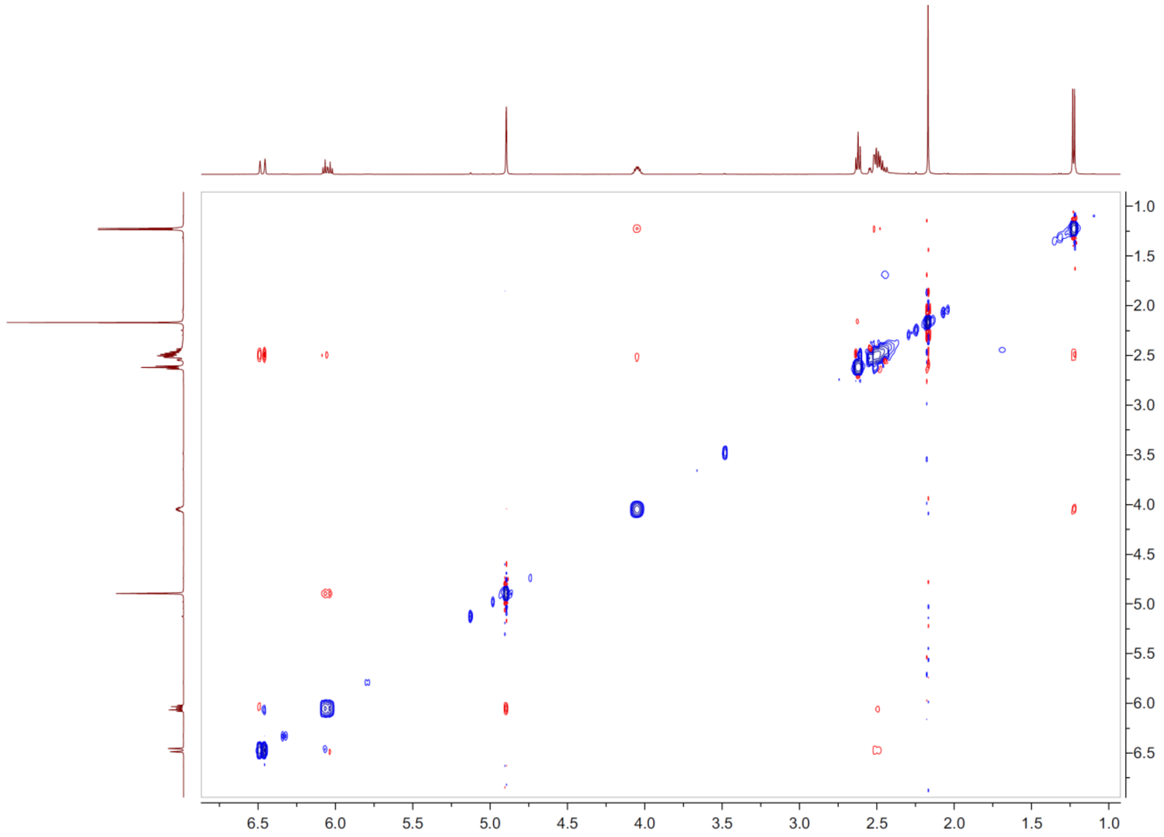

Figure S12. NOESY spectrum of compound **1** in $CDCl_3$.

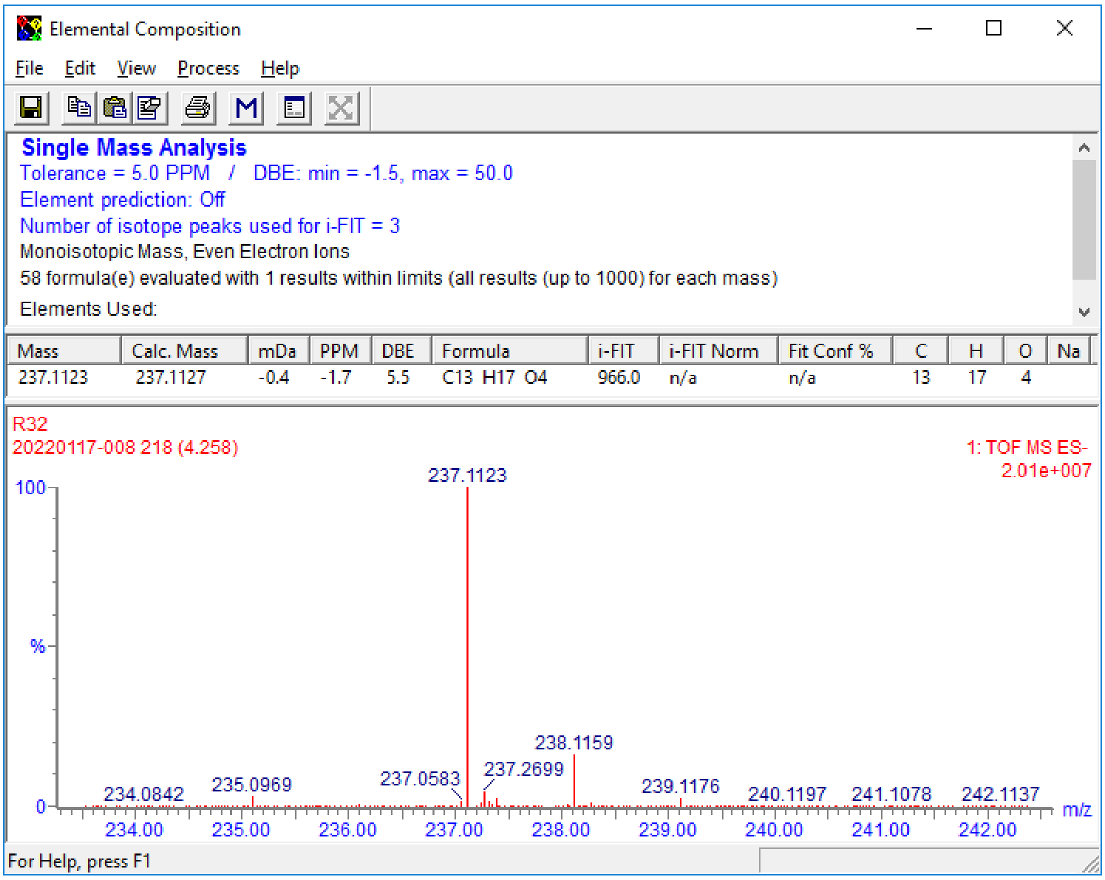

Figure S13. HRESI(-)MS spectrum of compound **1**.

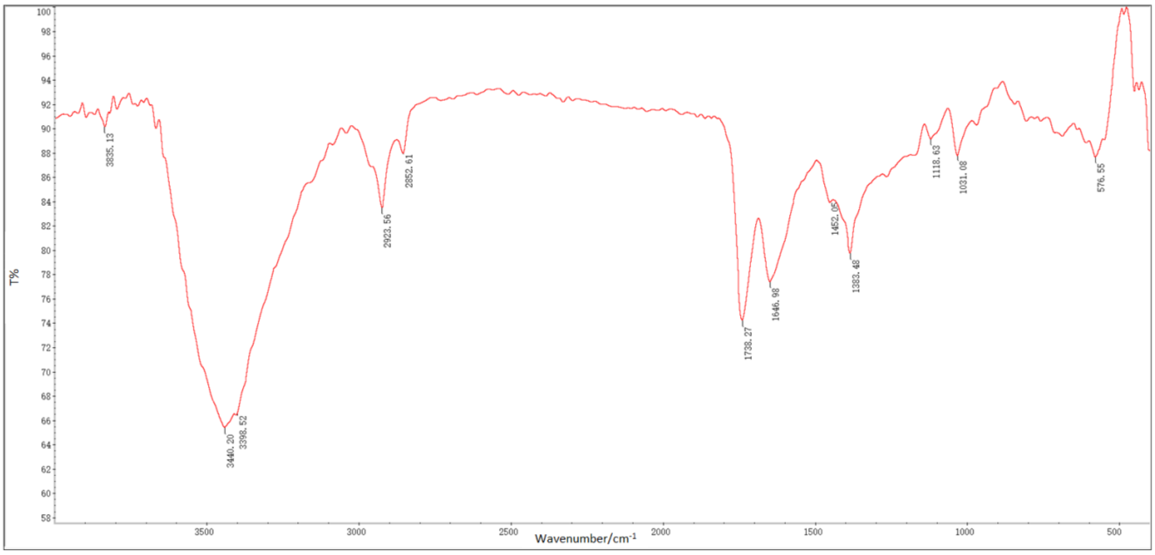

Figure S14. IR spectrum of compound **1**.

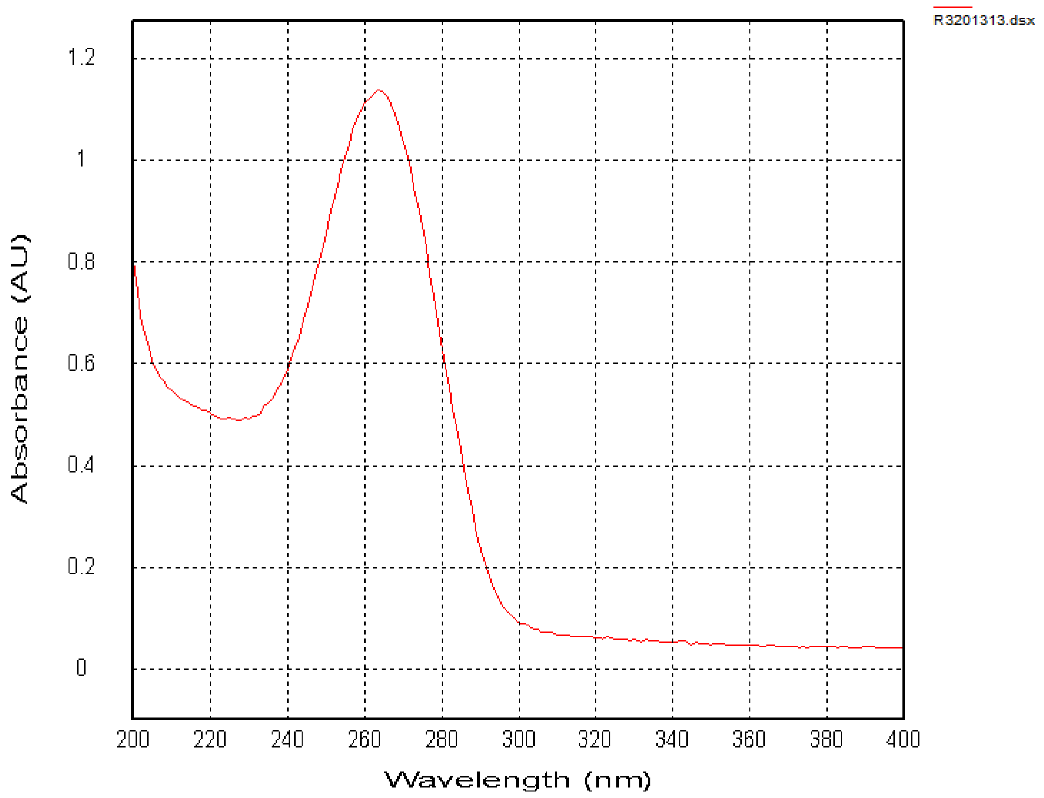

Figure S15. UV spectrum of compound **1** (0.2 mg/mL in MeOH).

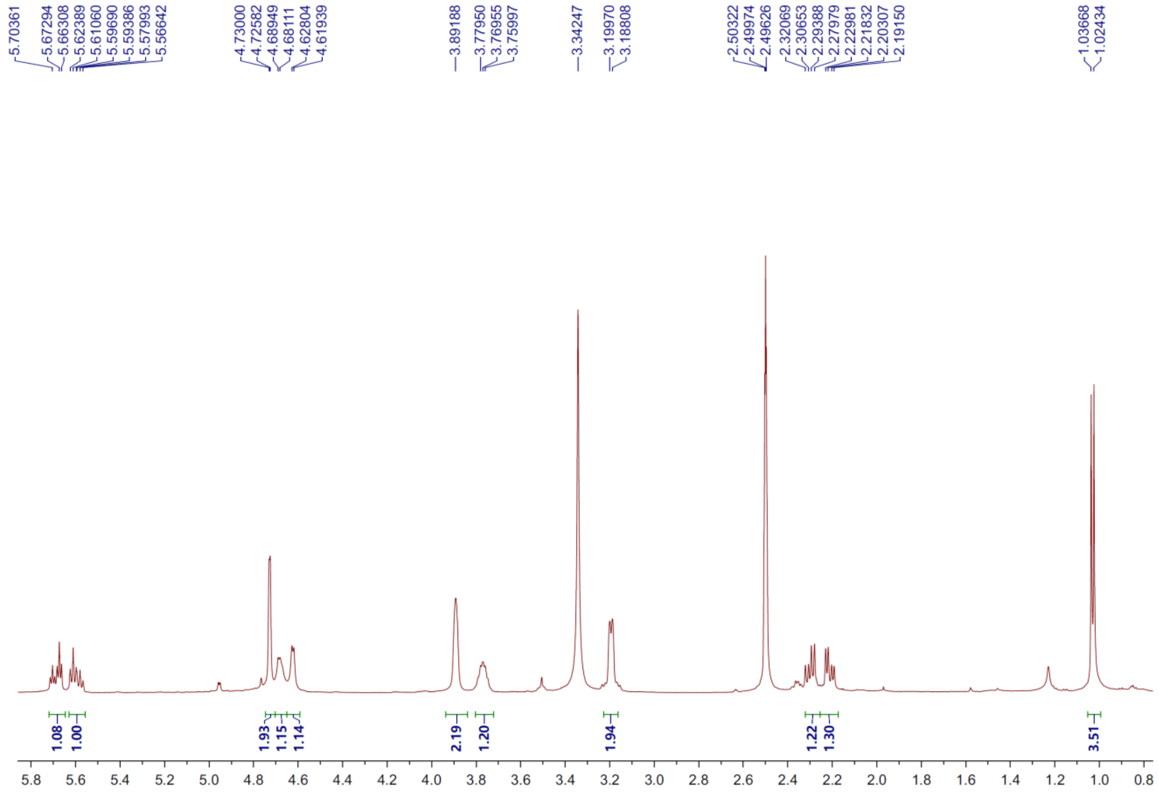

Figure S16. ^{1}H NMR spectrum of compound **2** in DMSO-d_6.

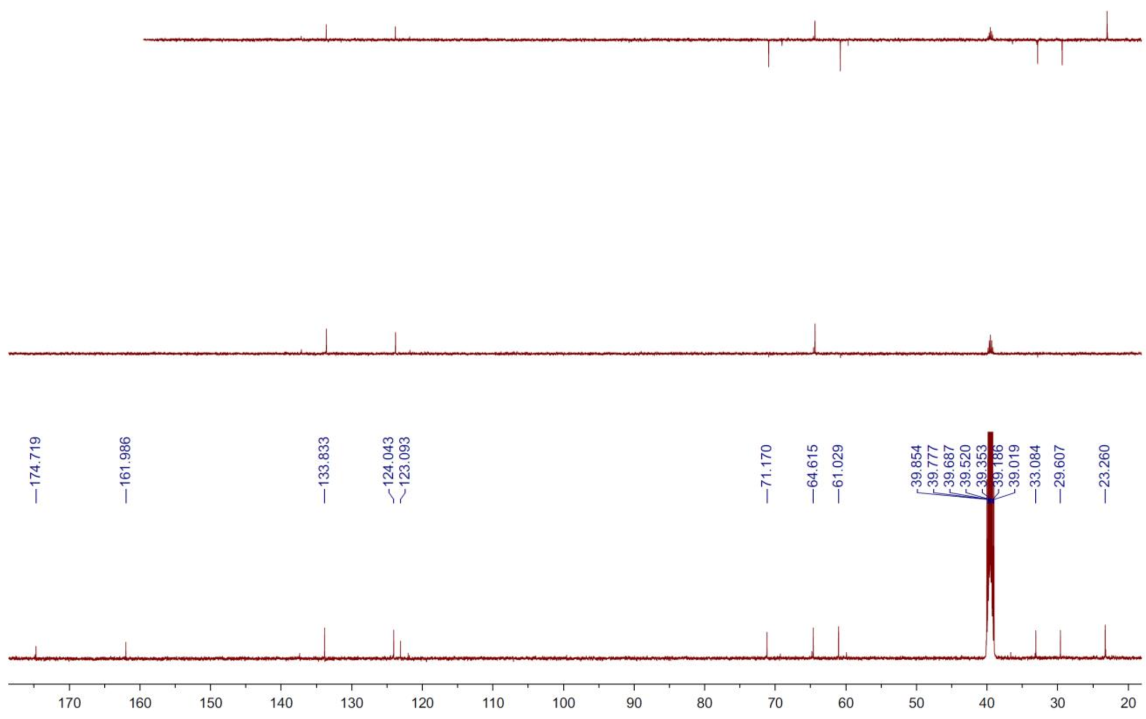

Figure S17. ^{13}C NMR and DEPT spectra of compound **2** in DMSO-d_6.

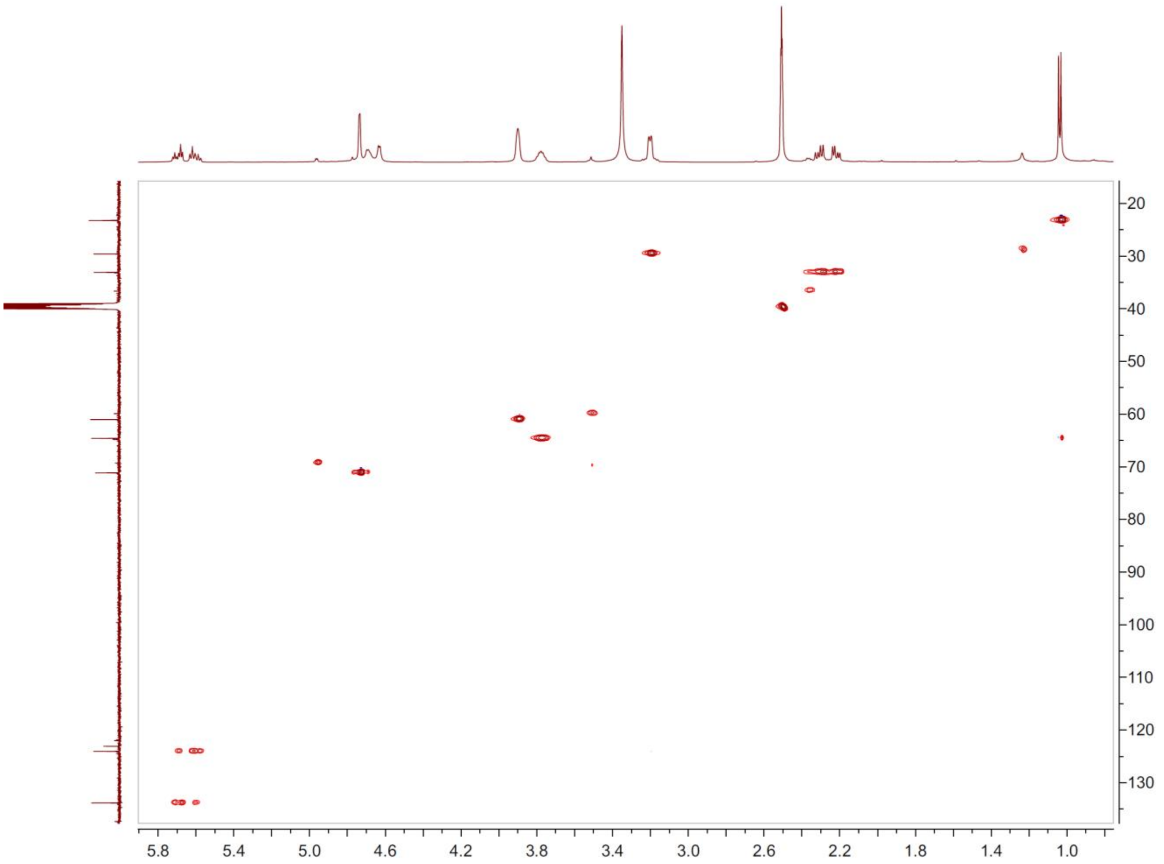

Figure S18. HSQC spectrum of compound **2** in DMSO-d_6.

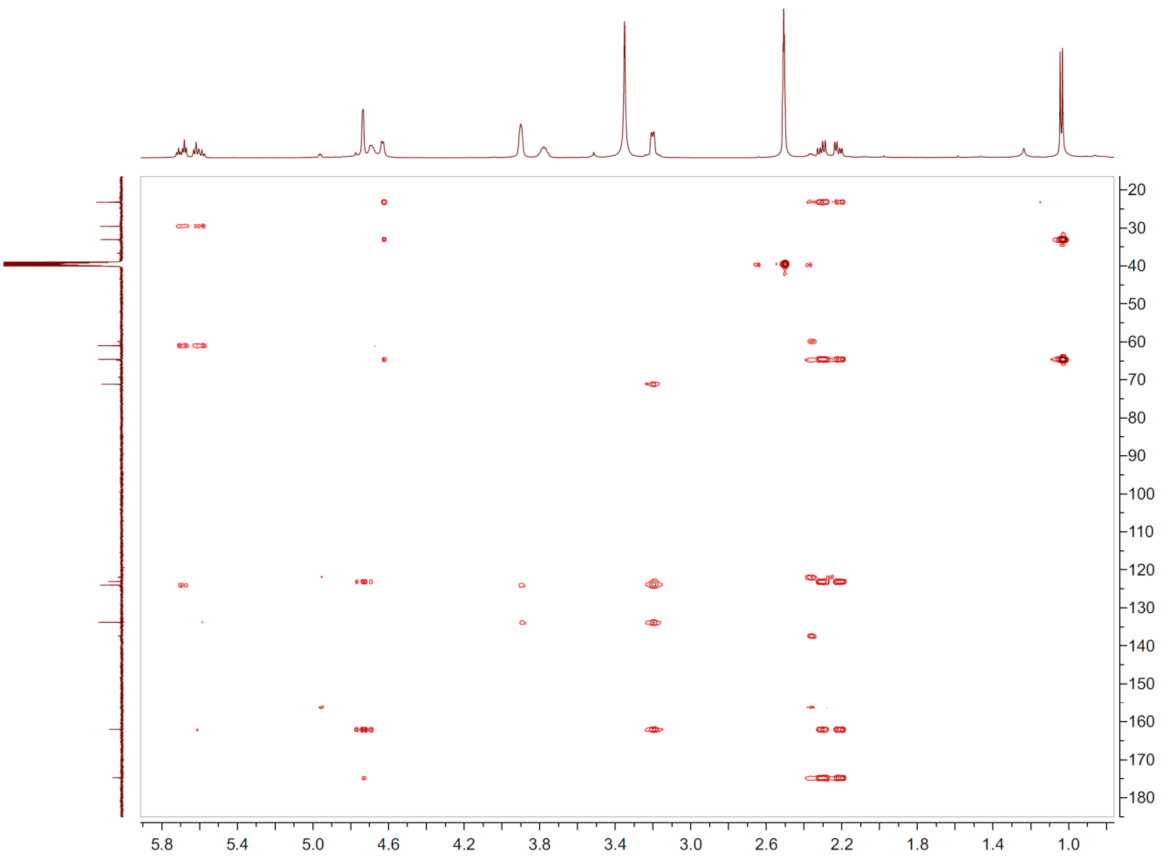

Figure S19. HMBC spectrum of compound **2** in DMSO-d_6.

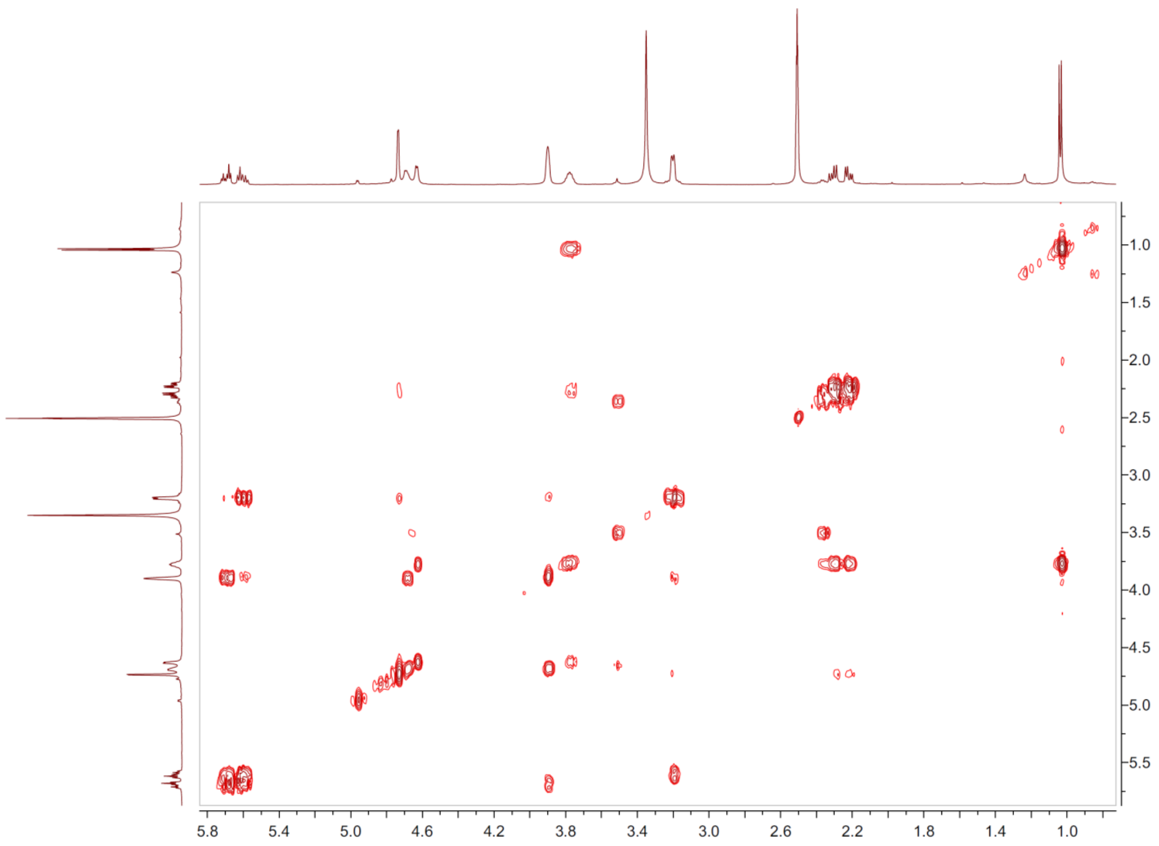

Figure S20. COSY spectrum of compound **2** in DMSO-d_6.

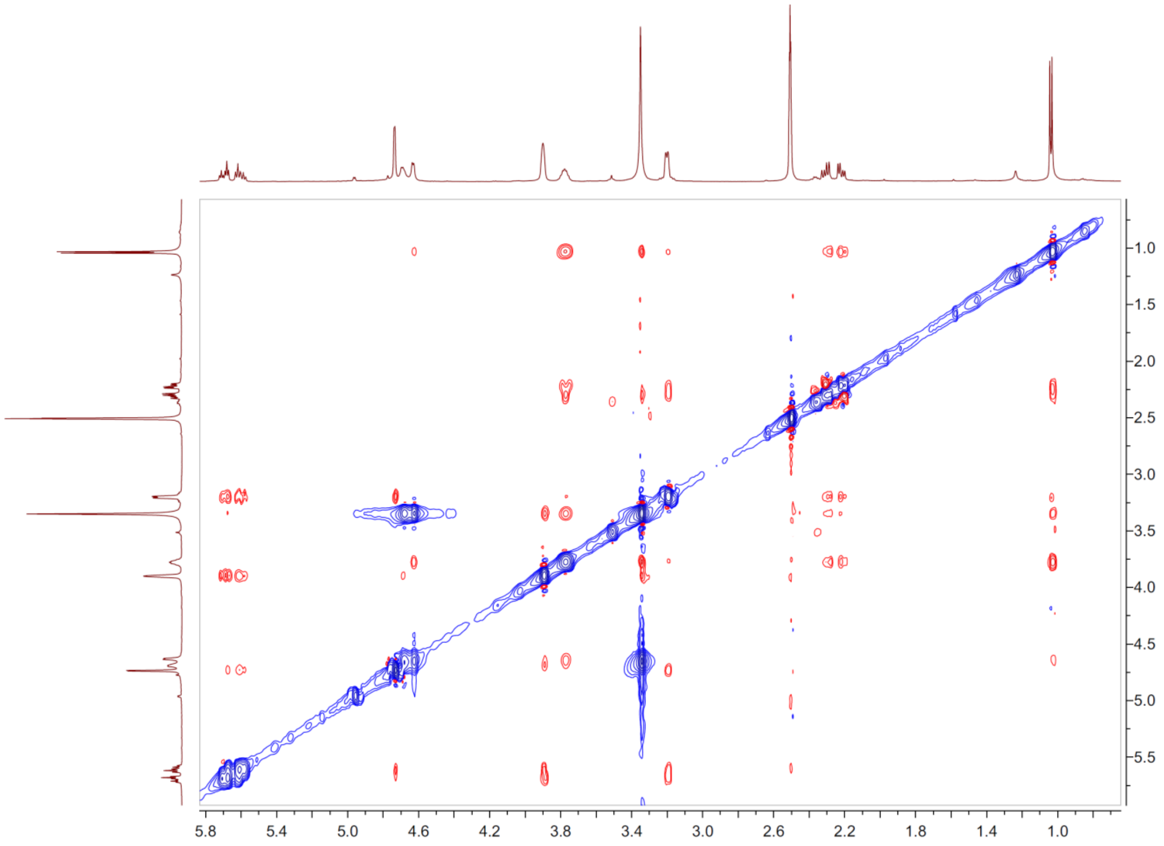

Figure S21. NOESY spectrum of compound **2** in DMSO-d_6.

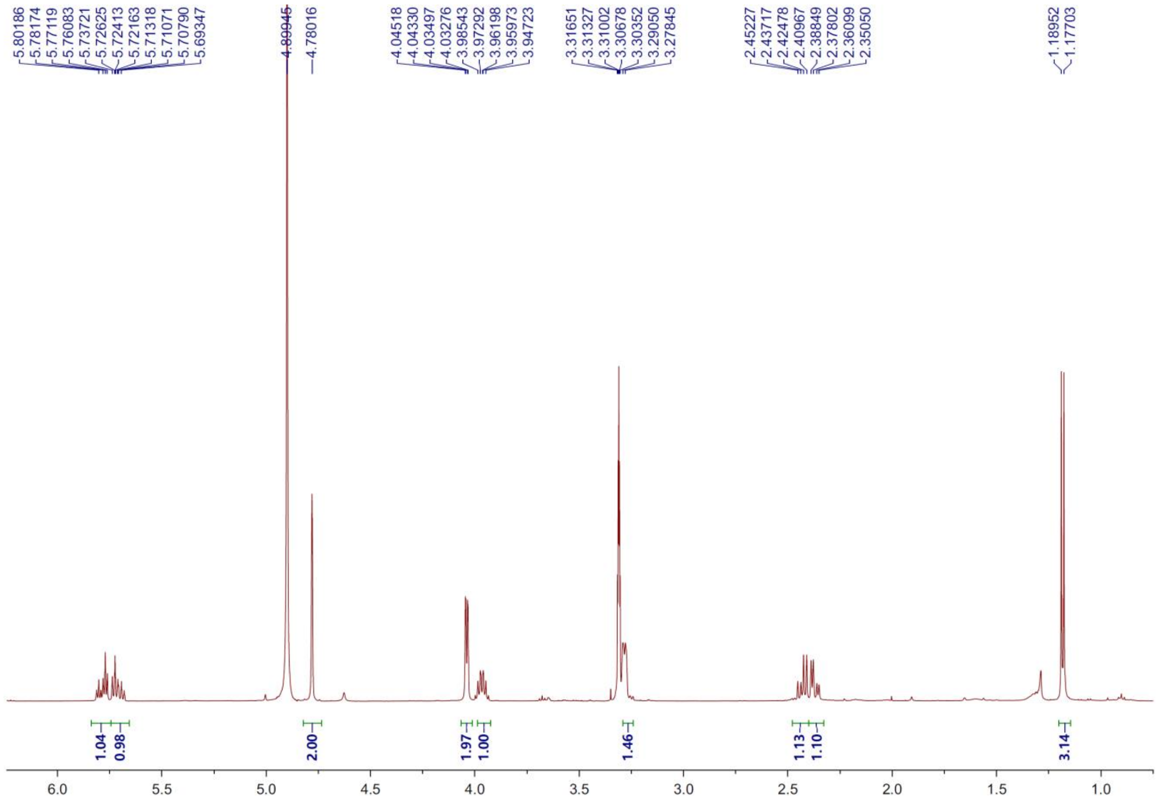

Figure S22. ^1H NMR spectrum of compound **2** in CD_3OD.

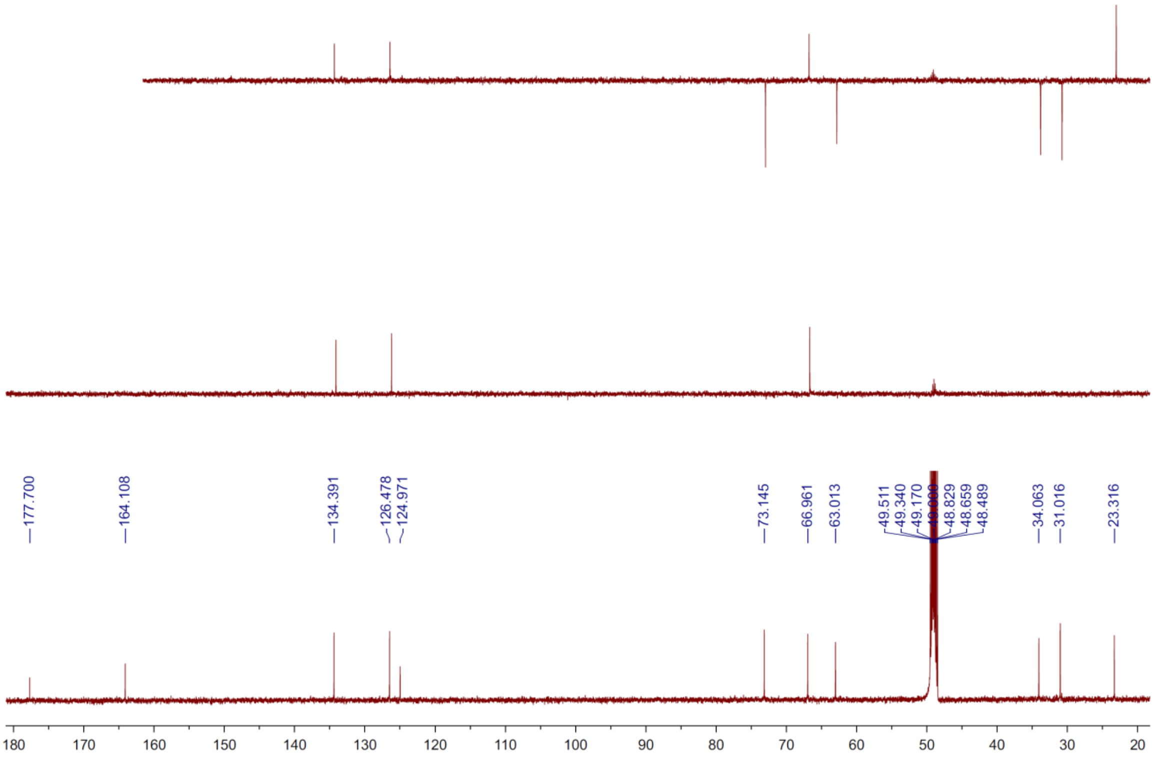

Figure S23. ^{13}C NMR and DEPT spectra of compound **2** in CD_3OD.

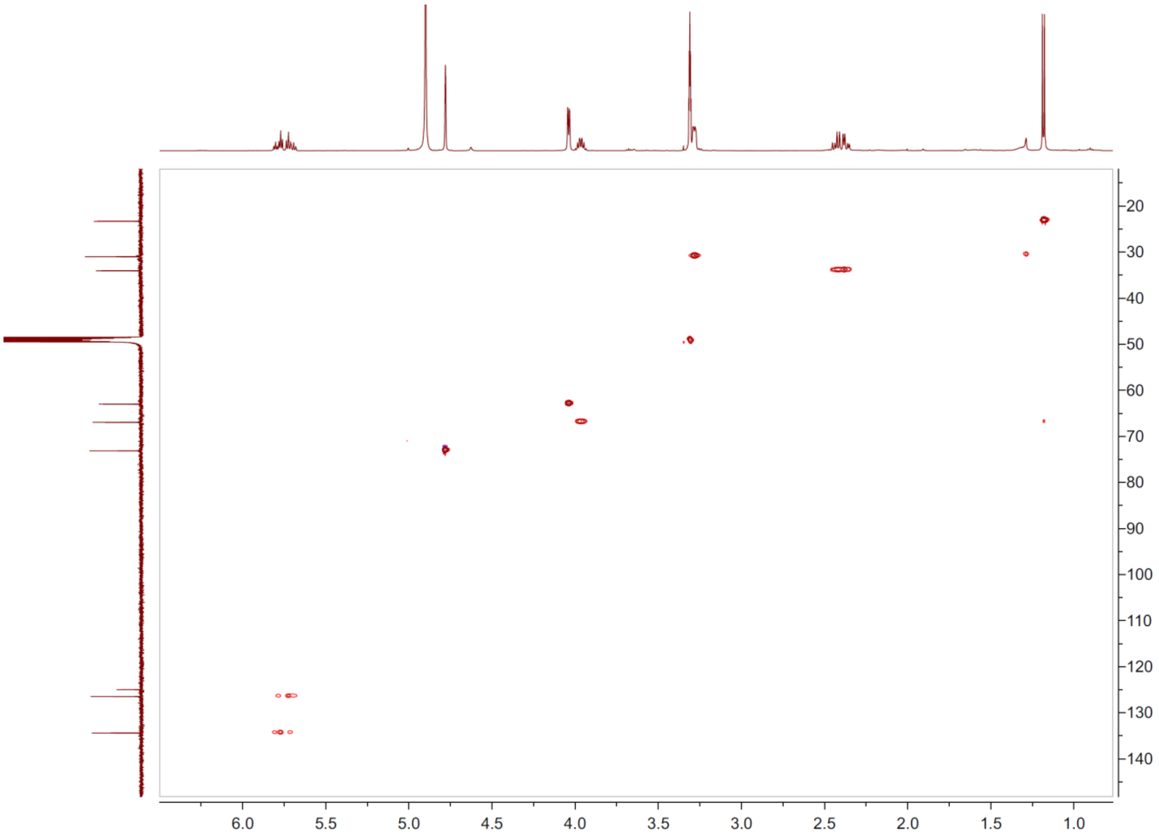

Figure S24. HSQC spectrum of compound **2** in CD_3OD.

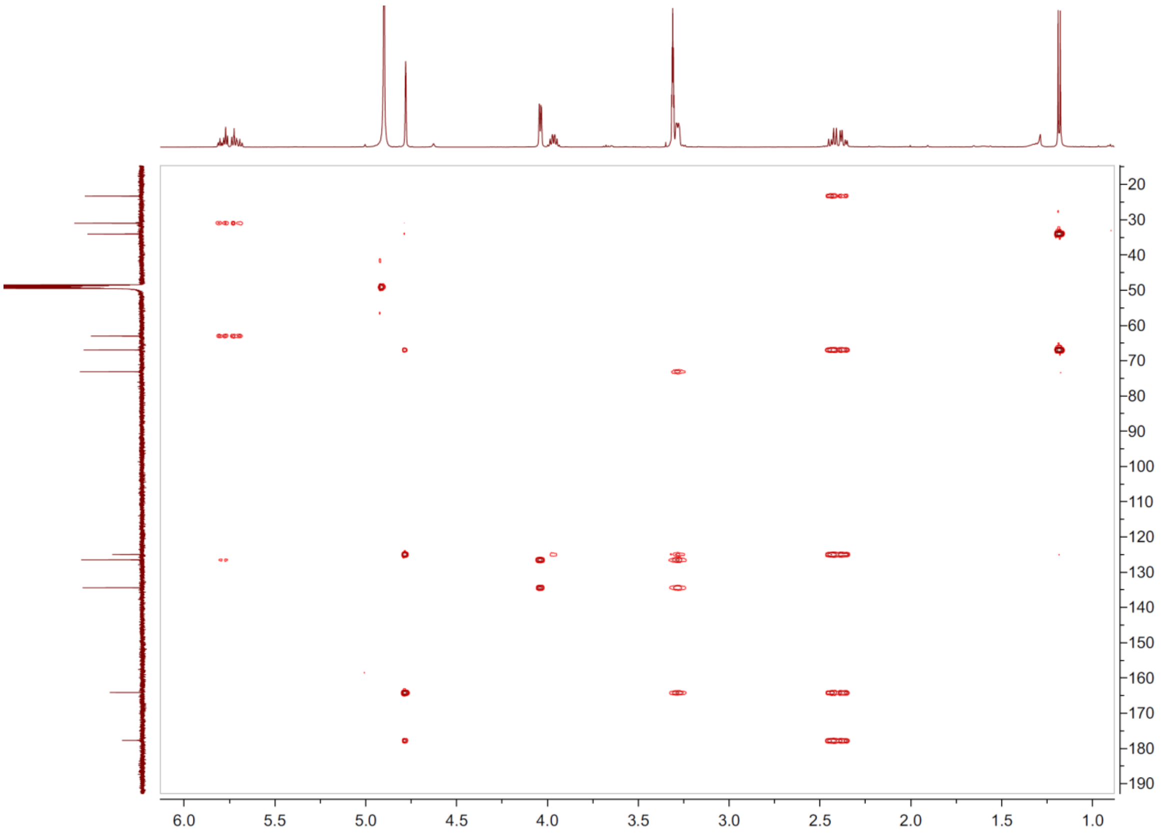

Figure S25. HMBC spectrum of compound **2** in CD_3OD.

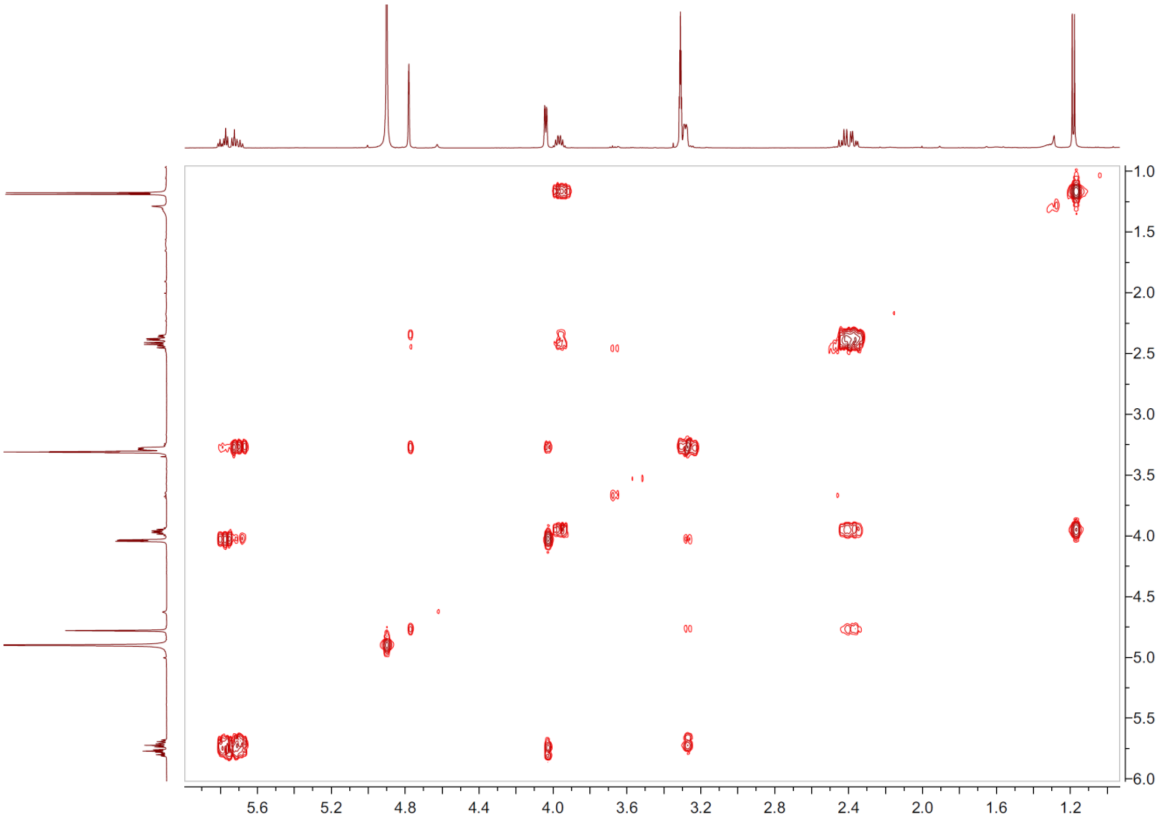

Figure S26. COSY spectrum of compound **2** in CD_3OD.

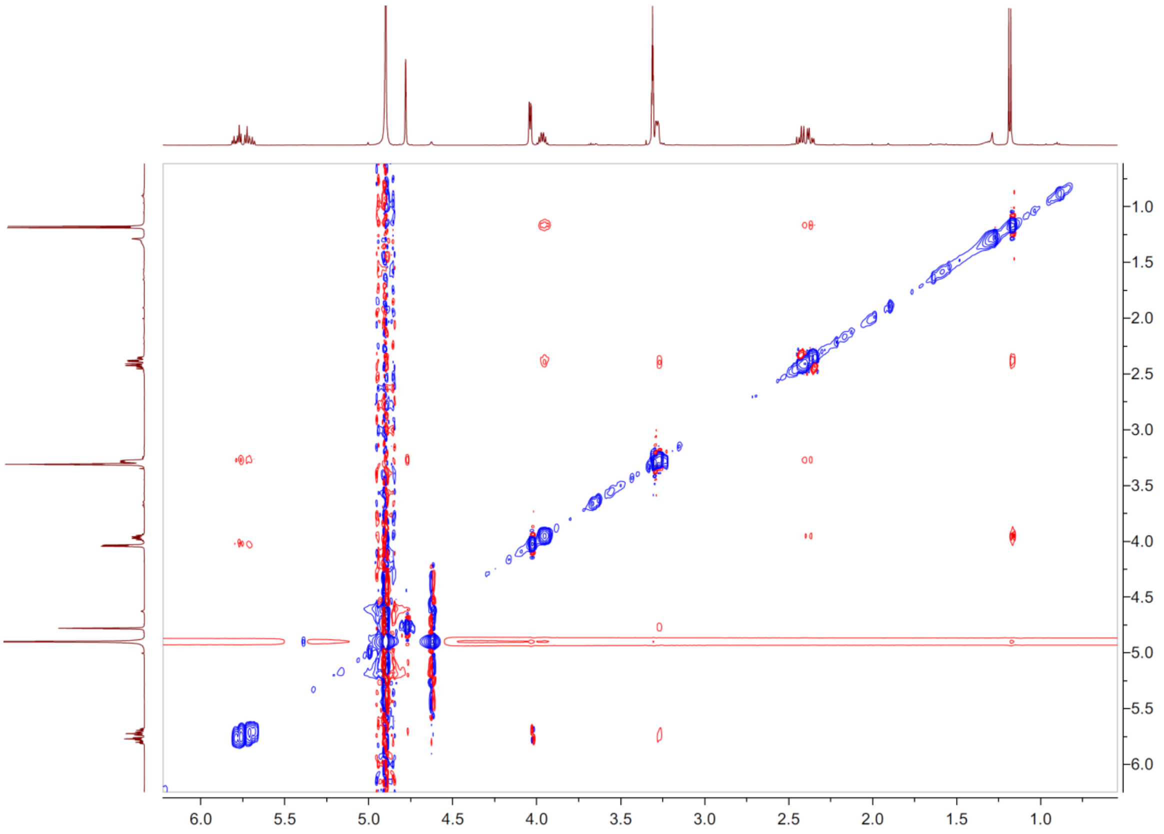

Figure S27. NOESY spectrum of compound **2** in CD_3OD.

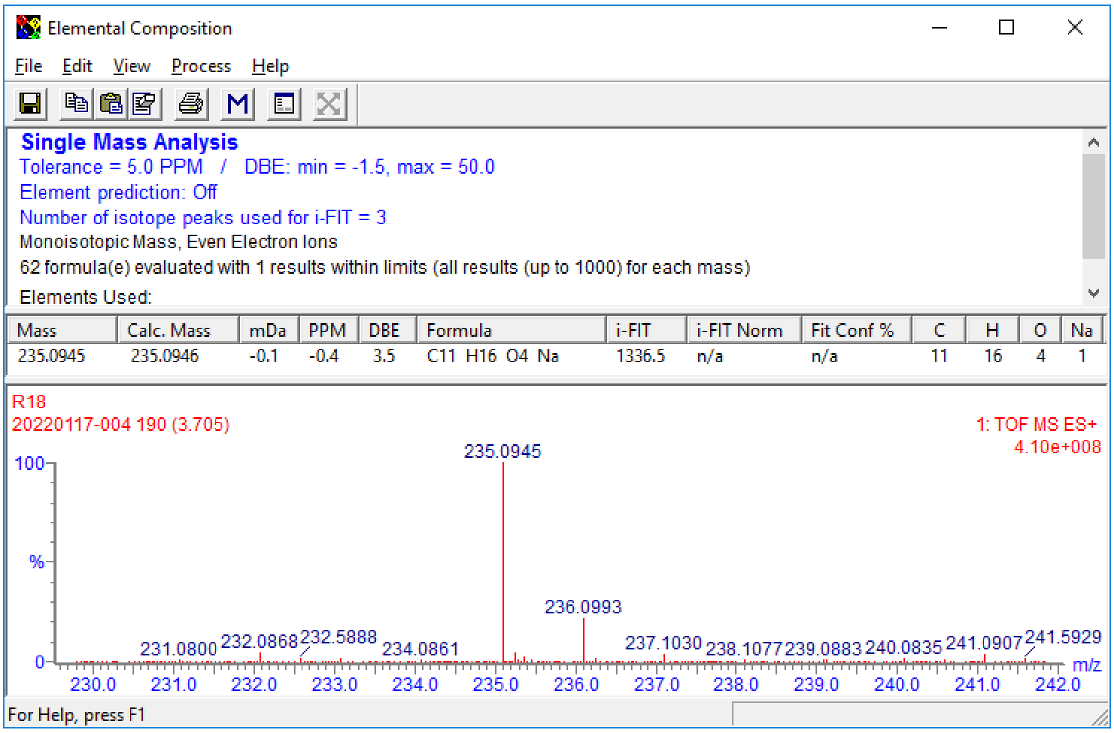

Figure S28. HRESI(+)MS spectrum of compound **2**.

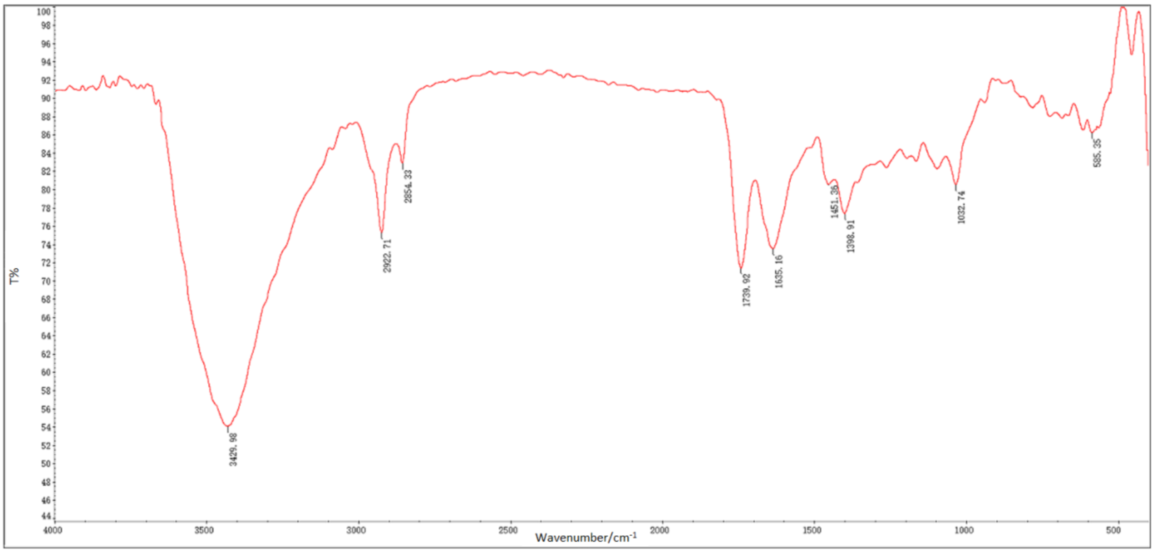

Figure S29. IR spectrum of compound **2**.

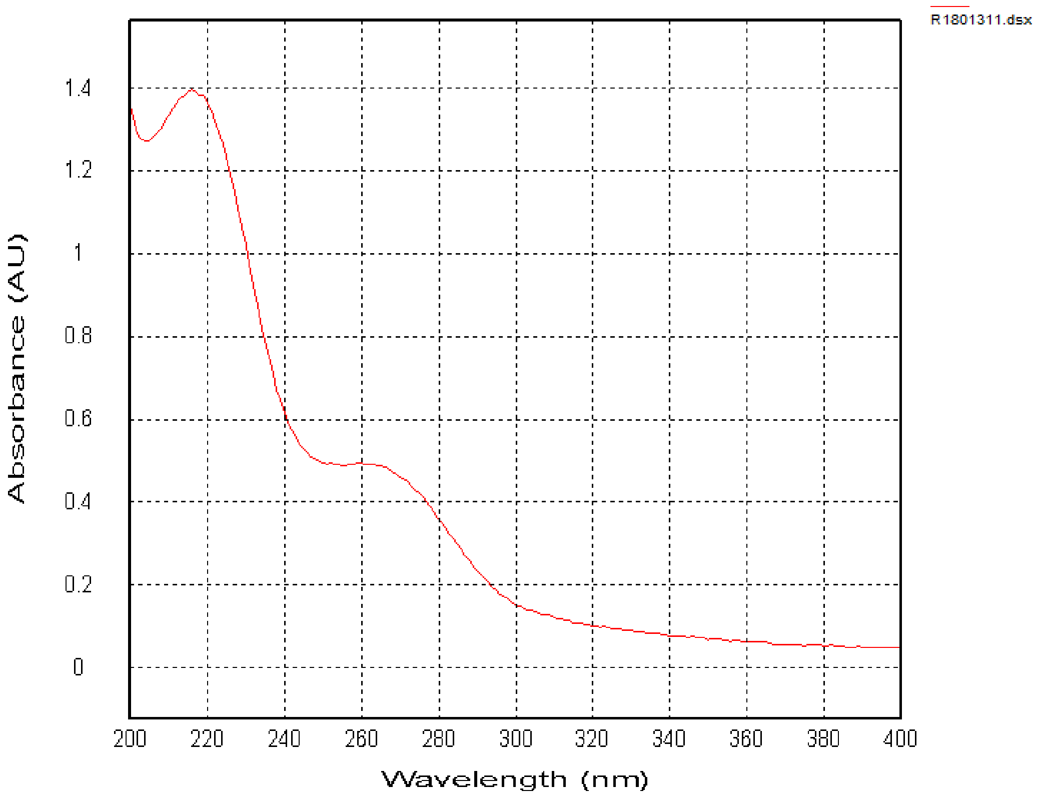

Figure S30. UV spectrum of compound **2** (0.4 mg/mL in MeOH).

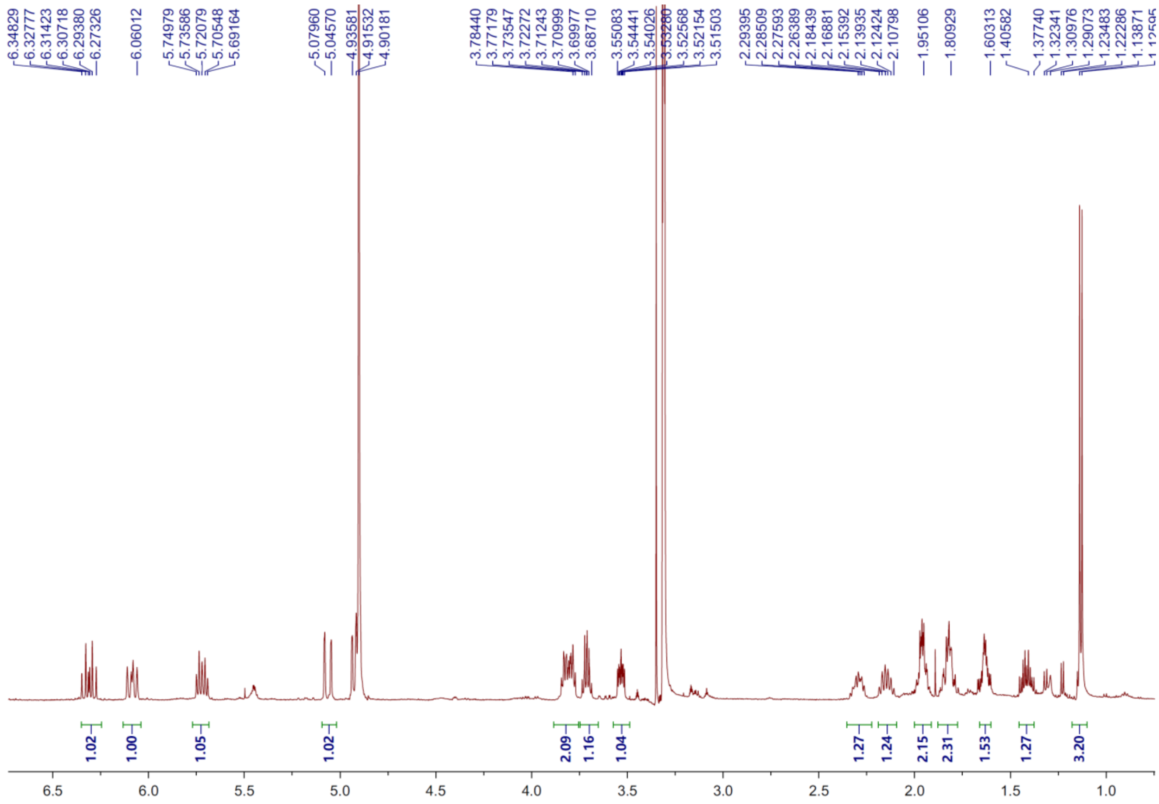

Figure S31. ^{1}H NMR spectrum of compound **3** in CD_3OD.

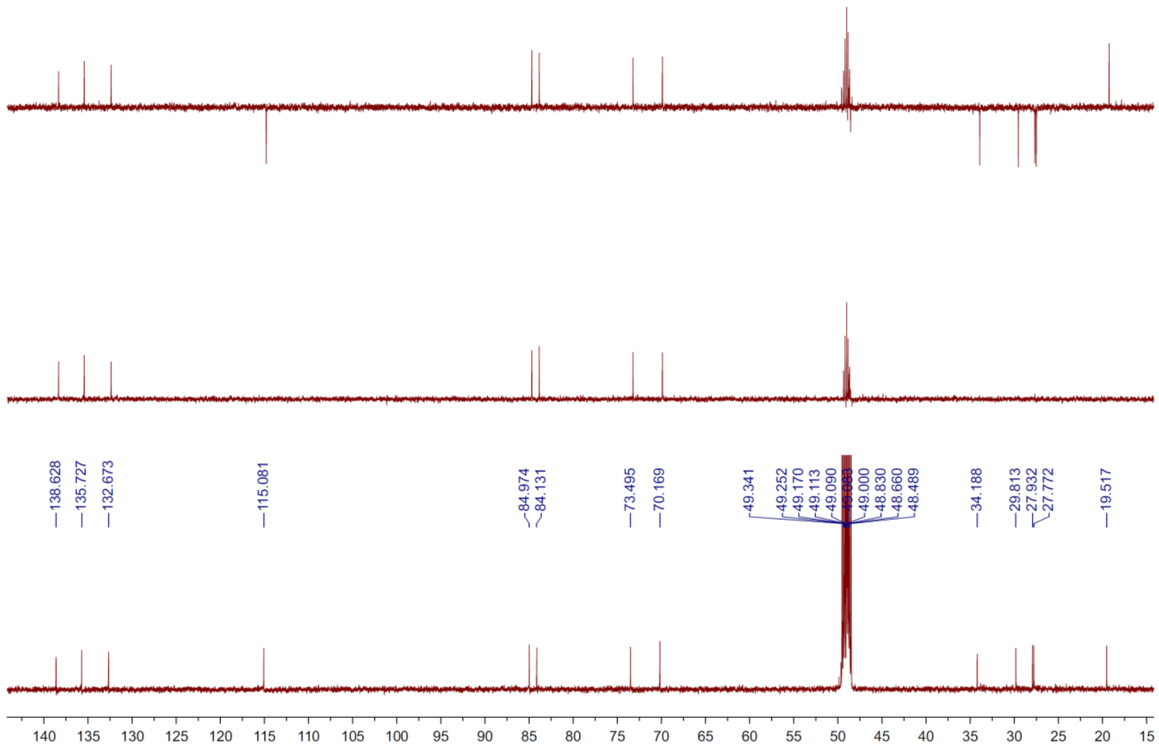

Figure S32. ^{13}C NMR and DEPT spectra of compound **3** in CD_3OD.

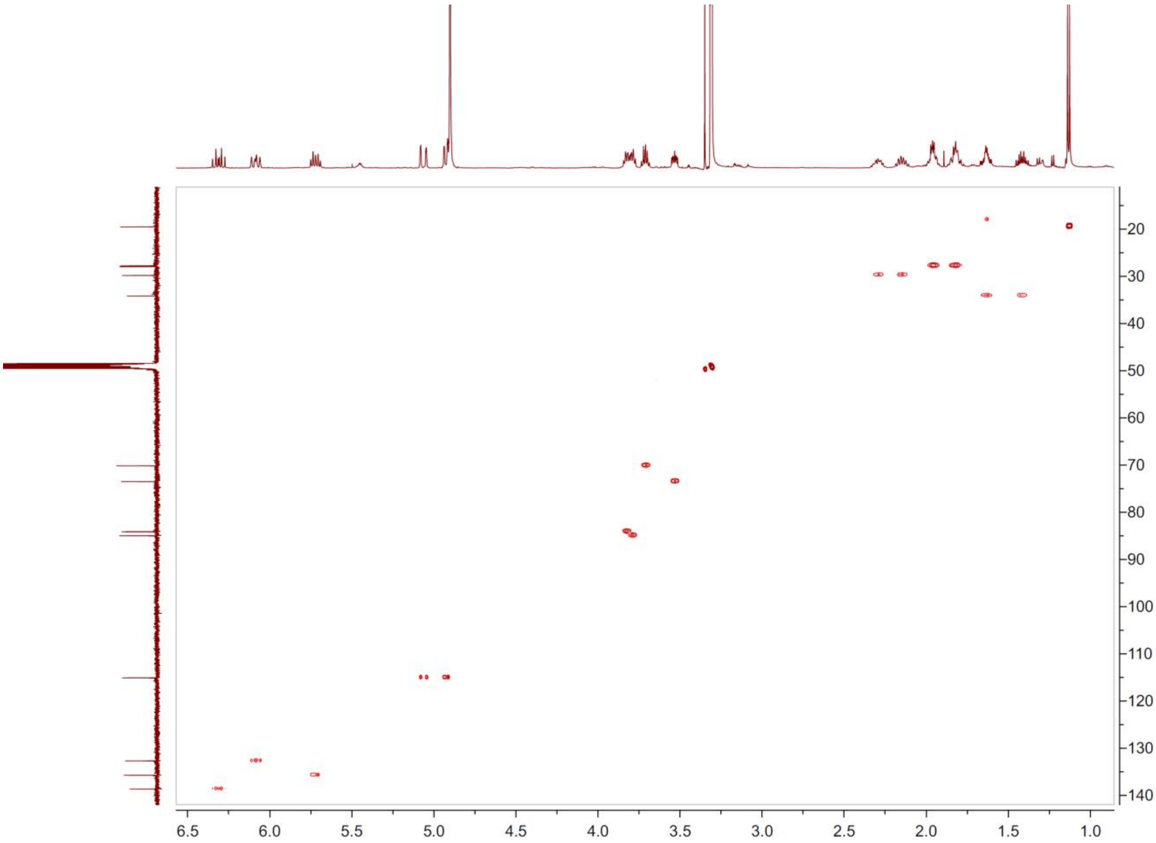

Figure S33. HSQC spectrum of compound **3** in CD_3OD.

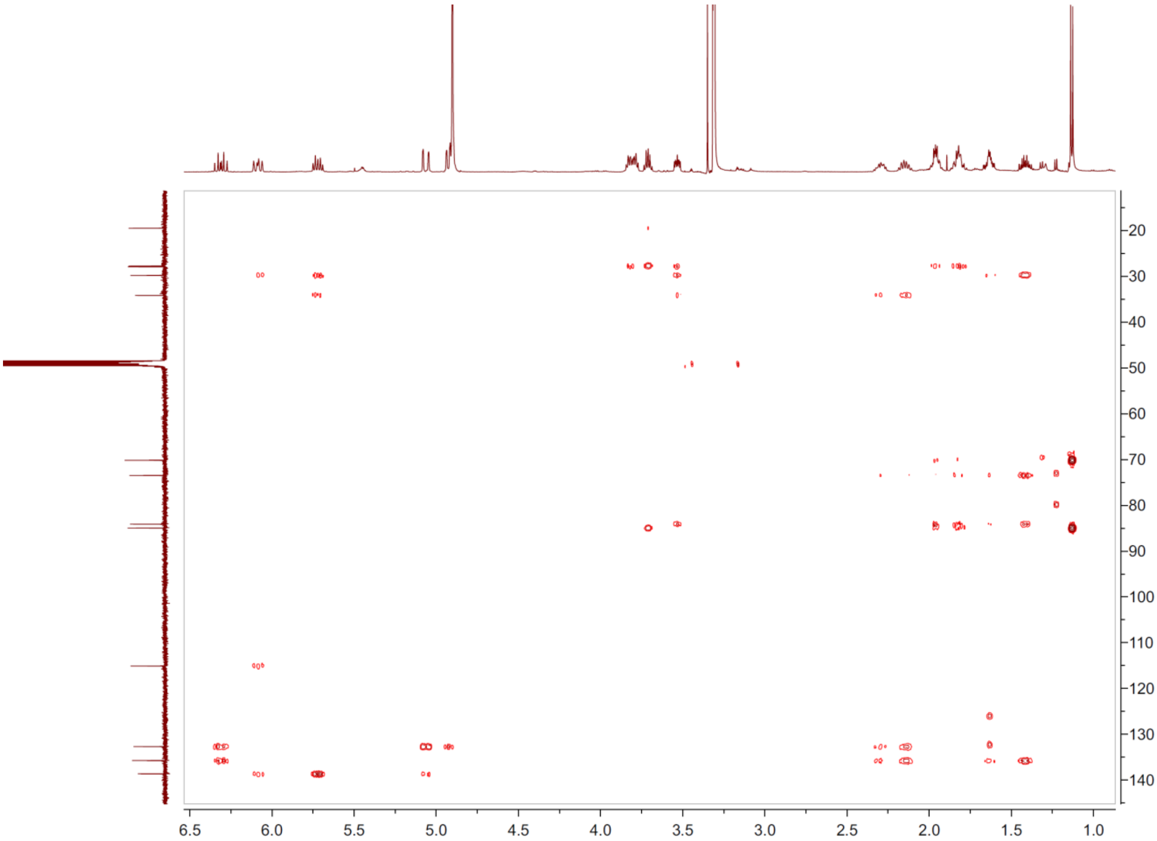

Figure S34. HMBC spectrum of compound **3** in CD_3OD.

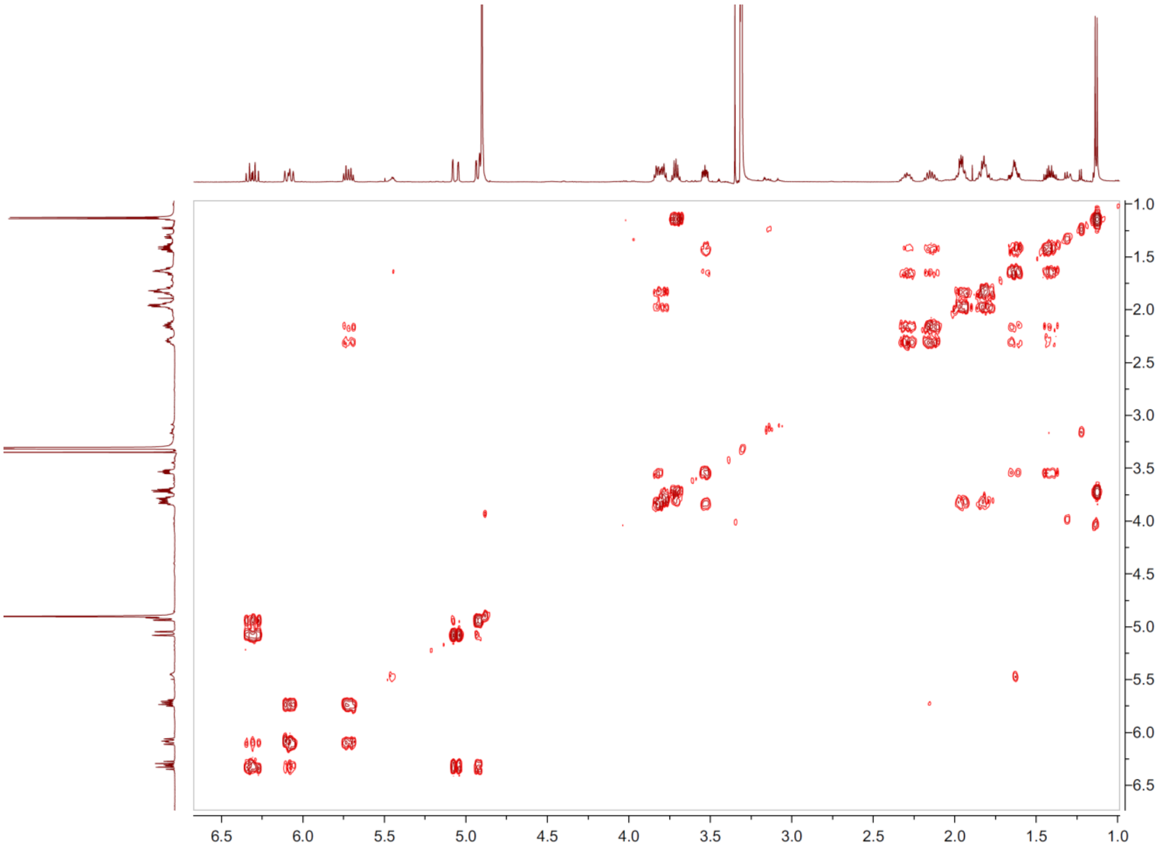

Figure S35. COSY spectrum of compound **3** in CD_3OD.

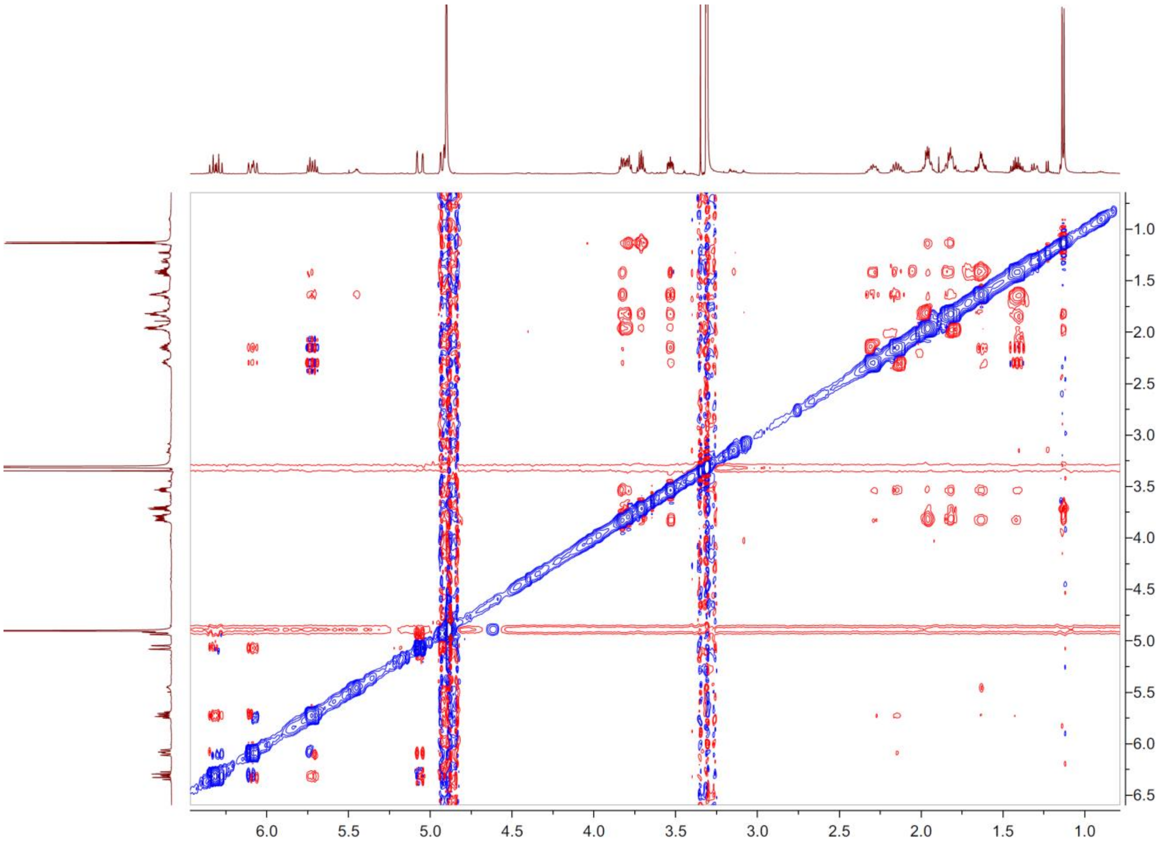

Figure S36. NOESY spectrum of compound **3** in CD_3OD.

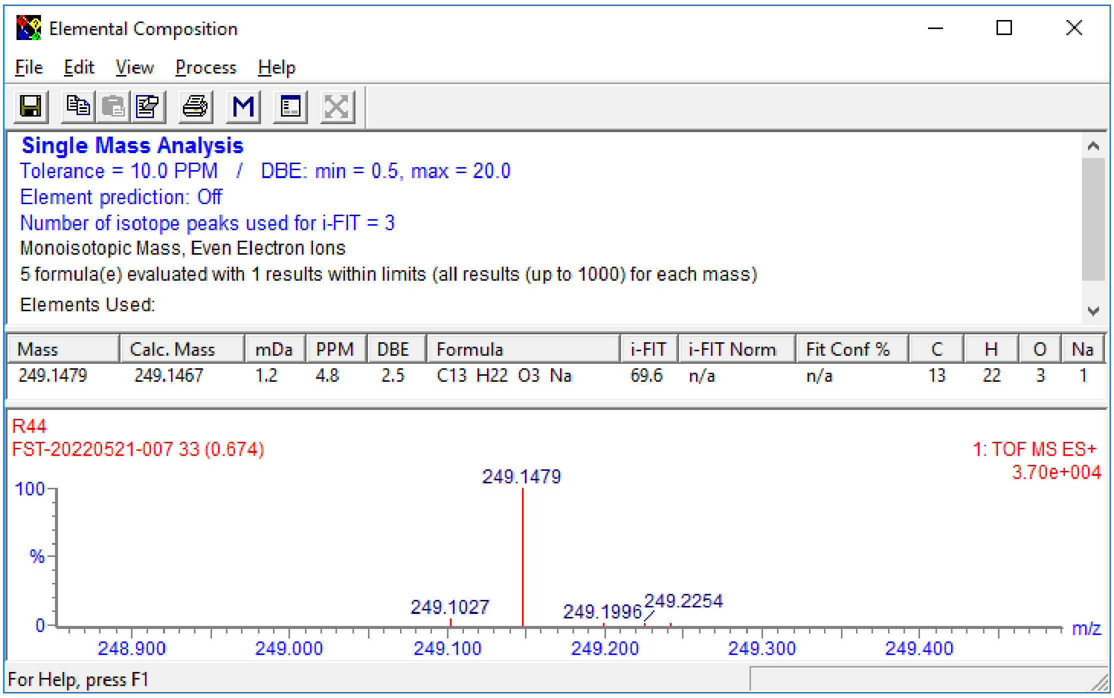

Figure S37. HRESI(+)MS spectrum of compound **3**.

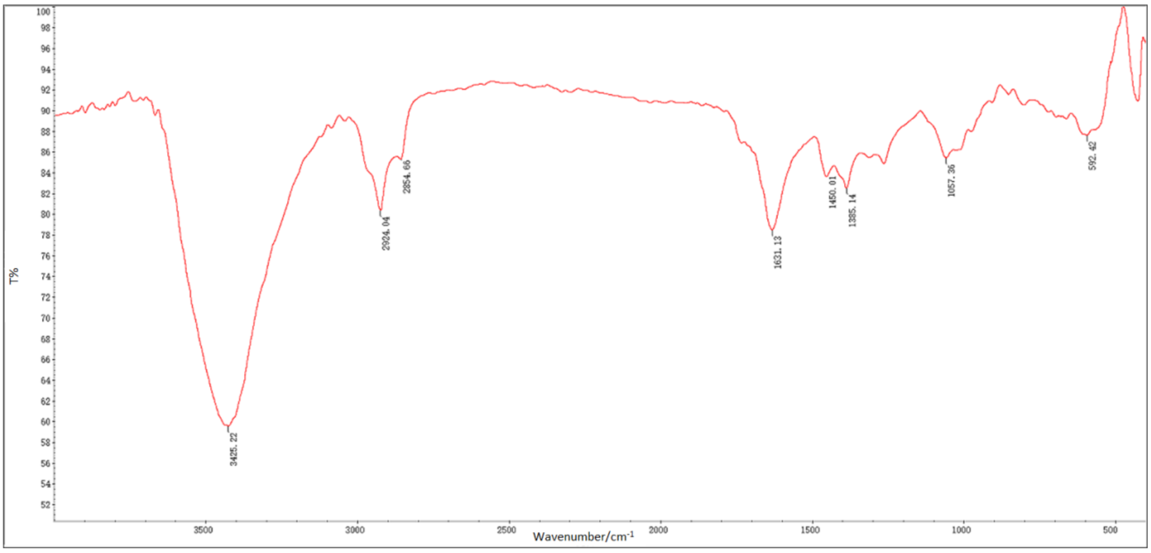

Figure S38. IR spectrum of compound **3**.

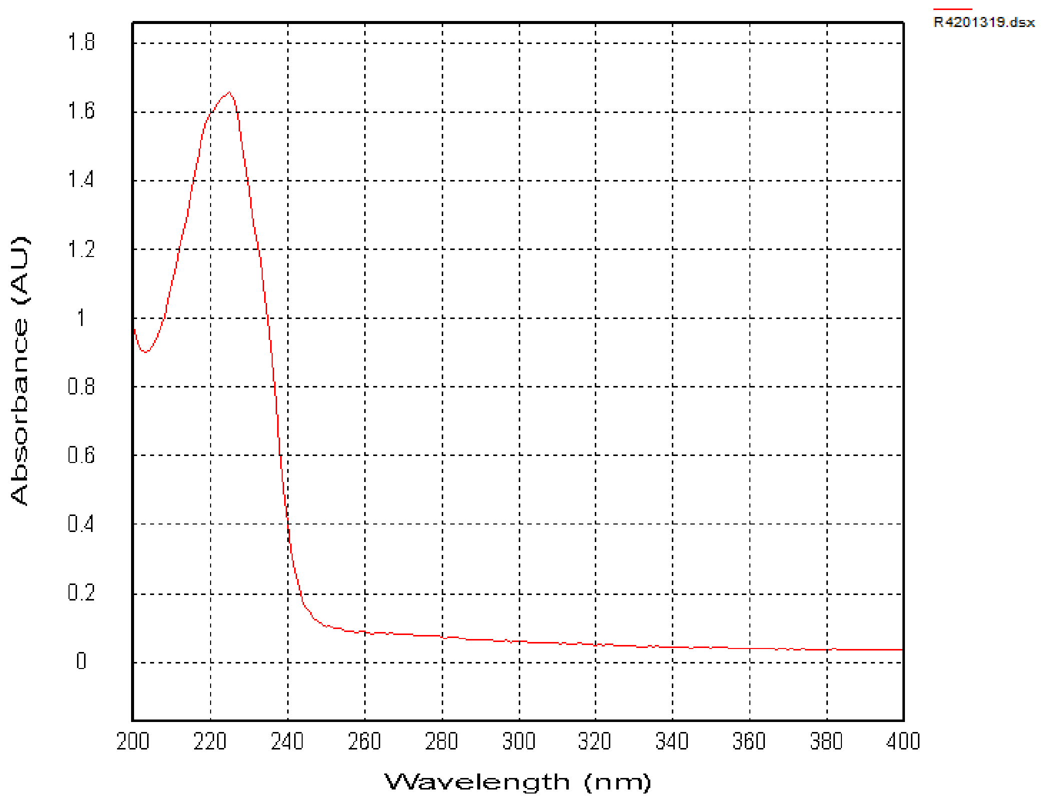

Figure S39. UV spectrum of compound **3** (0.2 mg/mL in MeOH).

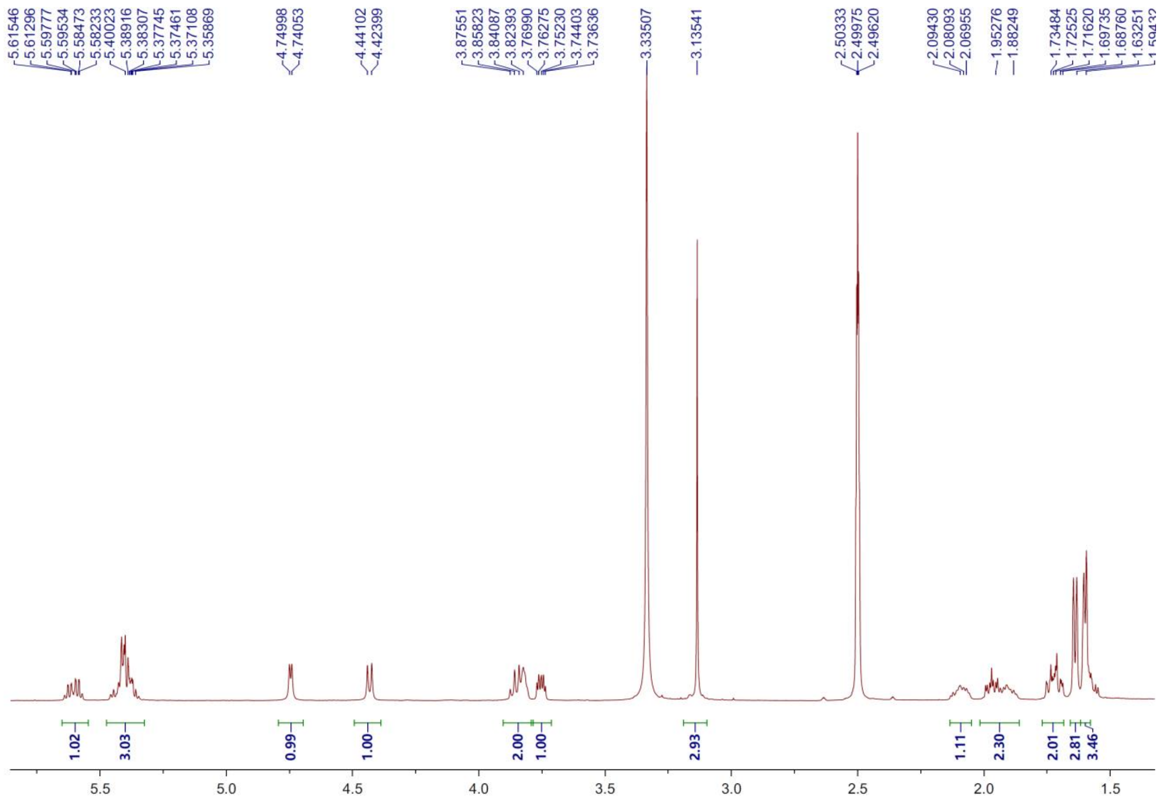

Figure S40. ^{1}H NMR spectrum of compound **4** in DMSO-d_6.

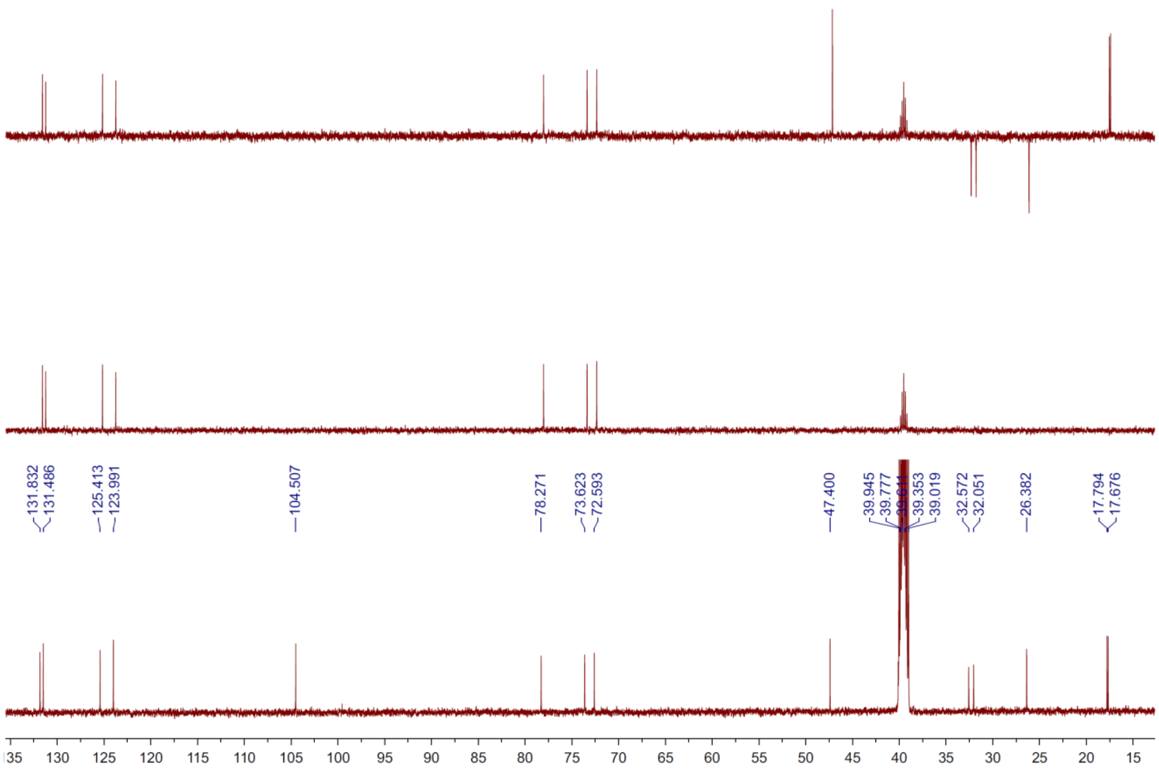

Figure S41. ^{13}C NMR and DEPT spectra of compound **4** in DMSO-d_6.

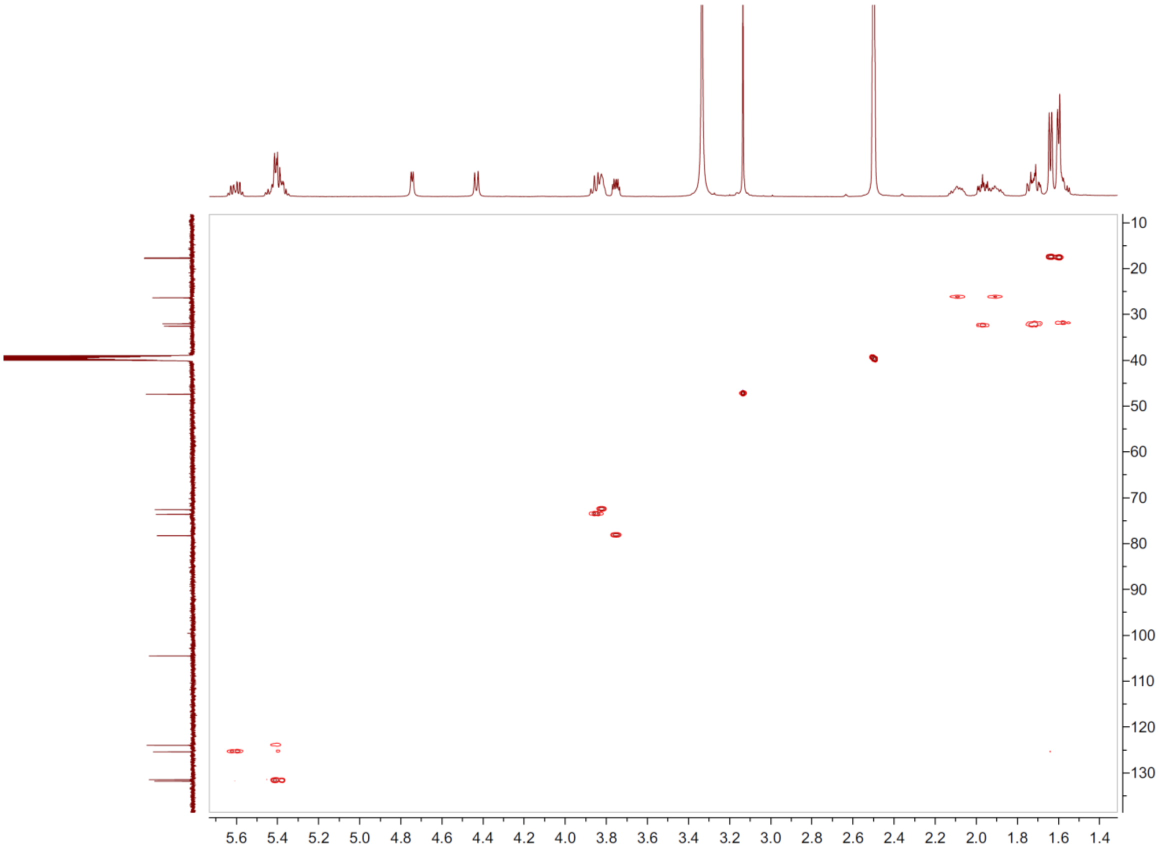

Figure S42. HSQC spectrum of compound **4** in DMSO-d_6.

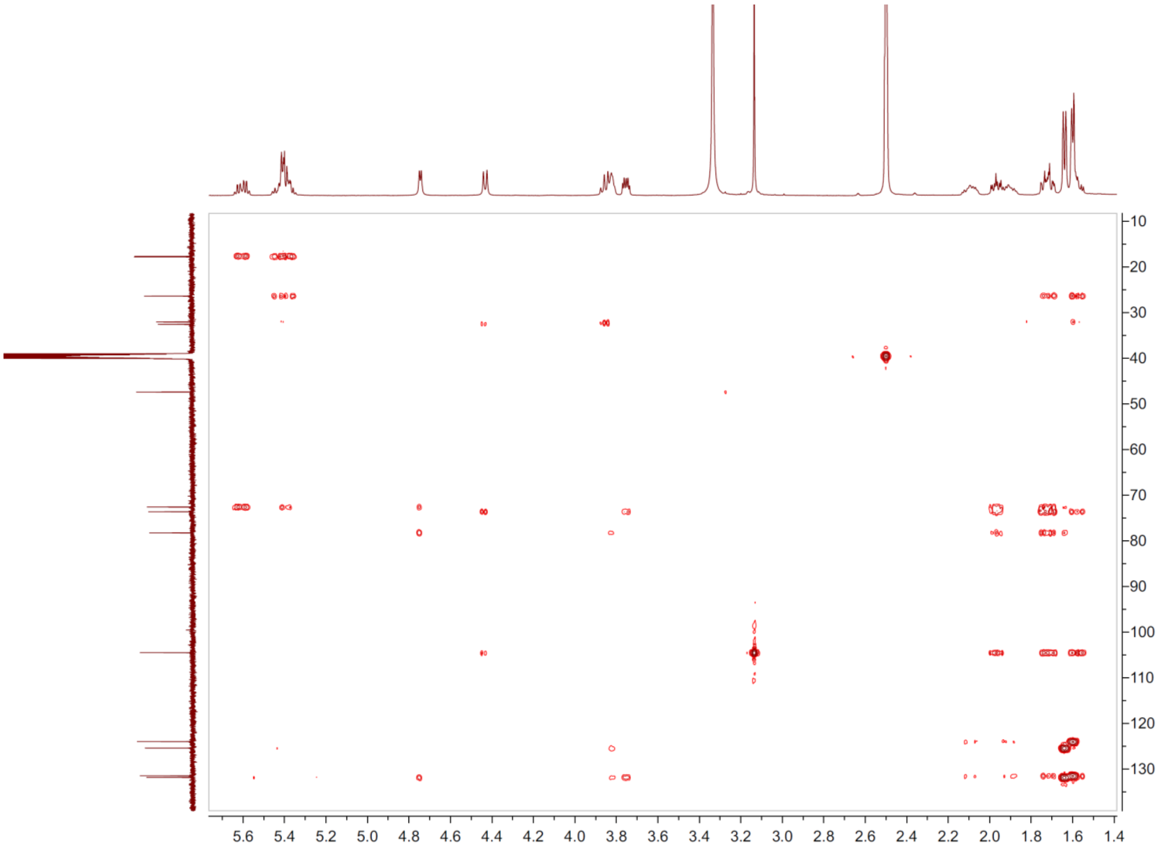

Figure S43. HMBC spectrum of compound **4** in DMSO-d_6.

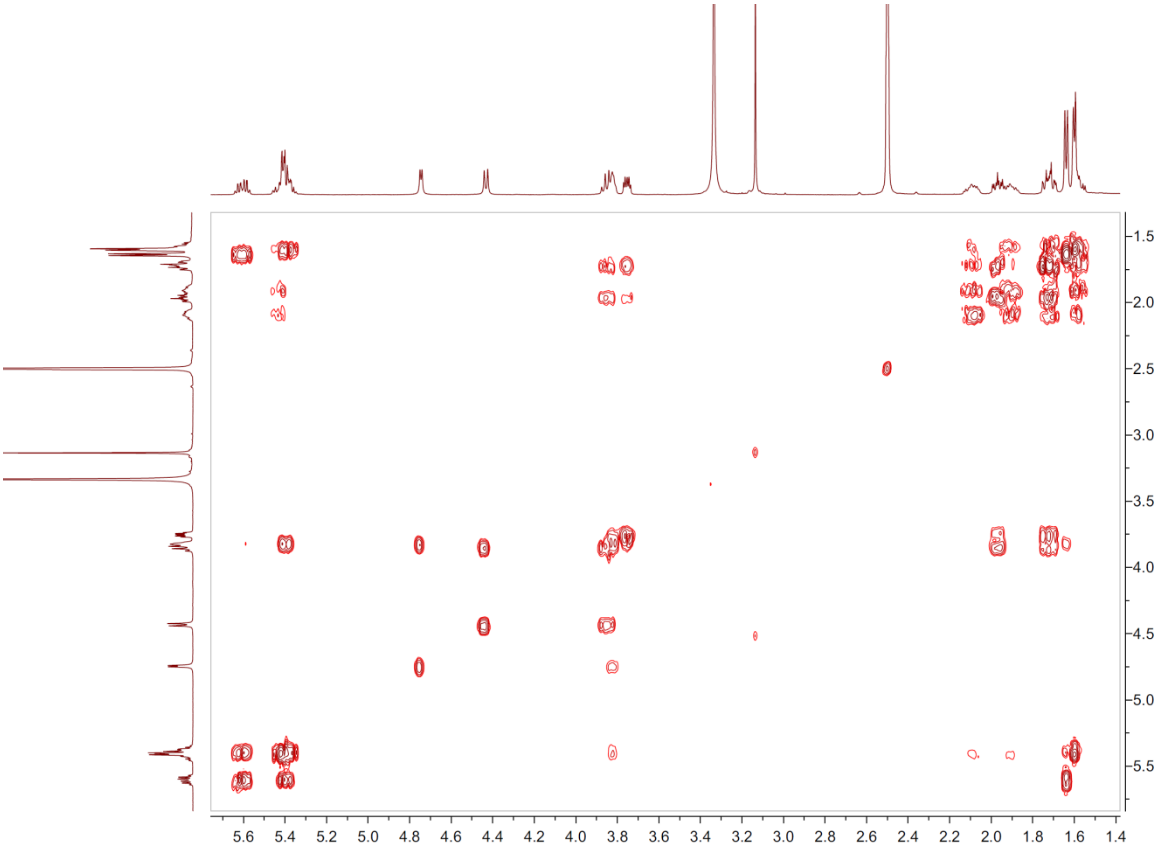

Figure S44. COSY spectrum of compound **4** in DMSO-d_6.

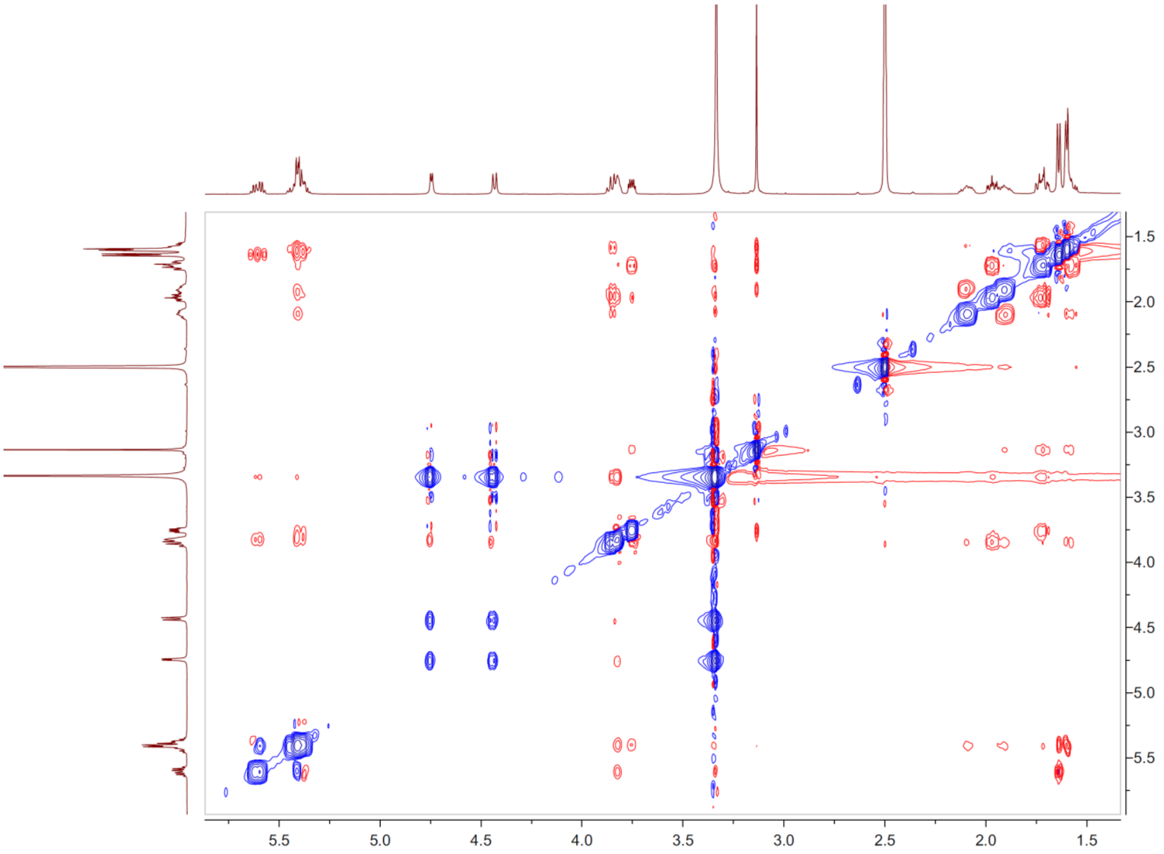

Figure S45. NOESY spectrum of compound **4** in DMSO-d_6.

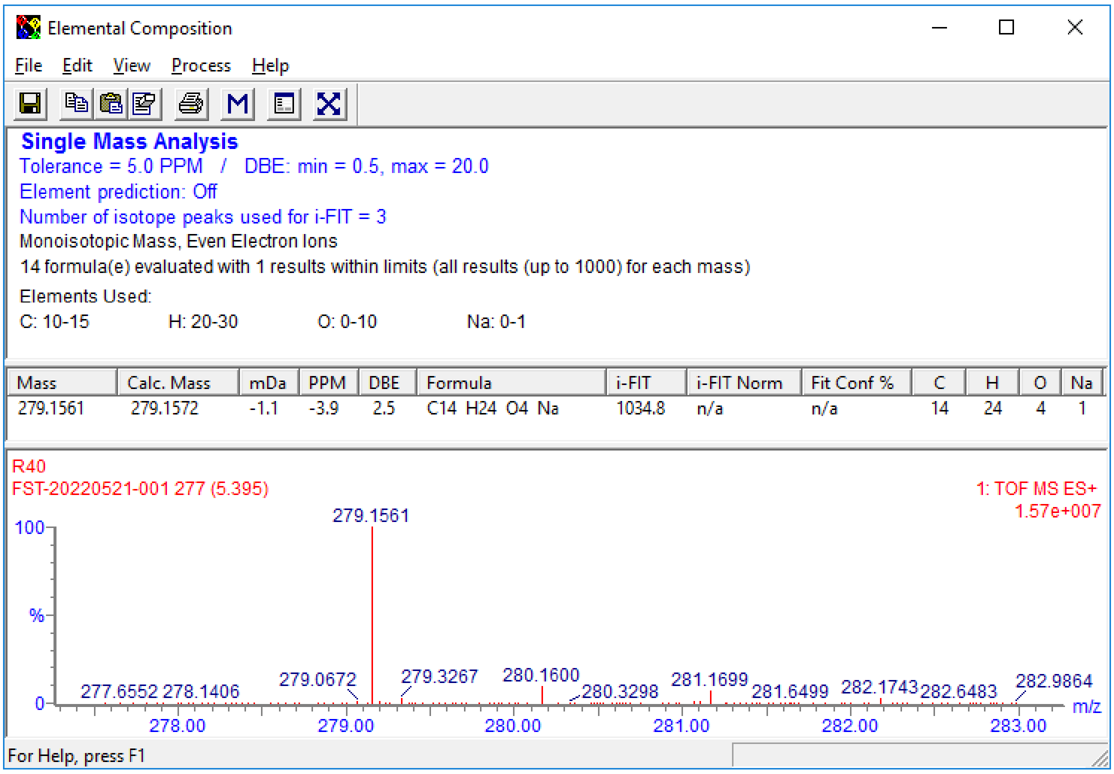

Figure S46. HRESI(+)MS spectrum of compound **4**.

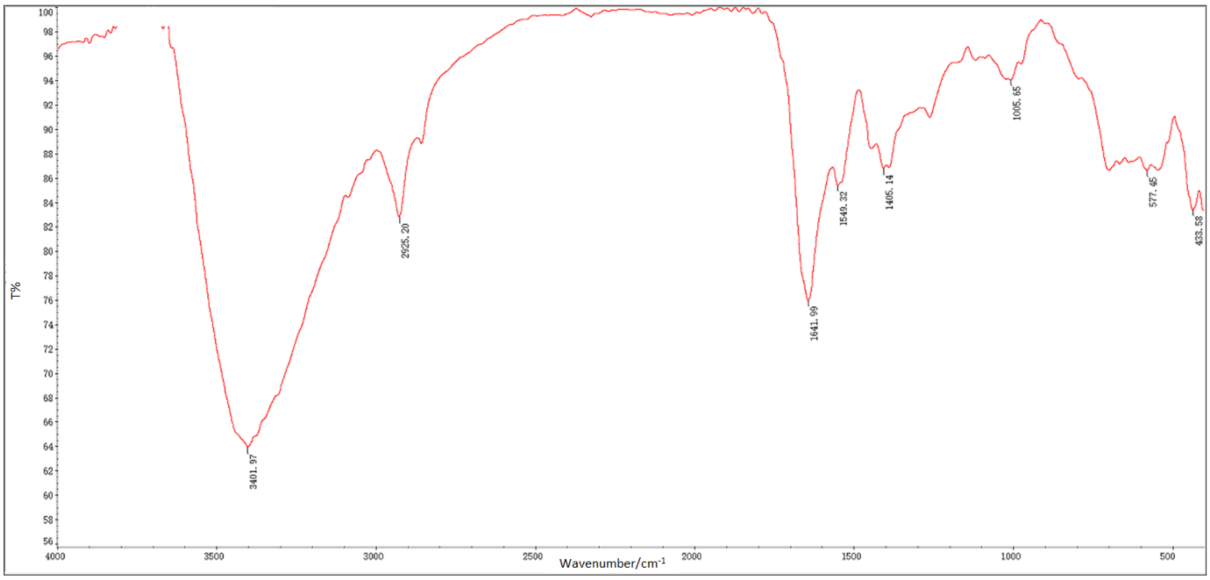

Figure S47. IR spectrum of compound **4**.

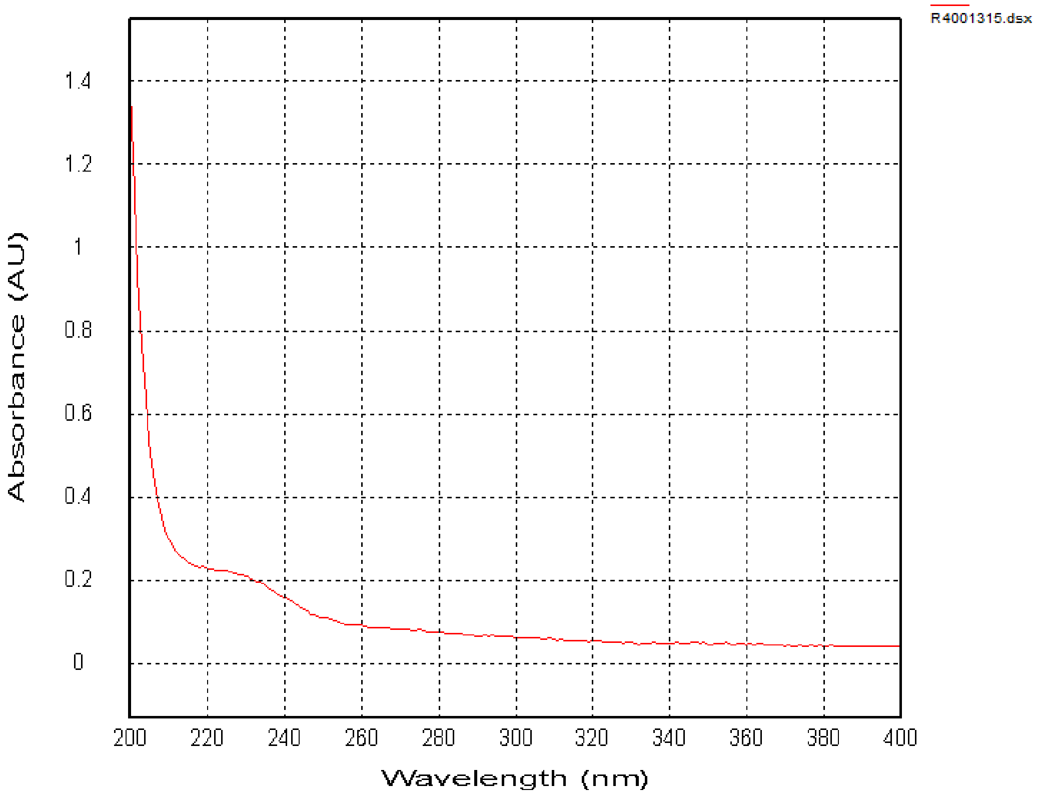

Figure S48. UV spectrum of compound **4** (0.4 mg/mL in MeOH).

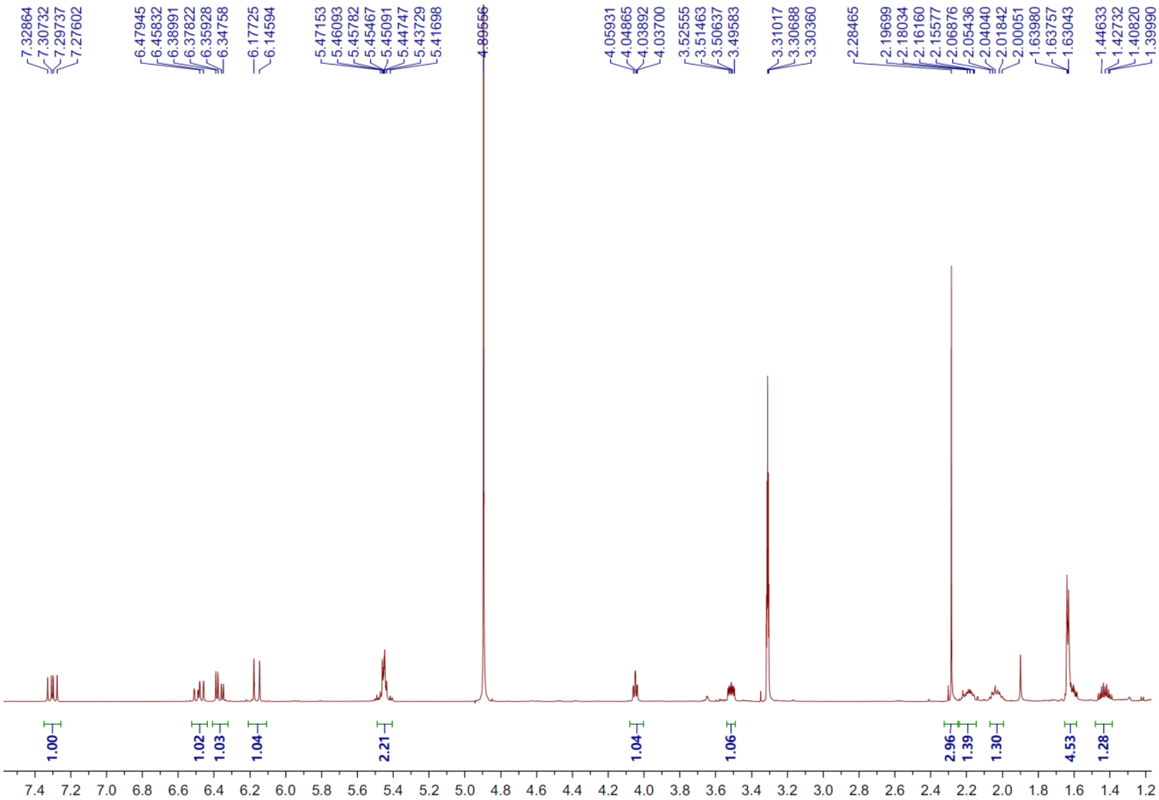

Figure S49. ^{1}H NMR spectrum of compound **5** in CD_3OD.

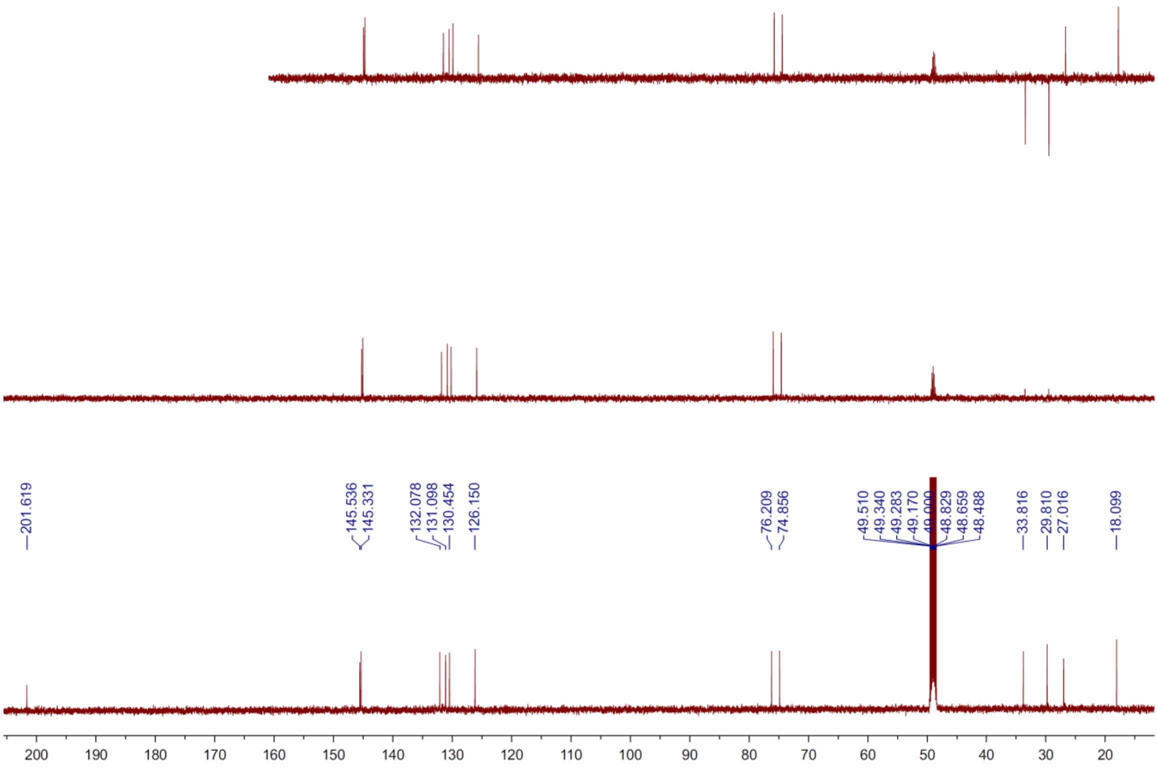

Figure S50. ^{13}C NMR and DEPT spectra of compound **5** in CD_3OD.

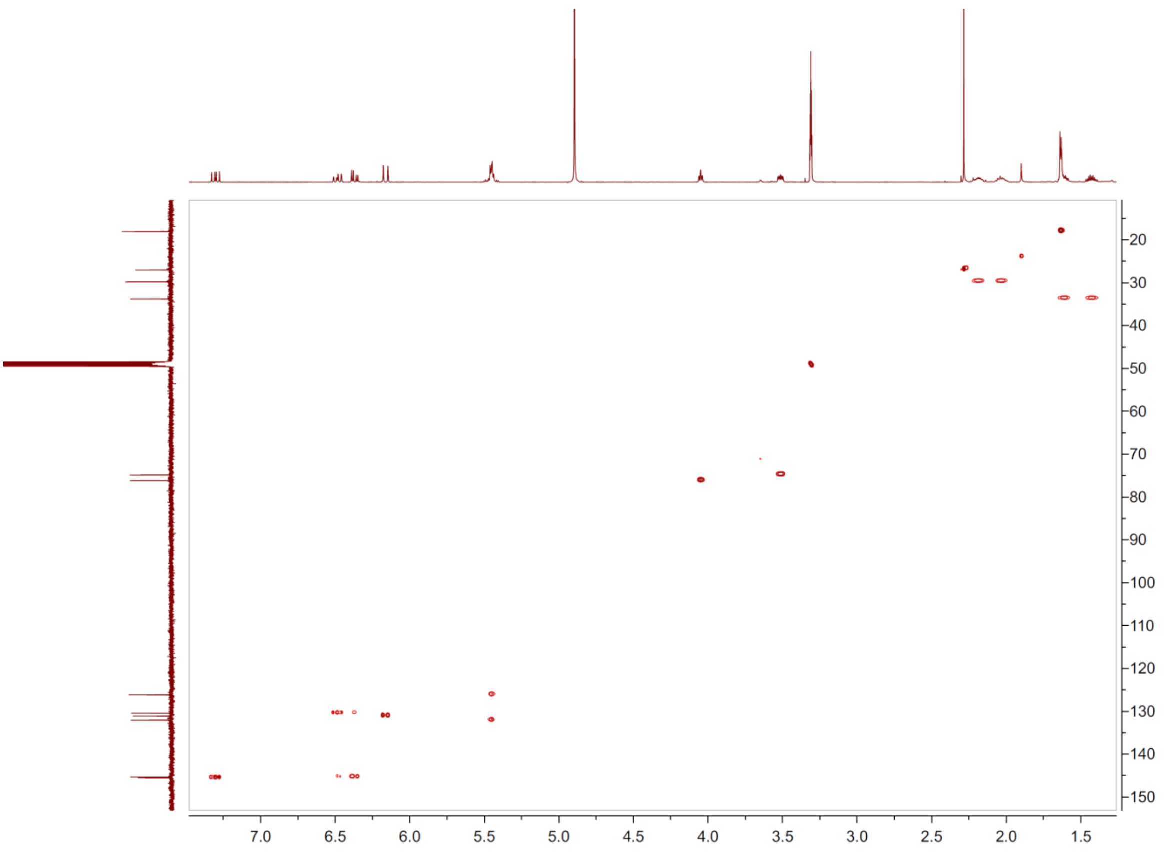

Figure S51. HSQC spectrum of compound **5** in CD_3OD.

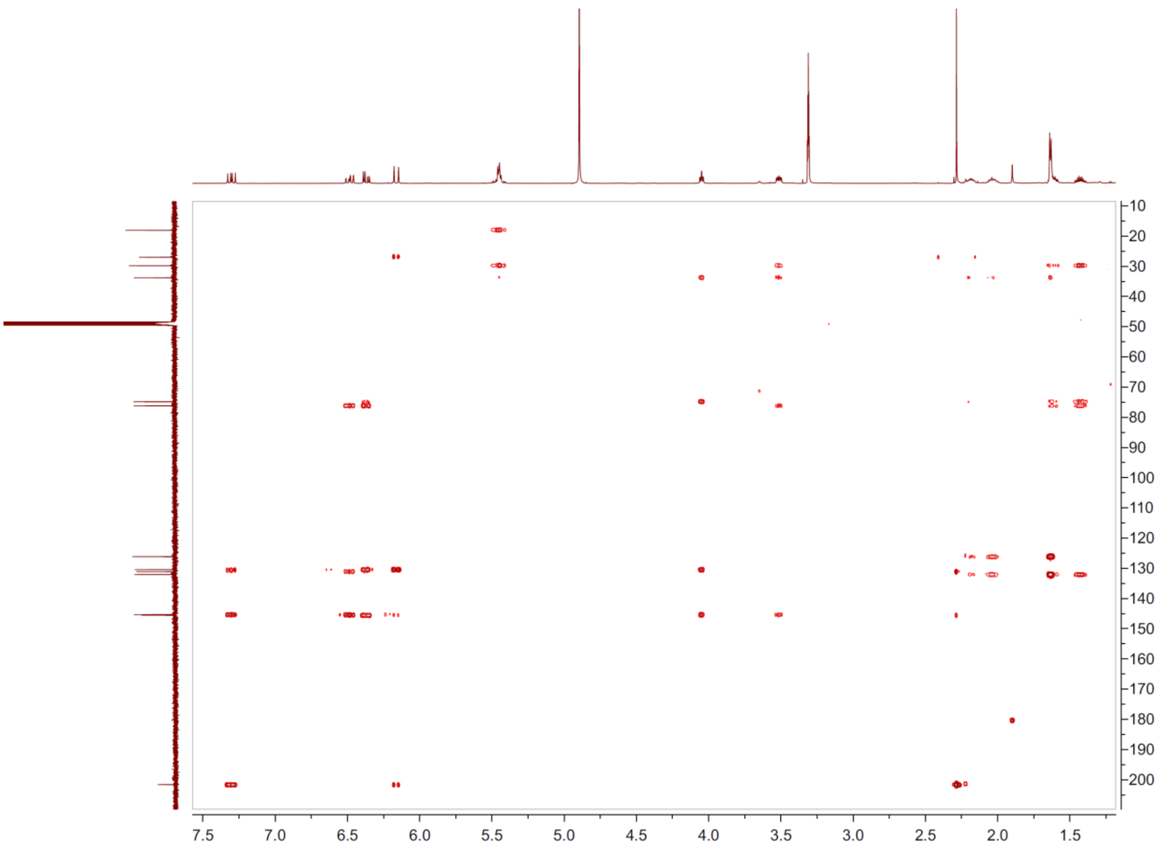

Figure S52. HMBC spectrum of compound **5** in CD_3OD.

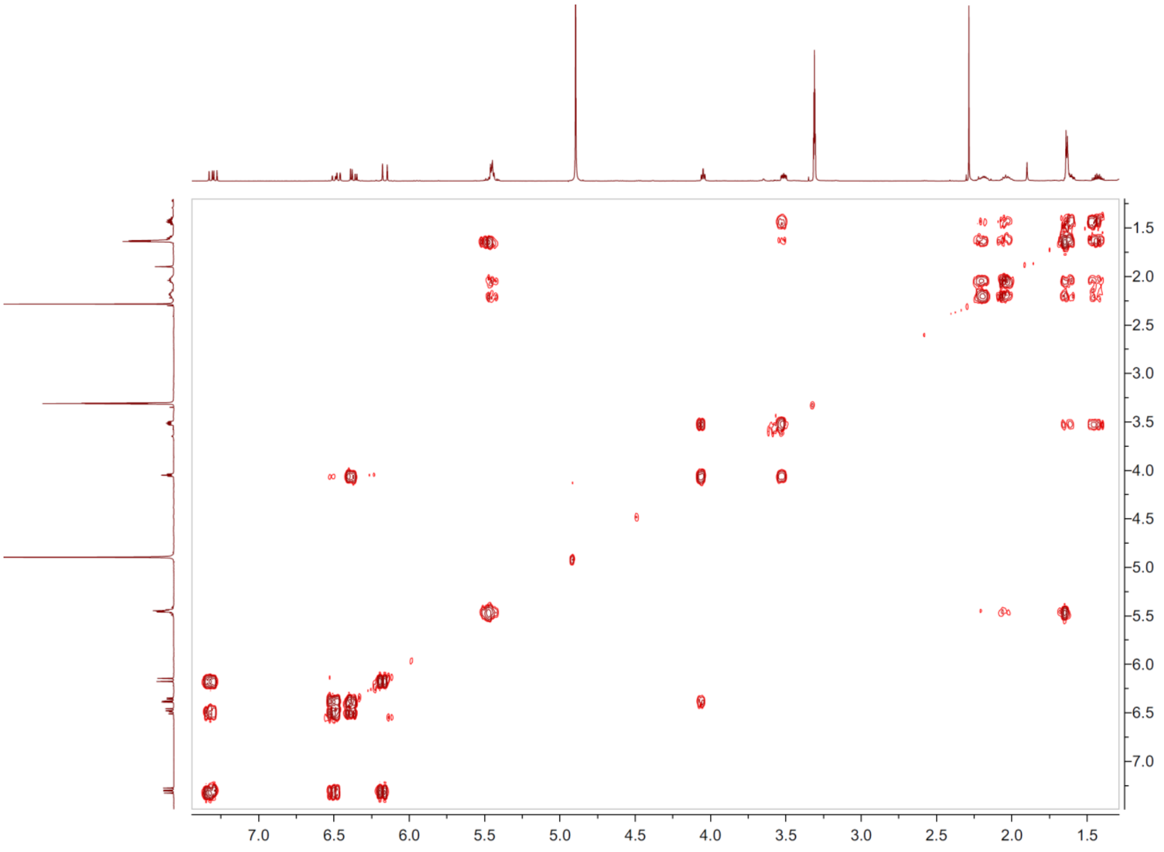

Figure S53. COSY spectrum of compound **5** in CD_3OD.

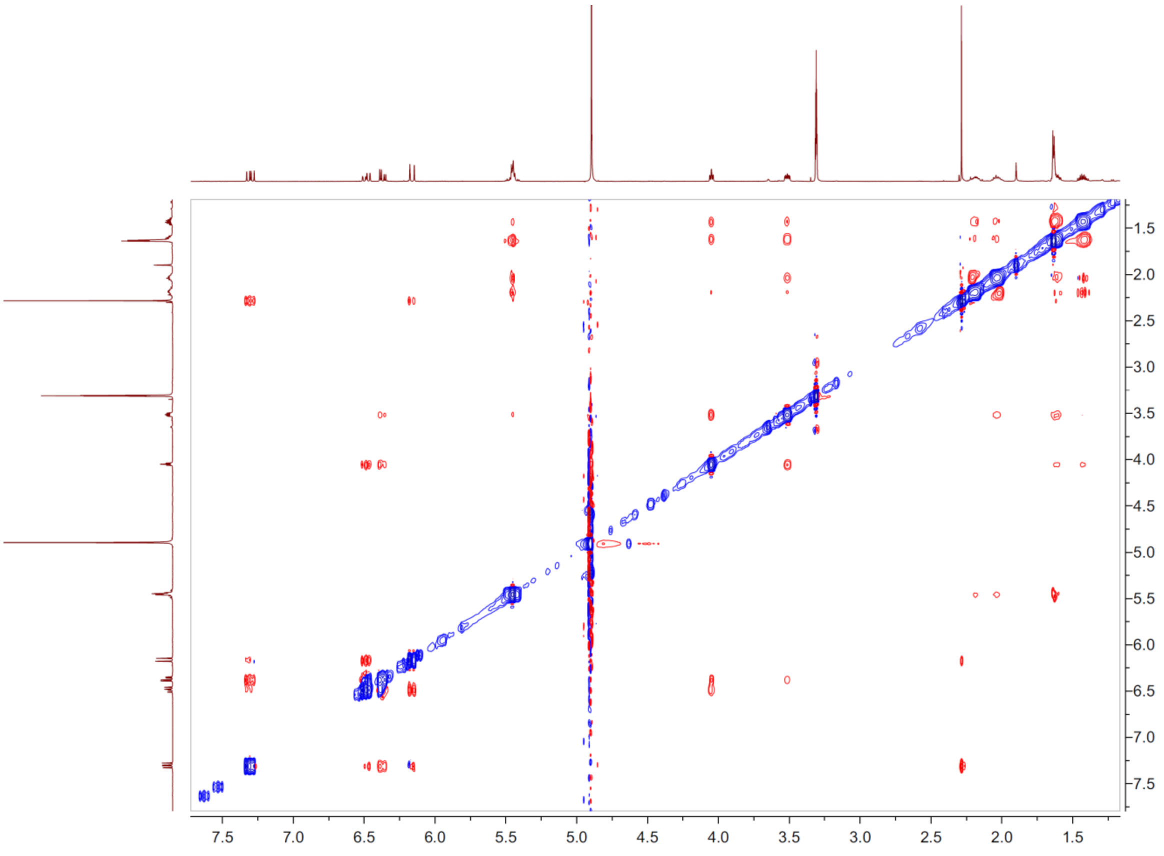

Figure S54. NOESY spectrum of compound **5** in CD_3OD.

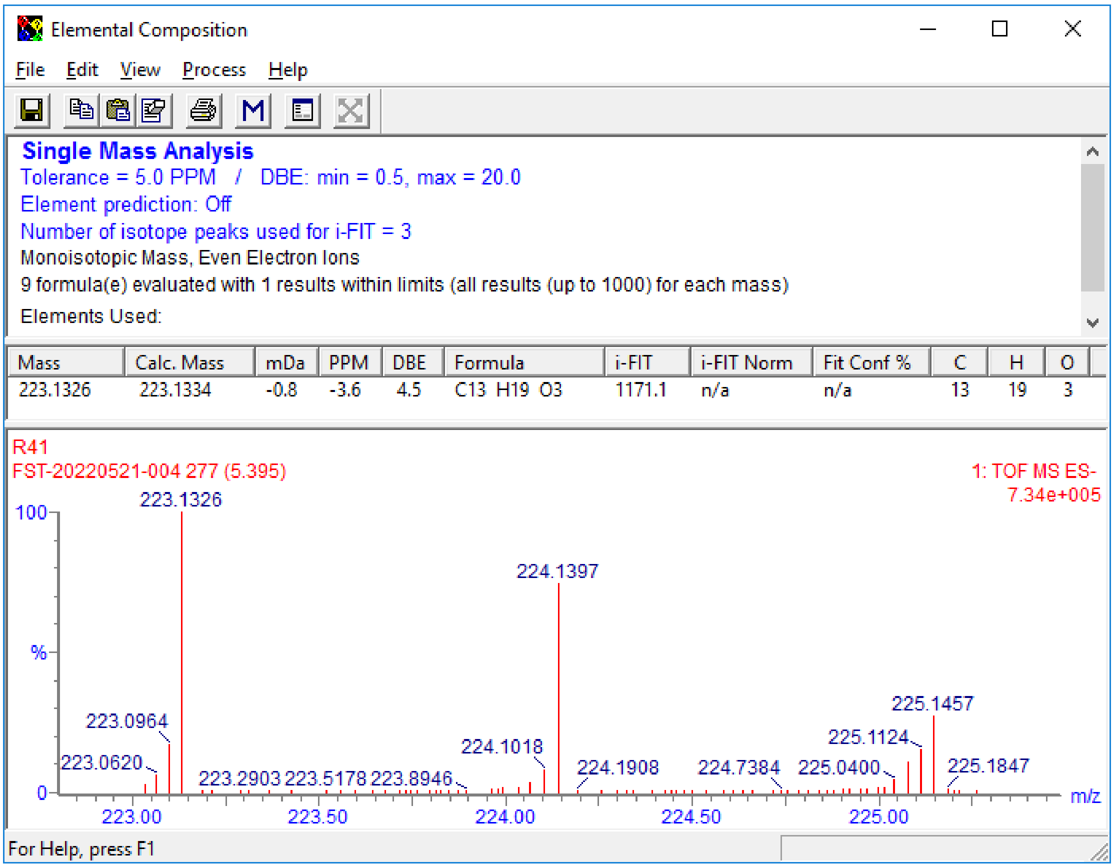

Figure S55. HRESI(-)MS spectrum of compound **5**.

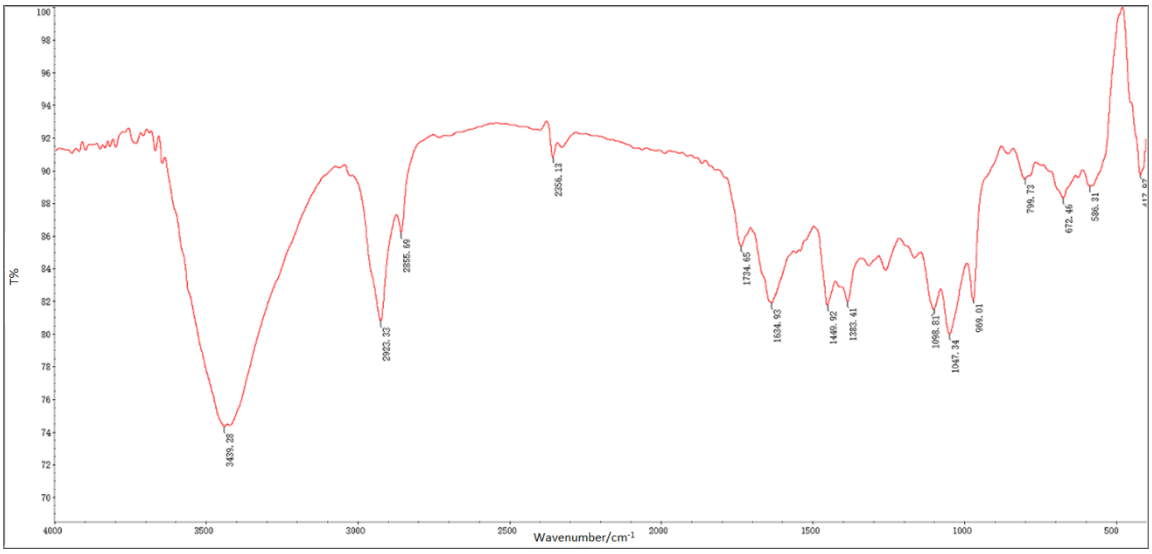

Figure S56. IR spectrum of compound **5**.

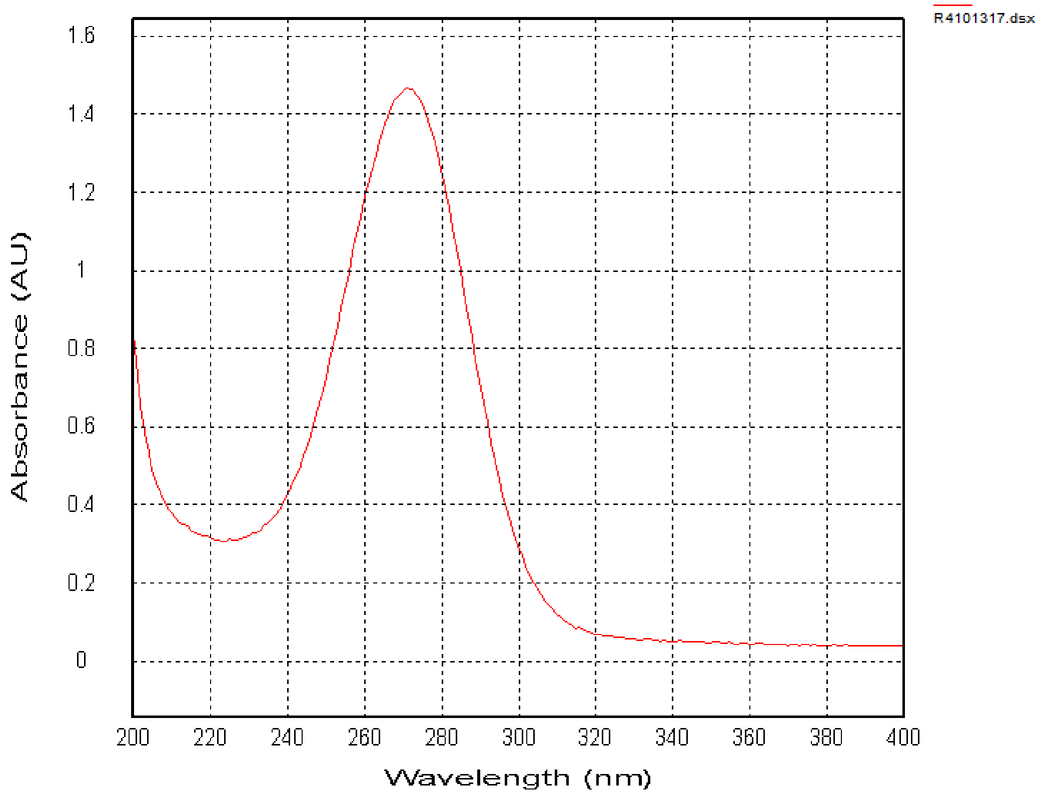

Figure S57. UV spectrum of compound **5** (0.1 mg/mL in MeOH).

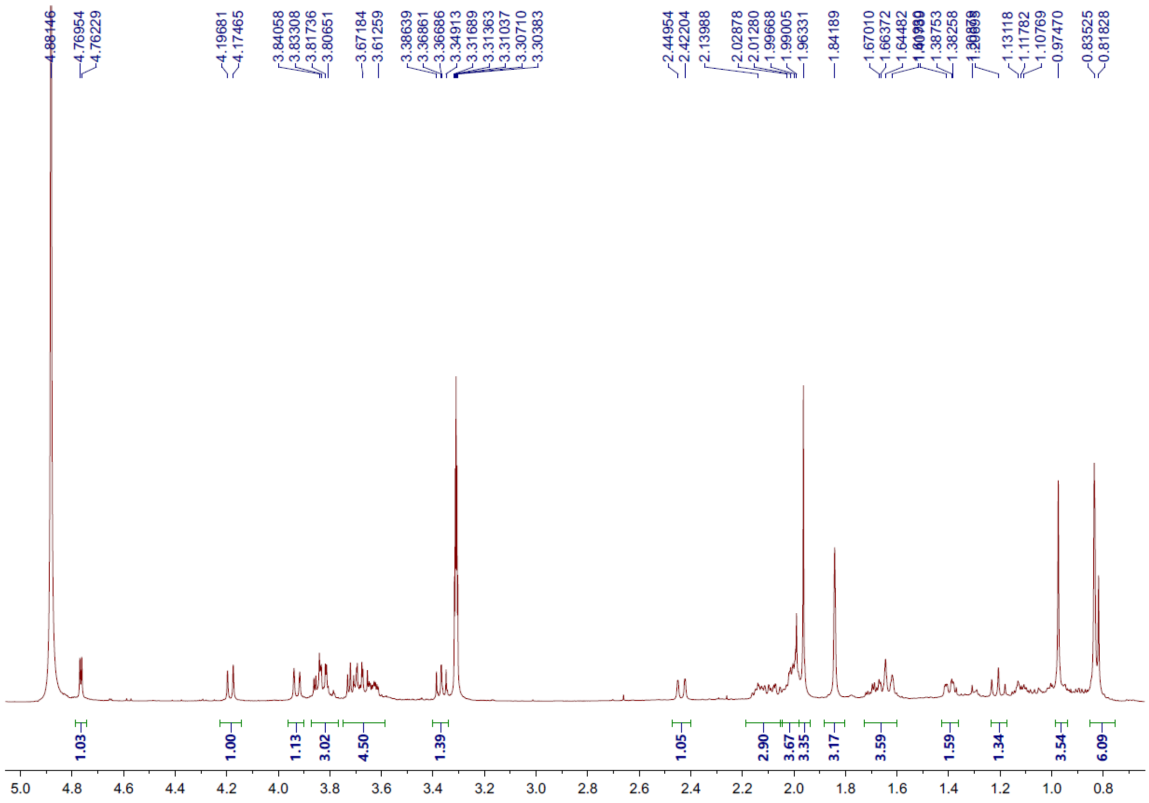

Figure S58. ^{1}H NMR spectrum of compound **6** in CD_3OD.

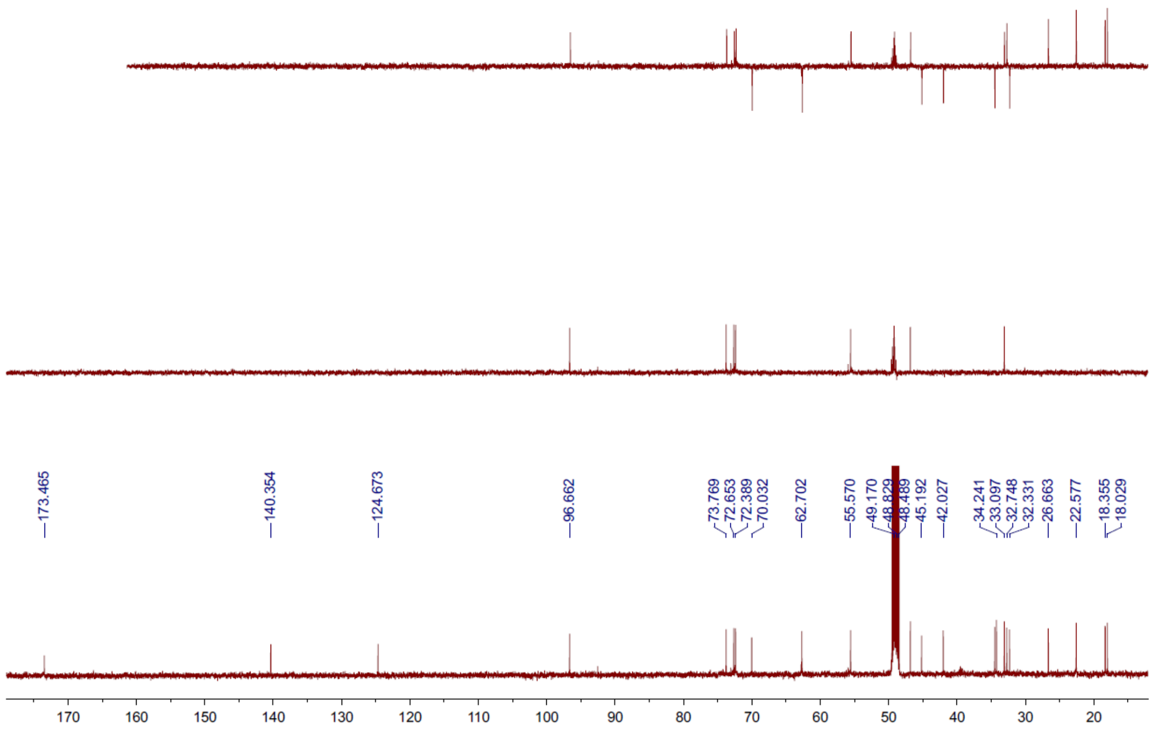

Figure S59. ^{13}C NMR and DEPT spectra of compound **6** in CD_3OD.

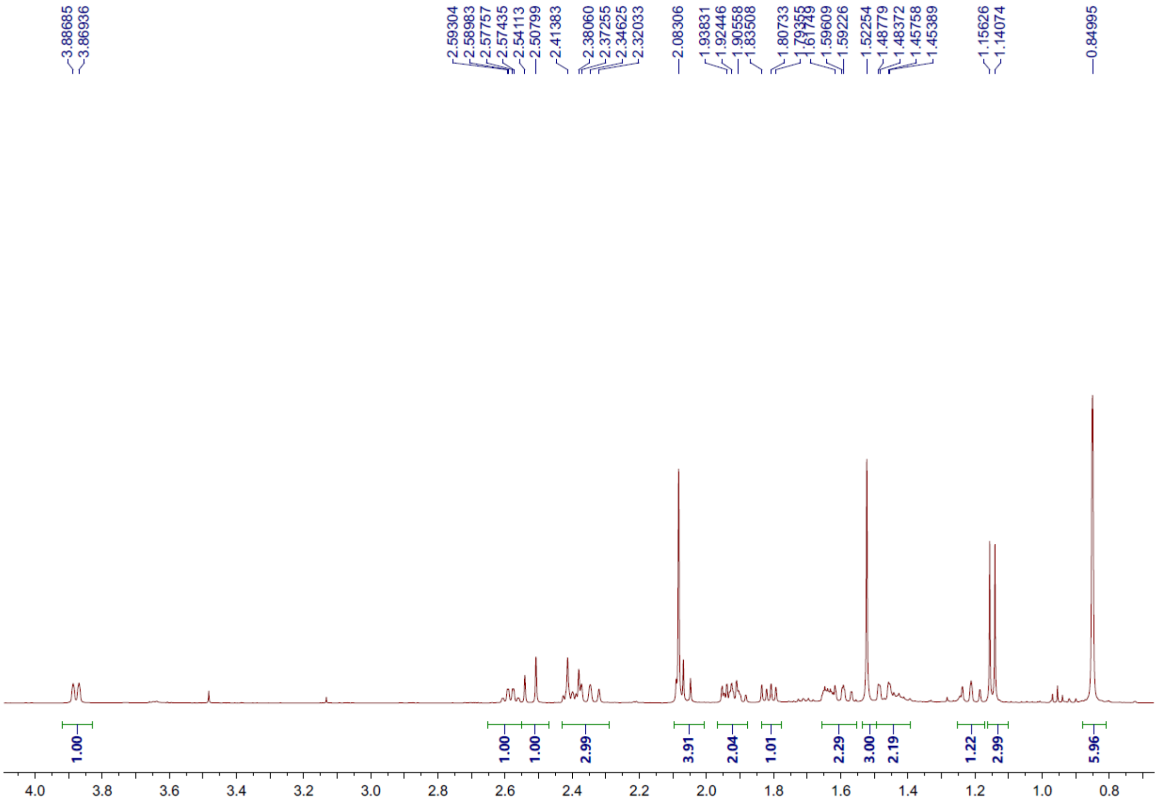

Figure S60. ^{1}H NMR spectrum of compound **7** in $CDCl_3$.

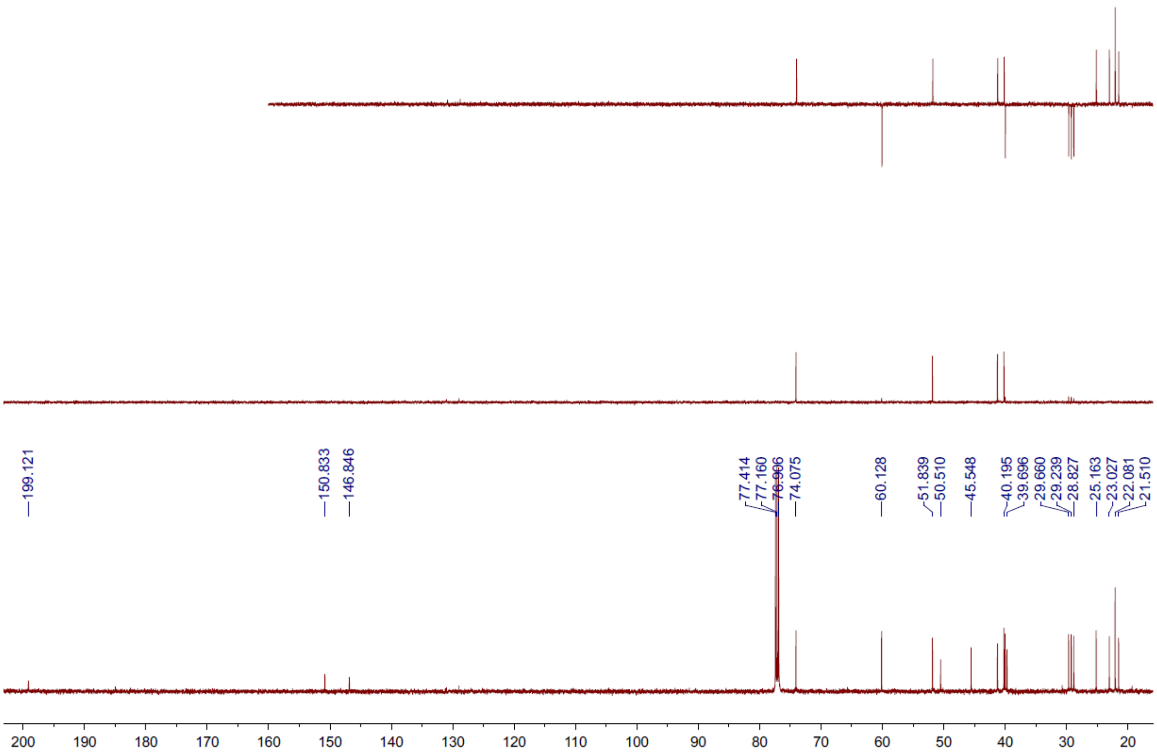

Figure S61. ^{13}C NMR and DEPT spectra of compound **7** in $CDCl_3$.
